# Supplementary material for: Severe COVID-19 patients have impaired plasmacytoid dendritic cell-mediated control of SARS-CoV-2
Source: Nat Commun. 2023 Feb 8;14:694. doi: 10.1038/s41467-023-36140-9 (PMC9907212; doi:10.1038/s41467-023-36140-9)
Supplement: Supplementary file 1 — Supplementary Information [file 41467_2023_36140_MOESM1_ESM.pdf]

Severe COVID-19 patients have impaired plasmacytoid dendritic cell-mediated control of SARS-CoV-2

Manon Venet[1*], Margarida Sa Ribeiro[1*], Elodie Décembre[1*], Alicia Bellomo[1*], Garima Joshi[1], Célia Nuovo[1], Marine Villard[1], David Cluet[2], Magali Perret[1], Rémi Pescamona[1], Helena Paidassi[1], Thierry Walzer[1], Omran Allatif[1], Alexandre Belot[1], Sophie Trouillet-Assant[1], Emiliano P. Ricci[2], and Marlène Dreux[1,#]

[1] CIRI, Inserm, U1111, Université Claude Bernard Lyon 1, CNRS, UMR5308, École Normale Supérieure de Lyon, Univ Lyon, F-69007, LYON, France
[2] Laboratory of Biology and Modeling of the Cell, Université de Lyon, ENS de Lyon, Université Claude Bernard, CNRS UMR 5239, Inserm U1293, Lyon, France.
[#] Corresponding author/lead contact; email: marlene.dreux@ens-lyon.fr, Tel.: + +33 426 23 38 34.

[*] These authors contributed equally.

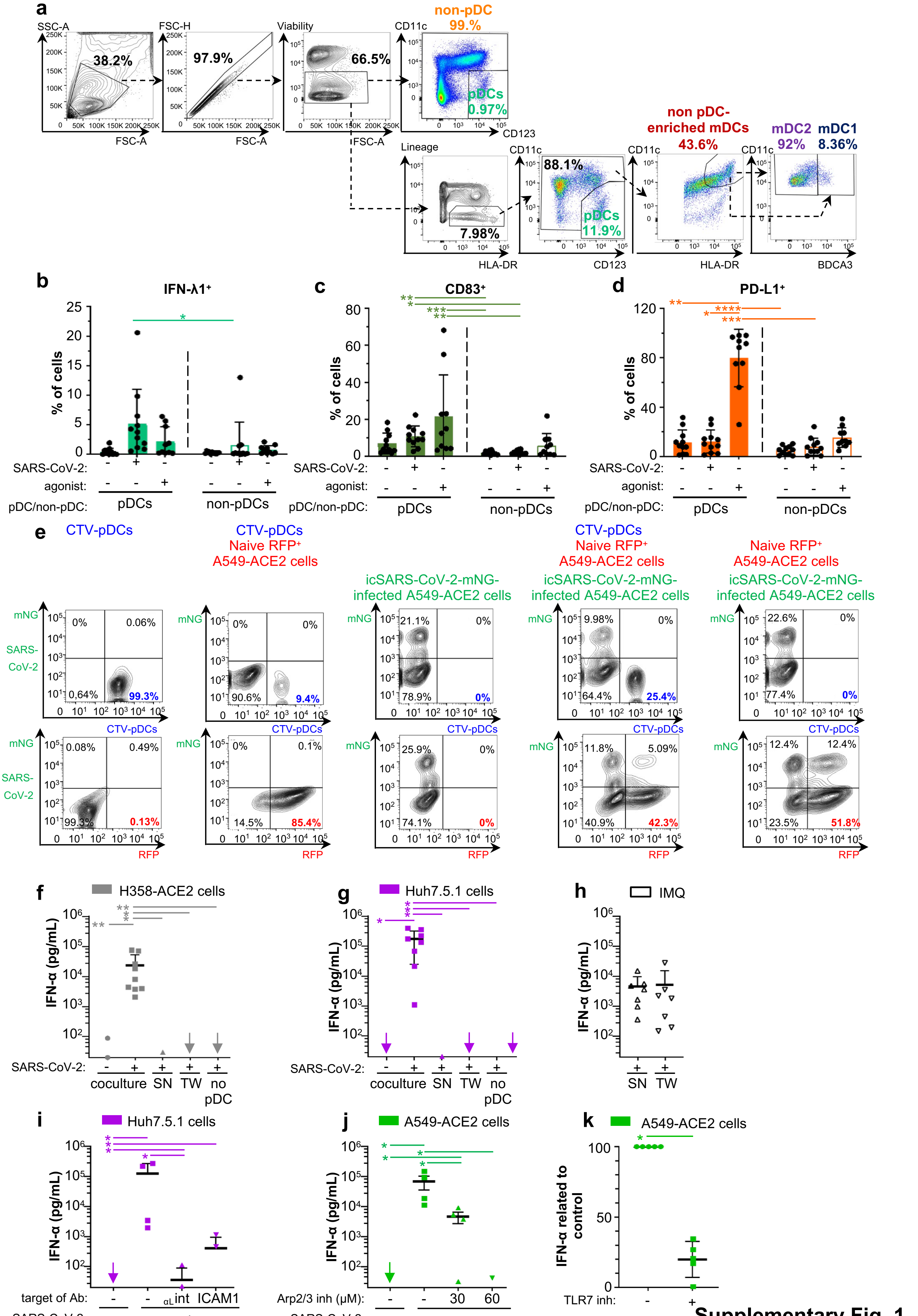

Supplementary Fig. 1

Supplementary Fig. 1. Assessment of pDC response and their non-permissivity to infection. a, Representative flow cytometric analysis of pDC gating strategy from PBMCs: pDCs gated as singulets$^+$ live cells$^+$ CD11c$^-$ CD123$^+$; non-pDC PBMCs gated as singulets$^+$ live cells$^+$ CD11c$^+$ CD123$^-$; non-pDC enriched mDCs gated as singulets$^+$ live cells$^+$ lineage [CD3, CD19, CD20, CD56, CD14, CD16]$^-$, HLA-DR$^+$, CD123$^-$ and CD11c$^+$ and CD11c$^+$/BDCA3$^+$ *versus* CD11c$^+$/BDCA3$^-$ for mDC1 and mDC2 subsets, respectively. **b-d,** PBMCs from healthy donors were cocultured with SARS-CoV-2-infected or uninfected A549-ACE2 cells or treated with agonists as in **Fig. 1c-d, b,** Frequency of positive cells for IFN-λ1 (**b**), CD83 (**c**) and PD-L1 (**d**) in populations gated as pDCs (left bars) non-pDC PBMCs (right bars). Means ± SD; n=10-11 independent experiments using distinct healthy donors. **e**, A549-ACE2 cells were infected by icSARS-CoV-2-mNG for 24 hours prior to coculture with isolated CTV-stained pDCs and/or RFP$^+$A549-ACE2 uninfected cells for 48 hours. Viral transmission from icSARS-CoV-2-mNG-infected cells to pDCs *versus* RFP$^+$A549-ACE2-uninfected cells was assessed by flow cytometry. Results are expressed as the percentage of infected cells (mNG$^+$) in the living cell populations gated as pDCs (CTV$^+$) and non-pDCs (CTV$^-$/RFP$^+$). Means ± SD; dot plots are representative of n=4-5 independent experiments. **f-k,** Quantification of IFN-α in SNs of pDCs cocultured with the indicated cell types infected or not by SARS-CoV-2. **f-g,** pDCs were cultured with infected cells, either in direct contact (coculture) or physically separated by the semi-permeable membrane of transwell [TW], or treated with supernatants [SN] from the corresponding SARS-CoV-2-infected cells. IFN-α concentration was also determined in the SN of SARS-CoV-2-infected cells cultured without pDC [no pDC]. **h,** In parallel experiments, treatment of pDCs with TLR7 agonist imiquimod [IMQ, 20 μM] in coculture *versus* transwell settings served as positive control. Means ± SD; n=3-9 independent experiments. **i-k,** Quantification of IFN-α in SN of pDCs cocultured with SARS-CoV-2-infected cells *(*A549-ACE2 and Huh7.5.1 cells as indicated) and treated or not with blocking antibodies against α_L-integrin and ICAM-1 at 10 μg/mL (**i**) or ARP2/3 inhibitor (CK-666) at the indicated concentrations (**j**), or with TLR7 inhibitor (IRS661; 0.35 μM, **k**). Means ± SD; Dots represent n=4-5 independent experiments. For all panels, when appropriated, p-values are indicated as follows: ≤0.05 as *; ≤0.005 as **; ≤0.0005 as ***; and ≤0.00005 as ****. Source data are provided as a Source Data file.

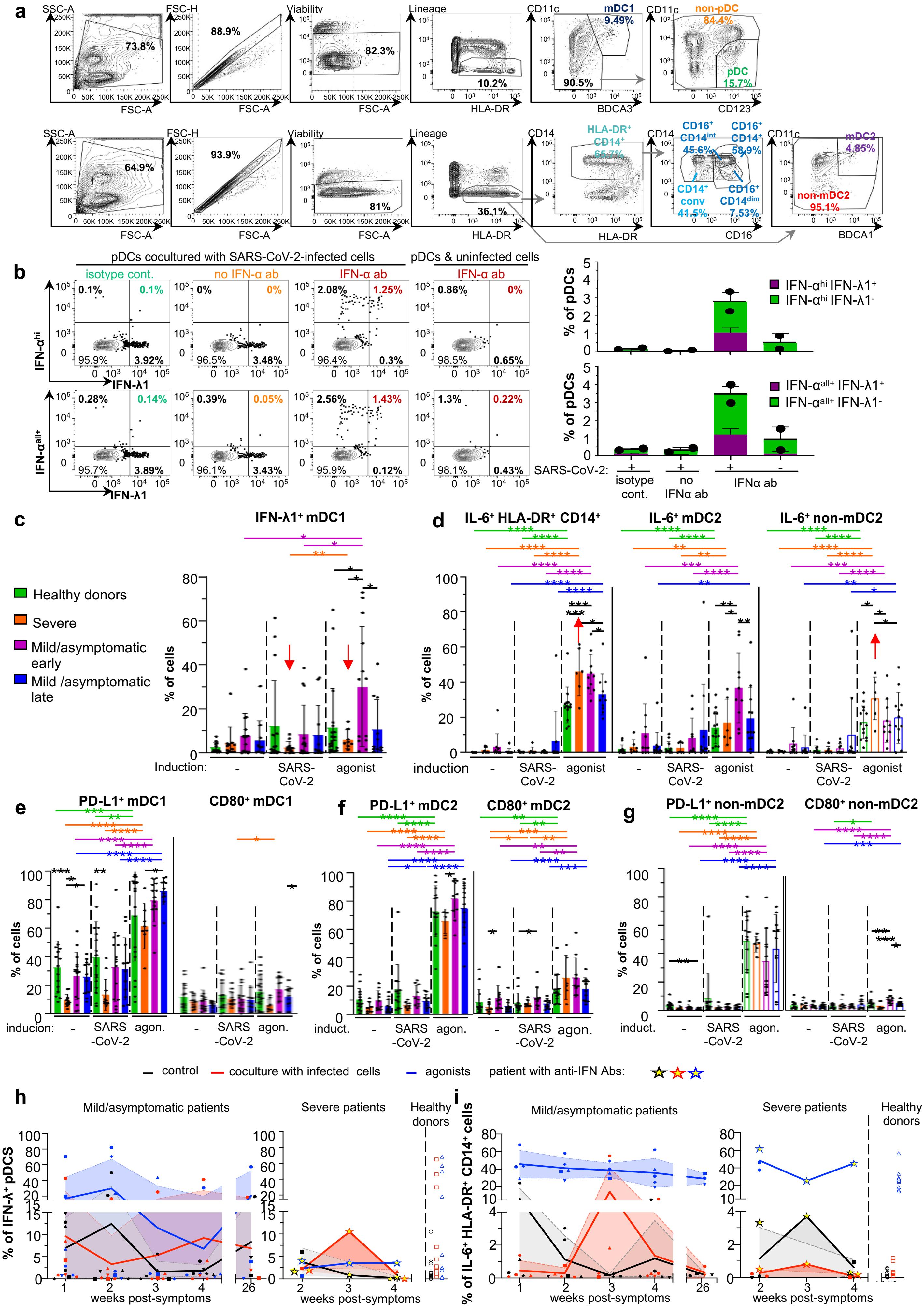

Supplementary Fig. 2

Supplementary Fig. 2. Responsiveness of PBMCs from COVID-19 patients to SARS-CoV-2-infected cells *versus* agonist, related to Fig. 2. PBMCs issued from the indicated groups of patients *(Healthy donors, Severe, Mild/asymptomatic early, Mild/asymptomatic late*, listed in **Table 1 and Supplementary Table 1**) were cocultured for 14-16 hours with SARS-CoV-2-infected or uninfected A549-ACE2 cells or treated with agonists as in **Fig. 2b-h**. **a,** Gating strategies for mDC1 and pDC/non-pDC (upper panels), and mDC2/non-mDC2/HLA-DR^+ $CD14^+$ monocytes (lower panels). **b,** Assessment of specific IFN-α detection by comparing the full antibody panel as in **Fig. 2b-h**. Cells were cocultured as in **Fig. 2b-h** and similarly analyzed using the same antibody panel [IFN-α ab] *versus* replacing only IFN-α antibody by IgG1 isotype control [isotype cont.] *versus* omitting only IFN-α antibody [no IFN-α ab]. The representative dot blots are presented in Left panels and the quantification in right panels; means; dots represent n=2 independent experiments. **c-g,** Quantification of the frequency of cells positive for IFN-λ1 in gated mDC1 (**c**), IL-6 in gated HLA-DR^+ $CD14^+$ monocytes, mDC2, non-mDC2 (**d**), CD80 and PD-L1 in gated mDC1 (**e**), mDC2 (**f**) and non-mDC2 (**g**). The numbers of included patient samples are listed in the **Table 1** and **Supplementary Table 1**. **c-g,** Error bars represent the means ± SD; each dot represents the level determined for PBMCs from one individual patients in each group, or healthy donors. The p-values are indicated as follows: ≤0.05 as *; ≤0.005 as **; ≤0.0005 as ***; and ≤0.00005 as ****. **h-i,** Kinetic analysis of pDC IFN-$\lambda1^+$ response (**h**) and HLA-DR^+ $CD14^+$ $IL6^+$ in PBMCs (**i**) collected from *Mild/asymptomatic* (left panel), *Severe* patients (middle panel) and *healthy donors* (right panel), upon *ex vivo* stimulation with SARS-CoV-2-infected cells (red) agonist (blue) *versus* control cells (black). As in **Fig. 2h,** dots for the *Severe* patient with circulating anti-IFN antibodies (see description in **Supplementary Table 1**) are represented by yellow-centered stars. Patient PBMCs were collected from symptom onset and results correspond the timeframes as follows: week 1= [Days 1-8]; 2= [Days 8-15]; 3= [Days 15-22]; 4= [Days 22-30]. Means (coloured lines) and errors (coloured areas) are indicated (n=20-24 analysed patients). Source data are provided as a Source Data file.

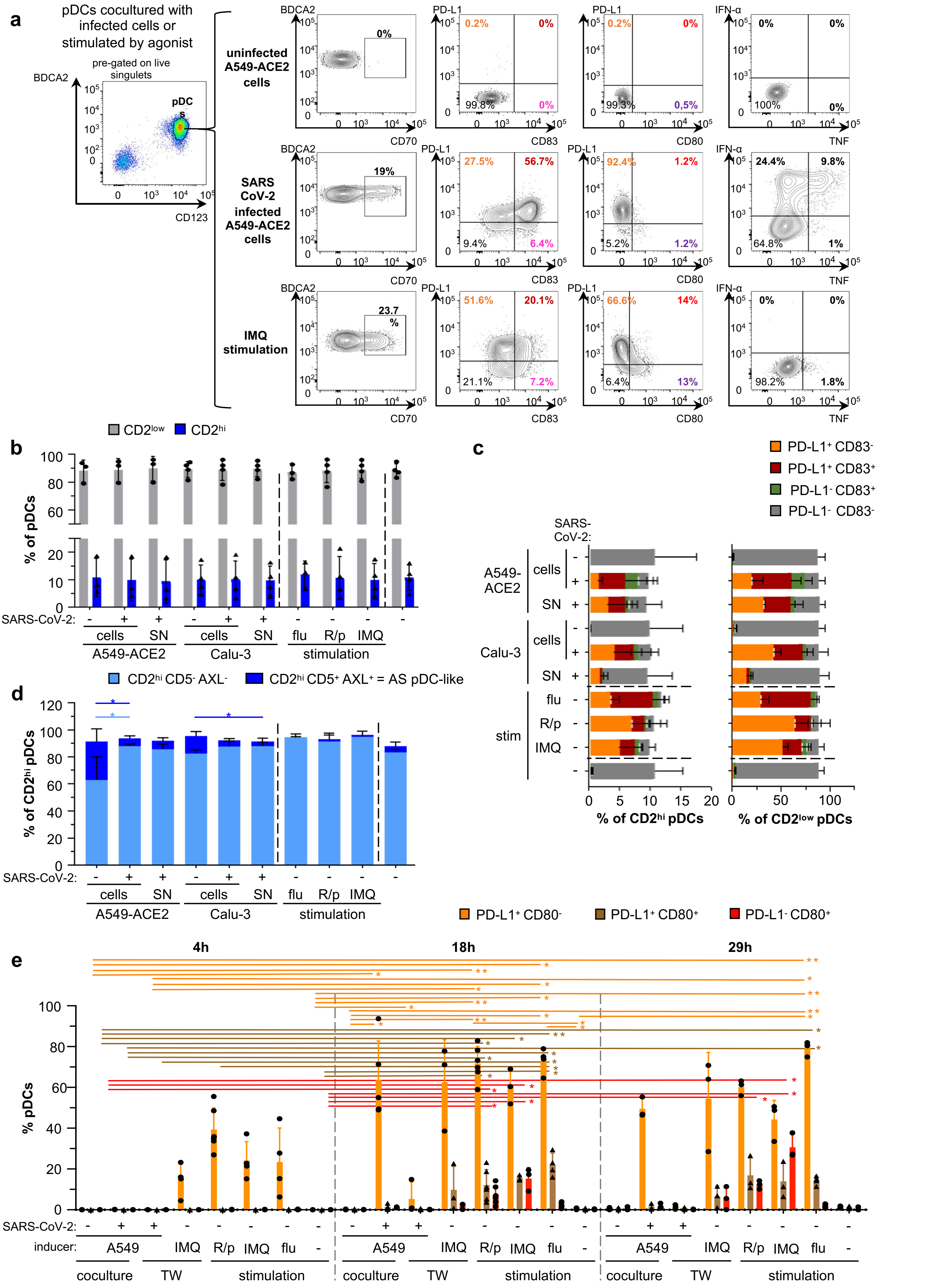

Supplementary Fig. 3

Supplementary Fig. 3. Activation profile and diversification of pDCs in response to coculture with SARS-CoV-2-infected cells, as compared to synthetic agonist stimulation. Purified human pDCs were cocultured with uninfected [-] or SARS-CoV-2-infected [+] A549-ACE2 or Calu-3 cells, or cell-free SN collected from SARS-CoV-2-infected cells (48 hours of infection prior to coculture) or were stimulated with influenza virus [flu], R848/polyI:C [R/p] or imiquimod [IMQ], as in **Fig. 3a-b, d**. The samples were collected for FACS analysis at 14-16 hours of cocultures, and at the indicated times (**e**). **a,** Gating strategies used to determine the activation profile and diversification of pDCs. Dot plots showing the expression of BDCA2/CD70, PD-L1/CD83, PD-L1/CD80, IFN-α/TNF are representative of n=3-5 independent experiments. **b-e**, Quantification by flow cytometry of the frequency of gated pDCs as: $CD2^{hi}$ and $CD2^{low}$ subsets (**b**), PD-L1/CD83 positivity in $CD2^{hi}$ and $CD2^{low}$ subsets (**c**), pDC subsets defined by CD2, CD5 and AXL (**d**), and of subset assigned by PD-L1/CD80 and determined at the coculture duration indicated at the top of the graph (**e**). Bars represent means ± SD; n=3-5; The data were analyzed using Kruskal-Wallis Global test and p-values were calculated with Tukey and Kramer test; ns: p >0,05; *p<0,05; **p<0,005. Source data are provided as a Source Data file.

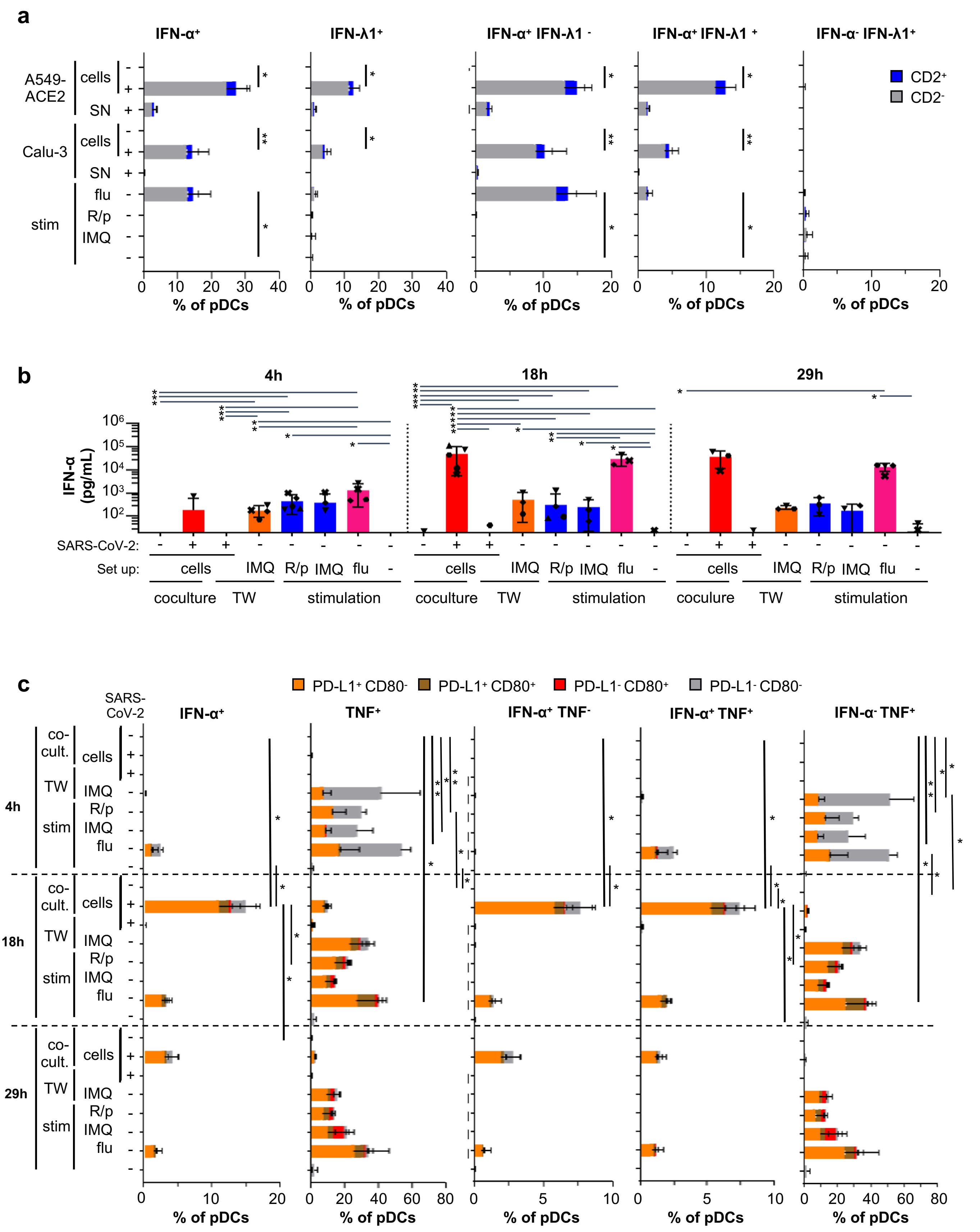

Supplementary Fig. 4

Supplementary Fig. 4. Cell-cell contact sensing of SARS-CoV-2-infected cells by pDCs induces a robust production of IFN-I/λ and other cytokines. Human pDCs isolated from healthy donors were cocultured with SARS-CoV-2-infected [+] or uninfected [-] cells, or were incubated with 100μl of cell-free SN collected immediately prior to coculture from the corresponding SARS-CoV-2-infected cells or were stimulated by cell-free influenza virus or synthetic agonists, as in **Fig. 4d-e**. **a,** Quantification by flow cytometry of the frequency of pDCs positive for IFN-α and/or IFN-λ1 in regard to positivity for CD2 subsets at 14-16 hours post-cocultures .**b-c.** Kinetic analysis of pDC cocultured with SARS-CoV-2- infected cells or not *versus* stimulations and determined at the coculture duration as indicated (top of the graph). **a,** quantification of IFN-α in SNs. **b,** Kinetic quantification by flow cytometry of the frequency of pDCs positives for IFN-α and/or TNF in regard to their diversification as PD-L1$^+$ and/or CD80$^+$ cells. Bars represent means ± SD; n=3-5 independent experiments using distinct healthy donors. The data were analyzed using Kruskal-Wallis Global test and p-values were calculated with Tukey and Kramer test; ns: p >0,05; *p<0,05; **p<0,005. Source data are provided as a Source Data file.

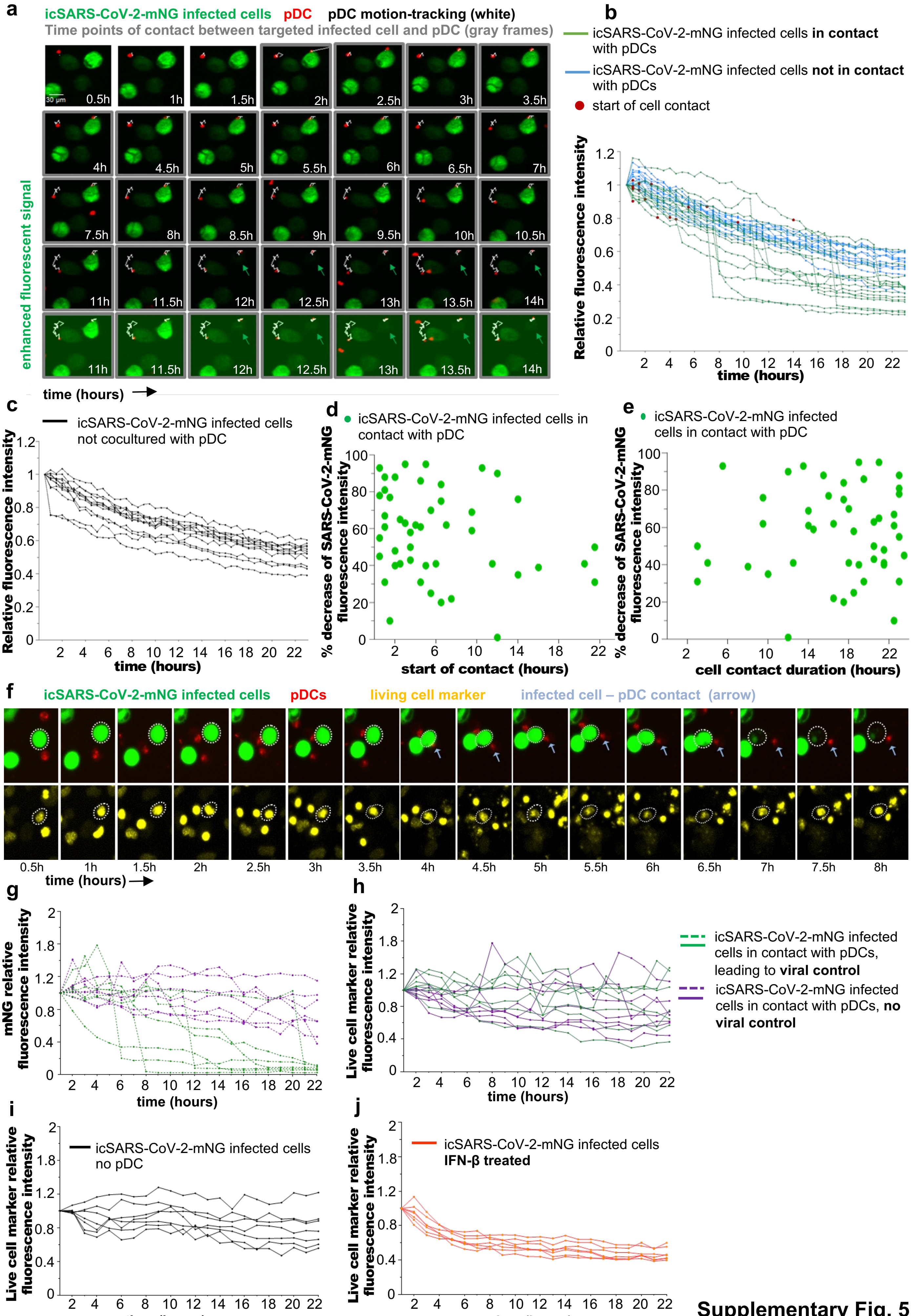

Supplementary Fig. 5

Supplementary Fig. 5. Targeted antiviral activity of pDCs toward SARS-CoV-2-infected cells. Live imaging of coculture of icSARS-CoV-2-mNG-infected cells with pDCs by spinning-disk confocal analysis, performed as in **Fig. 5. a,** Representative time-sequence of pDCs (red), tracked using motion automatic tracking plug-in in image J (white line) in contact with icSARS-CoV-2-mNG-infected cells (green arrow). The time points when pDCs are in contact with infected cells are framed in gray. Bottom panels show same imaging of the last 7 time points with enhanced fluorescence signal. **b-c**, Quantification of mNeongreen fluorescence intensity over time in individual icSARS-CoV-2-mNG-infected cells cocultured with pDCs when in contact (green curves) *versus* not in contact (blue curves) with pDCs (**b**), and as control/reference, in parallel cultures of icSARS-CoV-2-mNG-infected cells without pDC (**c**). The time point corresponding to the onset/start of contact is indicated by a red dot. Imaging analysis was performed and represented as in **Fig. 5b-c**; n=12 individual recorded cell analyzed per condition from one representative experiment. **d-e**, Scatter graphs representing the decrease of icSARS-CoV-2-mNG fluorescence intensity in individual icSARS-CoV-2-mNG infected cell identified as in contact with pDC and in relationship with the time of contact onset (**d**) and cell contact duration **(e)** expressed in hours. Results are expressed as the percentage of decrease of icSARS-CoV-2-mNG fluorescence at the end relative to the beginning of the contact (set to 100); 4 independent experiments with each dots representing an individual infected cell; n=48 (**d**) and n=48 (**e**). **f-j,** The icSARS-CoV-2-mNG-infected cells were stained with a fluorescent live cell marker prior to coculture with pDCs and real-time imaging by spinning-disk confocal analysis. **f,** Representative time-sequence of pDCs (DiD stained, in red) cocultured with icSARS-CoV-2-mNG-infected cells (green) stained by living cell marker (yellow, lower panels). Dotted white cercles indicate the infected cells with a robust decrease of mNG fluorescence intensity, but not of the living cell marker (yellow, lower panels) and arrow indicated the pDC in contact to this infected cell. **g-j,** Calculation of the fluorescence intensity of mNeongreen (**g**) and the fluorescent live cell marker (**h-j**) over time in individual icSARS-CoV-2-mNG-infected cells cocultured and in contact with pDCs, leading to control of viral replication defined as a decreased fluorescence intensity > 50% relative to the initial mNG fluorescent intensity (green curves) or not (purple curves). **i-j,** Simultaneous record of cultures of icSARS-CoV-2-mNG-infected cells in the absence of pDC (black, **i**) or treated with recombinant IFN-β (100 UI/mL, red, **j**) served as control/reference. The results are presented as the fluorescence intensity at the indicated time relative to time 0 of record corresponding to the contact onset and set to 1 (**g-h**); n= 7 individually recorded cells analyzed per condition from 2 independent experiments, and representative of a total n= 15 individually recorded cells analyzed per condition. Source data are provided as a Source Data file.

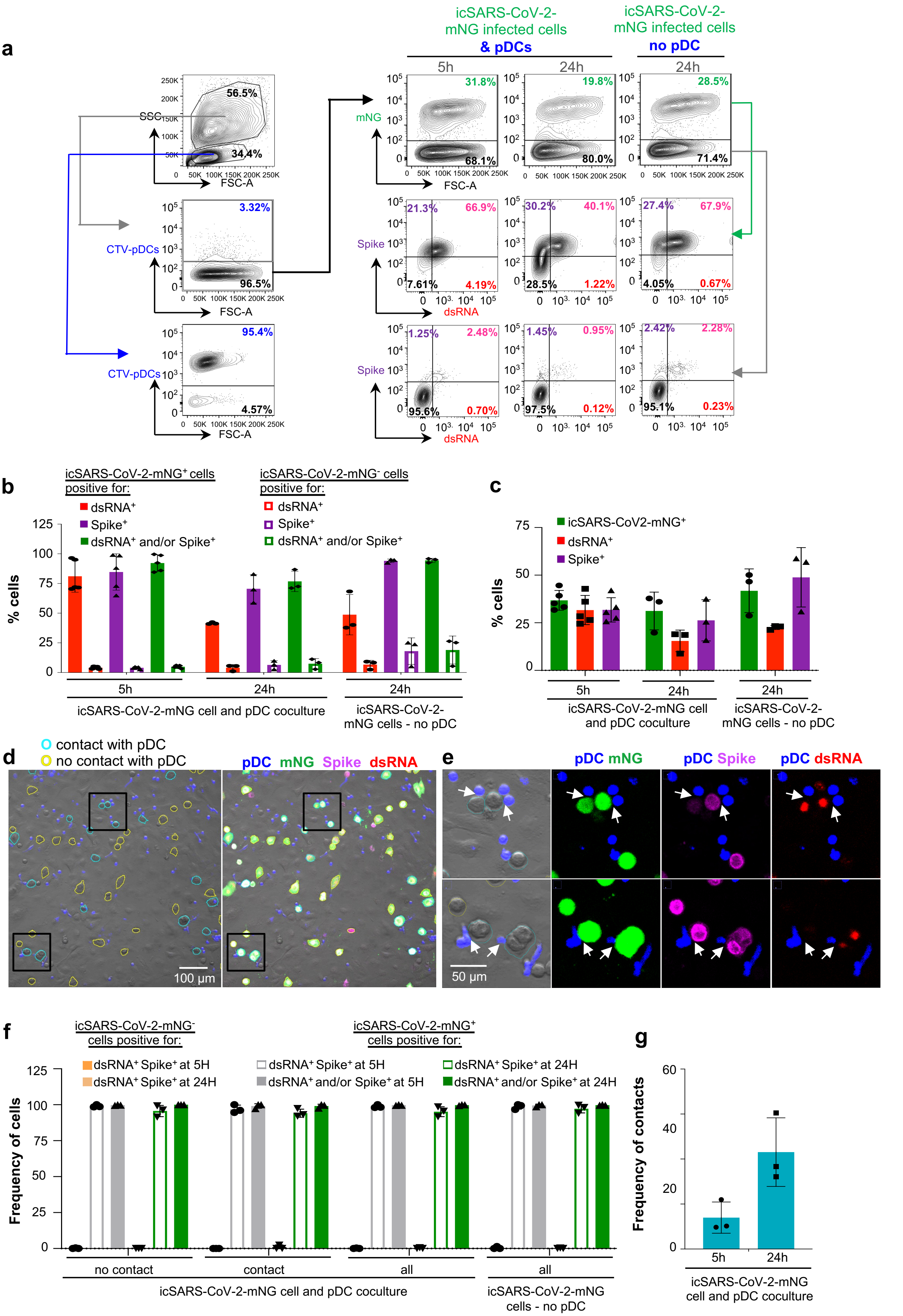

Supplementary Fig. 6

Supplementary Fig. 6. Validation of the icSARS-CoV-2-mNG to track in live imaging the viral spread and replication, related to Fig. 4f-g and Fig. 5, A549-ACE2 cells were infected by icSARS-CoV-2-mNG for 24 hours and then cocultured with isolated pDCs or not [no pDC], as in **Fig. 4f-g**, for 5 and 24 hours. The dsRNA viral replicative intermediate and the Spike protein — representative of viral replication/infection — were assessed by Flow cytometry in the cell population gated as pDC-CTV$^-$ (**a**, left panels representative dot blots of pDCs and infected cells cocultured for 5 hours), and further sorted according to mNG expression (**a**, 3 upper panel on the right side): to as mNG$^+$ (middle panels) *versus* mNG$^-$ cells (lower panel). **b**, The cell frequency of dsRNA$^+$, Spike$^+$ *versus* dsRNA$^+$ and/or Spike$^+$ were quantified among icSARS-CoV-2-mNG$^+$ cells (plain bars) and icSARS-CoV-2-mNG$^-$ cells (empty bars) at 5 and 24 hours post-coculture with pDCs or without [no pDC]; means ± SD; n=3 independent experiments. The corresponding representative dot blots are presented in **a,** middle and lower panel on the right side). **c,** As complementary controls, the cell frequency of icSARS-CoV-2-mNG$^+$, dsRNA$^+$, Spike$^+$ were quantified among cell population sorted as non-CTV-pDC$^-$ (without the distinction of mNG$^+$ and mNG$^-$). Error bars represent the means ± SD; each dot represents one independent experiments. **d-g,** Cocultures of icSARS-CoV-2-mNG-infected cells and pDCs were performed as in **Fig. 5**. **a-e,** Representative confocal imaging of pDCs (CTV-stained, blue) and icSARS-CoV-2-mNG-infected cells (mNG, green) immunostained by antibodies against dsRNA (red) and Spike (purple) at 24 hours post-cocultures. **d,** representative pictures of CTV-detection projected on the phase contrast imaging and presenting the automatic tracking of infected cells in contact with pDCs (*i.e.,* defined within a cell-to-cell distance < 5 μm; blue circles) or not in direct contact (yellow circles) and corresponding detections of CTV-pDCs, mNG, dsRNA and Spike protein projected on phase contrast imaging, left and right panels respectively. **e,** Magnifications of the two black-boxed areas, displayed from left to right panels for detection of: CTV/phase contrast with the tracking of contact/no contact with a pDC (blue/yellow circles); mNG/CTV; Spike/CTV and dsRNA/CTV. **f,** Quantification of the frequency of cells positive for both dsRNA$^+$ and Spike$^+$ (plain bars) or dsRNA$^+$ and/or Spike$^+$ (empty bars) among either the icSARS-CoV-2-mNG$^-$ cells (orange) or icSARS-CoV-2-mNG$^+$ cells at 5 hours (grey bars) and 24 hours (green bars) of coculture when defined in cells not in contact with pDC [no contact], nearby pDCs *i.e.,* cell-to-cell distance < 5 μm [contact] and in all cells cocutured with pDCs [all] or not [all/no pDC], as indicated on the X-axis. **g,** Quantification of the frequency icSARS-CoV-2-mNG$^+$ cells in contact with pDCs at 5 and 24 hours of coculture. Results represent means ± SD; n=3 independent experiments, including n=1495; n=1153; n=859 and n= 444 analyzed cells for infected cell/pDC cocultures at 24 hours; idem at 5 hours; infected cell/no pDC at 24 hours and idem at 5 hours, respectively. Source data are provided as a Source Data file.

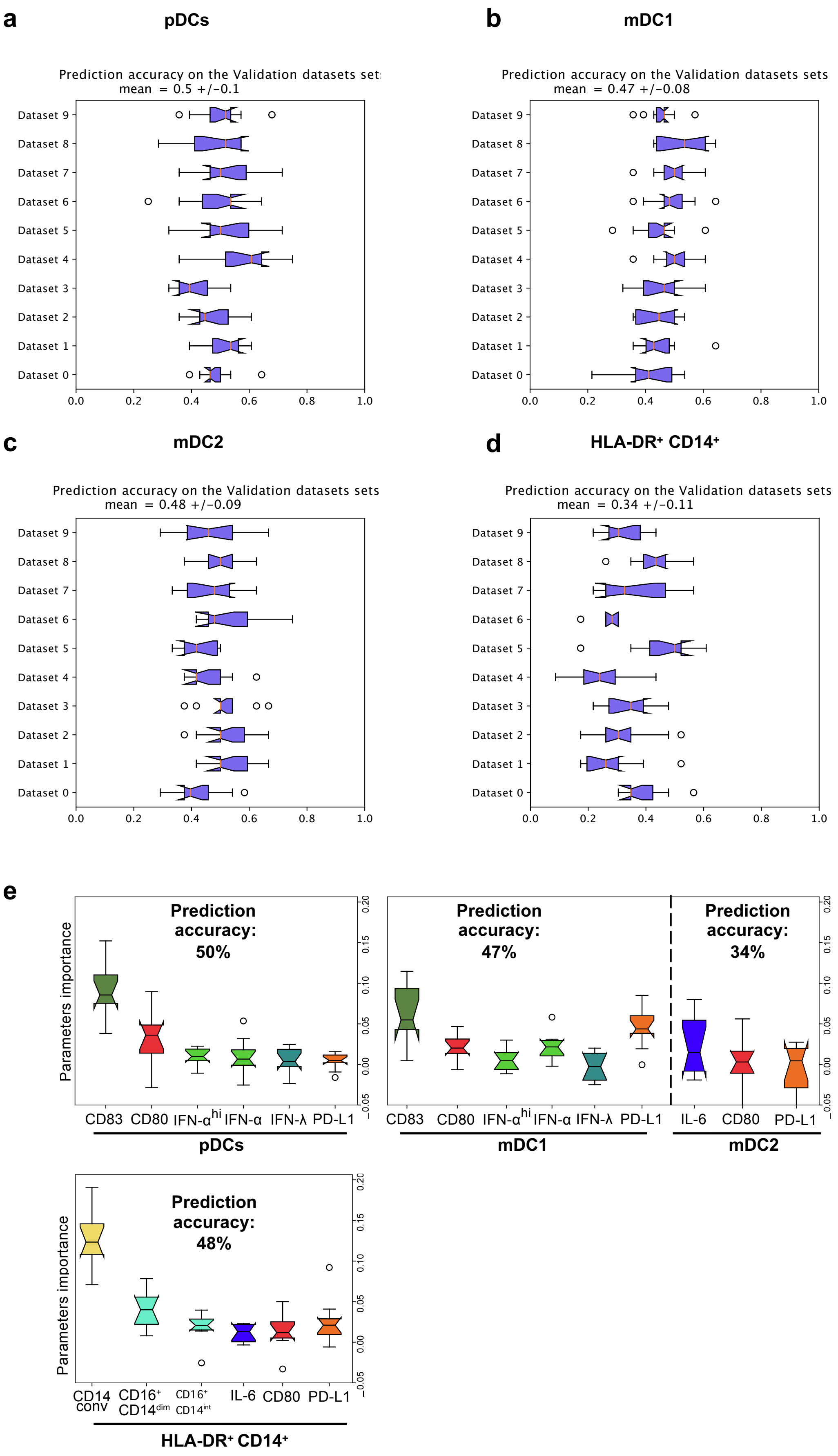

Supplementary Fig. 7

Supplementary Fig. 7. Analysis of the flow cytometry dataset using a Machine learning approach based on Gradient Boosting. PBMCs issued from the different groups of patients (*i.e.*, healthy donors; severe; mild/asymptomatic early; mild/asymptomatic late were cocultured for 14-16 hours with SARS-CoV-2-infected or uninfected A549-ACE2 cells, or treated with agonists [31.8 μM R848 and 42.22 μM polyI:C or 2.04 μM LPS], followed by the multiparametric analysis using flow cytometry. Analysis of the flow cytometry dataset was performed using a Machine learning approach based on Gradient Boosting. Graphs represent the mean accuracy prediction for the validation sets with the cell type specific models: pDCs (**a**), mDC1 (**b**), mDC2 (**c**) and HLA-DR$^+$ CD14$^+$ monocytes (**d**). For each cell type, 10 datasets were randomly generated. Each Validation set was used to challenge 10 models built with different down-sampling coupled to a cross-validation approach with 10 splits. The accuracy obtained with each cell type is indicated in the upper-title with the standard deviation taking into account all predictions. **e,** Upper-titles indicate the predictive accuracy in the validation set of samples for each cell populations as gated as pDCs, mDC1 and mDC2 subsets and HLA-DR+CD14$^+$ monocytes (see gating strategies in **Supplementary Fig. 2a**). Graphs display the parameters importance in predicting the severity/group of patients from the validation set, *via* comparative analyses of all cell surface expressed-differentiation markers and intracellular cytokines defined in different cell types, with inclusion of the distinction of IFN-α^{+all} and IFN-α^{hi}. Error bars correspond to the standard deviation of the mean importance of each parameter for each of the 10 down-sampling iterations over all iterations. For all graphs, bounds of the box plots correspond to the Interquartile Range (IQR) and the median displayed as a line in the box. Notches represent the confidence interval (CI) around the median. In case values of the CI are less than the lower quartile or greater than the upper quartile, the notches will extend beyond the box, giving it a distinctive « flipped » appearance. The lower whisker corresponds to [Q1-1.5*IQR (where Q1 corresponds to the first quartile)], while the upper whisker corresponds to [Q3+1.5*IQR (where Q3 corresponds to the third quartile)]. Beyond the whiskers, data are considered outliers and are plotted as individual points. Source data are provided as a Source Data file.

Fig. 1a

	tPBMCs & SN	tPBMCs & cont cells	tPBMCs & inf cells	PBMC/no pDC	iso pDCs & cont cells	iso pDCs & inf cells
tPBMCs & cont cells	NS					
tPBMCs & inf cells	1.3E-2	2.0E-2				
PBMC/no pDC	NS	NS	2.0E-2			
iso pDCs & cont cells	NS	NS	1.0E-2	NS		
iso pDCs & inf cells	1.0E-2	1.3E-2	1.0E-2	1.3E-2	1.0E-2	
no PBMC/pDC	NS	NS	2.7E-2	NS	NS	2.0E-2

Fig. 1b

	tPBMCs & SN	tPBMCs & cont cells	tPBMCs & inf cells	PBMC/no pDC	iso pDCs & cont cells	iso pDCs & inf cells
			p-value			
tPBMCs & cont cells	NS					
tPBMCs & inf cells	7.8E-3	3.0E-3				
PBMC/no pDC	NS	NS	7.8E-3			
iso pDCs & cont cells	NS	NS	2.2E-3	NS		
iso pDCs & inf cells	5.2E-3	2.2E-3	9.6E-4	6.1E-3	1.8E-3	
no PBMC/pDC	NS	NS	4.7E-3	NS	2.8E-3	NS

Fig. 3a

Conditions	p-values	Conditions	p-values	Conditions	p-values
cont/inf A549	0.1422	cont vs inf Calu	0.2336	flu vs R/p	0.7757
cont vs SN A549	0.6757	cont vs SN Calu	0.7814	flu vs IMQ	0.9817
cont A549 vs unst	0.5854	cont Calu vs unst	0.6416	flu vs -	0.08075
inf vs SN A549	0.8285	inf vs SN Calu	0.7814	flu vs unst	0.005452
inf A549 vs unst	0.003771	inf Calu vs unst	0.007665	R/p vs IMQ	0.9832
SN A549 vs unst	0.09953	SNCalu vs unst	0.1273	R/p vs -	0.495
				R/p vs unst	0.06238
				IMQ vs -	0.3019
				IMQ vs unst	0.03644
				- vs unst	0.7886

Fig. 3b

Conditions	p-values	Conditions	p-values	Conditions	p-values
cont vs inf A549	0.007987	cont vs inf Calu	NS	flu vs R/p	0.908
cont vs SN A549	0.3505	cont vs SN Calu	NS	flu vs IMQ	0.9025
inf vs SN A549	0.3505	inf vs SN Calu	NS	flu vs -	0.07767
				R/p vs IMQ	0.4755
				R/p vs -	0.1907
				IMQ vs -	0.008473

Fig. 3c

Conditions	p-values	Conditions	p-values	Conditions	p-values
**PD-L1$^+$ CD83$^-$**					
cont vs inf A549	0.1815	cont vs inf Calu	0.004845	flu vs R/p	0.2267
cont vs SN A549	0.009154	cont vs SN Calu	0.2592	flu vs IMQ	0.5517
inf vs SN A549	0.4671	inf vs SN Calu	0.2592	flu vs -	0.735
				R/p vs IMQ	0.9216
				R/p vs -	0.008514
				IMQ vs -	0.05544
**PD-L1$^+$ CD83$^+$**					
cont vs inf A549	0.009154	cont vs inf Calu	0.004845	flu vs R/p	0.2267
cont vs SN A549	0.1815	cont vs SN Calu	0.2592	flu vs IMQ	0.5517
inf vs SN A549	0.4671	inf vs SN Calu	0.2592	flu vs -	0.004232
				R/p vs IMQ	0.9216
				R/p vs -	0.3894
				IMQ vs -	0.1195
**PD-L1$^+$ CD83$^+$**					
cont vs inf A549	0.004845	cont vs inf Calu	NS	flu vs R/p	0.1114
cont vs SN A549	0.2592	cont vs SN Calu	NS	flu vs IMQ	0.9982
inf vs SN A549	0.2592	inf vs SN Calu	NS	flu vs -	0.2066
				R/p vs IMQ	0.04494
				R/p vs -	0.9891
				IMQ vs -	0.09969

Fig. 3d

Conditions	p-values	Conditions	p-values	Conditions	p-values
**PD-L1$^+$ CD80$^-$**					
cont vs inf A549	0.02835	cont vs inf Calu	0.01995	flu vs R/p	0.7729
cont vs SN A549	0.1364	cont vs SN Calu	0.3721	flu vs IMQ	0.3158
inf vs SN A549	0.8897	inf vs SN Calu	0.3721	flu vs -	0.006139
				R/p vs IMQ	0.7729
				R/p vs -	0.03943
				IMQ vs -	0.5359
**PD-L1$^+$ CD80$^+$**					
cont vs inf A549	0.08346	cont vs inf Calu	NS	flu vs R/p	0.824
cont vs SN A549	0.03957	cont vs SN Calu	NS	flu vs IMQ	0.932
inf vs SN A549	0.8897	inf vs SN Calu	NS	flu vs -	0.3102
				R/p vs IMQ	0.9975
				R/p vs -	0.01486
				IMQ vs -	0.07767
**PD-L1$^-$ CD80$^+$**					
cont vs inf A549	NS	cont vs inf Calu	NS	flu vs R/p	0.7172
cont vs SN A549	NS	cont vs SN Calu	NS	flu vs IMQ	0.2729
inf vs SN A549	NS	inf vs SN Calu	NS	flu vs -	0.6339
				R/p vs IMQ	0.7729
				R/p vs -	0.04723
				IMQ vs -	0.007457

Fig. 3e

Conditions	p-values	Conditions	p-values	Conditions	p-values
cont vs inf A549	0.03711	cont vs inf Calu	0.01428	flu vs R/p	0.929
cont vs SN A549	0.03064	cont vs SN Calu	0.09681	flu vs IMQ	0.9986
inf vs SN A549	0.9969	inf vs SN Calu	0.836	flu vs -	0.04494
				R/p vs IMQ	0.967
				R/p vs -	0.2707
				IMQ vs -	0.0679

Fig. 3f

Conditions with A549 cells	p-values	Conditions with Calu cells	p-values
no pDC cont vs no pDC inf A549	0.302	no pDC cont vs no pDC inf Calu	NS
no pDC cont vs pDC cont A549	0.7578	no pDC cont vs pDC cont Calu	NS
no pDC cont vs pDC inf A549	0.9338	no pDC cont vs pDC inf Calu	NS
no pDC inf vs pDC cont A549	0.8465	no pDC inf vs pDC cont Calu	NS
no pDC inf vs pDC inf A549	0.04242	no pDC inf vs pDC inf Calu	NS
pDC cont vs pDC inf A549	0.2883	pDC cont vs pDC inf Calu	NS

Supplementary Fig. 8

		A549-ACE-2 cells			Calu-3			Hu7.5.1			293-ACE2		
		no.pDC & cont cells	no.pDC & inf cells	pDCs & cont cells	no.pDC & cont cells	no.pDC & inf cells	pDCs & cont cells	no.pDC & cont cells	no.pDC & inf cells	pDCs & cont cells	no.pDC & cont cells	no.pDC & inf cells	pDCs & cont cells
MxA	no.pDC & inf cells	0.9912			0.09751			1			0.8536		
	pDCs & cont cells	0.1199	0.05911		0.9339	0.3194		0.4559	0.4559		0.3765	0.8536	
	pDCs & inf cells	0.0002978	8.249E-05	0.2551	0.01247	0.8798	0.06793	0.003389	0.003389	0.2035	2.88E-03	3.78E-02	0.2407
ISG15	no.pDC & inf cells	0.1986			0.1598			0.9831			0.9738		
	pDCs & cont cells	0.9999	0.1786		0.9339	0.4464		0.6881	0.8831		0.9821	0.9999	
	pDCs & inf cells	0.02854	1.248E-05	0.03322	0.005976	0.6343	0.03767	0.003389	0.0118	0.09194	0.07604	0.02386	0.02789
IFNL	no.pDC & inf cells	0.2307			0.186			0.8465			0.4464		
	pDCs & cont cells	0.8095	0.7479		1	0.186		0.9825	0.6343		0.6343	0.9909	
	pDCs & inf cells	0.00526	0.4924	0.07453	0.03057	0.8798	0.03057	0.05616	0.3194	0.01976	0.005976	0.2818	0.1598
IL6	no.pDC & inf cells	0.7103			0.247			0.637			0.7084		
	pDCs & cont cells	0.829	0.9966		0.9988	0.3194		0.7374	0.9985		0.9186	0.3181	
	pDCs & inf cells	0.0002978	0.01506	0.00756	0.03057	0.8095	0.04614	0.001363	0.0614	0.03978	0.1765	0.01043	0.5058
TNF	no.pDC & inf cells	0.02443			0.3543			0.4559			0.1862		
	pDCs & cont cells	1	0.02443		1	0.3543		1	0.4559		1	0.1862	
	pDCs & inf cells	0.0003726	0.6442	0.0003726	0.03136	0.6923	0.03136	0.003389	0.2035	0.003389	0.004638	0.5397	0.004638

Fig. 4a

Fig. 4d IFN-α^{+} TNF^{-}

Conditions	p-values	Conditions	p-values	Conditions	p-values
cont vs inf A549	0.007987	cont vs inf Calu	0.01995	flu vs R/p	0.4755
cont vs SN A549	0.3505	cont vs SN Calu	0.3721	flu vs IMQ	0.05547
inf vs SN A549	0.3505	inf vs SN Calu	0.3721	flu vs -	0.02287
				R/p vs IMQ	0.5055
				R/p vs -	0.3631
				IMQ vs -	1

Fig. 4e IFN-α^{+} IL-29^{-}

Conditions	p-values	Conditions	p-values	Conditions	p-values
cont vs inf A549	0.01995	cont vs inf Calu	0.004845	flu vs R/p	0.2765
cont vs SN A549	0.3721	cont vs SN Calu	0.2592	flu vs IMQ	0.197
inf vs SN A549	0.3721	inf vs SN Calu	0.2592	flu vs -	0.02494
				R/p vs IMQ	0.9973
				R/p vs -	0.6853
				IMQ vs -	0.8

Fig. 4d IFN-α^{+} TNF^{+}

Conditions	p-values	Conditions	p-values	Conditions	p-values
cont vs inf A549	0.007987	cont vs inf Calu	0.01995	flu vs R/p	0.3725
cont vs SN A549	0.3505	cont vs SN Calu	0.3721	flu vs IMQ	0.06909
inf vs SN A549	0.3505	inf vs SN Calu	0.3721	flu vs -	0.03193
				R/p vs IMQ	0.6761
				R/p vs -	0.566
				IMQ vs -	1

Fig. 4e IFN-α^{+} IL-29^{+}

Conditions	p-values	Conditions	p-values	Conditions	p-values
cont vs inf A549	0.01995	cont vs inf Calu	0.004845	flu vs R/p	0.197
cont vs SN A549	0.3721	cont vs SN Calu	0.2592	flu vs IMQ	0.2592
inf vs SN A549	0.3721	inf vs SN Calu	0.2592	flu vs -	0.02775
				R/p vs IMQ	0.9986
				R/p vs -	0.8206
				IMQ vs -	0.7332

Fig. 4d IFN-α^{-} TNF^{+}

Conditions	p-values	Conditions	p-values	Conditions	p-values
cont vs inf A549	0.01116	cont vs inf Calu	NS	flu vs R/p	0.4755
cont vs SN A549	0.2846	cont vs SN Calu	NS	flu vs IMQ	0.09454
inf vs SN A549	0.4824	inf vs SN Calu	NS	flu vs -	0.01476
				R/p vs IMQ	0.6582
				R/p vs -	0.2765
				IMQ vs -	0.9779

Fig. 4e IFN-α^{-} IL-29^{+}

Conditions	p-values	Conditions	p-values	Conditions	p-values
cont vs inf A549	NS	cont vs inf Calu	NS	flu vs R/p	NS
cont vs SN A549	NS	cont vs SN Calu	NS	flu vs IMQ	NS
inf vs SN A549	NS	inf vs SN Calu	NS	flu vs -	NS
				R/p vs IMQ	NS
				R/p vs -	NS
				IMQ vs -	NS

Supplementary Fig. 3d CD2^{hi} CD5^{+} Axl^{+}

Conditions	p-values	Conditions	p-values	Conditions	p-values
cont vs inf A549	0.03784	cont vs inf Calu	0.09847	flu vs piC	NS
cont vs SN A549	0.06228	cont vs SN Calu	0.02209	flu vs IMQ	NS
inf vs SN A549	0.979	inf vs SN Calu	0.8264	flu vs medium	NS
				piC vs IMQ	NS
				piC vs medium	NS
				IMQ vs medium	NS

Supplementary Fig. 3d CD2^{hi} CD5^{-} Axl^{-}

Conditions	p-values	Conditions	p-values	Conditions	p-values
cont vs inf A549	0.01663	cont vs inf Calu	NS	flu vs piC	NS
cont vs SN A549	0.122	cont vs SN Calu	NS	flu vs IMQ	NS
inf vs SN A549	0.7126	inf vs SN Calu	NS	flu vs medium	NS
				piC vs IMQ	NS
				piC vs medium	NS
				IMQ vs medium	NS

Supplementary Fig. 8

Supplementary Fig. 8. Exact p-values obtained from the statistical analyses. All exact p-value are presented for each individual panel of Fig. 1, 3 and 4; Supplementary Fig. 3.

Supplementary Table 1. Viral load and cytokinic profiles of SARS-CoV-2 infected patients with mild/asymptomatic and severe/critically-ill COVID-19. Viral load in nasal swab determined by qPCR relative to the volume and number of cells. Abbreviations: ND ; not detected, / ; not determined.

group: mild/asymptomatic patients										
ID	day post symptom	Viral load				Cytokinic level in the blood of patients				
		Ct SARS N	log10 cp/ml	log10 copies /10^3 cell	Results	IFN-α (fg/mL)	IFN score	IFN-λ1 (pg/mL)	IL-6 (pg/mL)	IFN-γ (pg/mL)
P41-01	4	18.81	7.11	5.1	Positive	5919.0	149.96	38.26	/	41.17
P41-02	11	27.56	4.62	2.7	Positive	2267.0	71.71	15.64	/	149.17
P41-03	18	34.16	2.45	0.6	Positive	6.6	2.61	7.76	0.48	13.87
P41-04	25	37.23	0.00	ND	Positive	/	1.56	2.90	0.27	9.98
G38-01	1	23.37	5.72	3.4	Positive	7382.0	26.39	40.91	/	31.55
G38-02	8	23.22	6.00	3.9	Positive	53.3	2.8	6.24	/	11.65
G38-03	15	29.95	3.96	1.6	Positive	0.0	2.35	8.82	1.56	9.85
G38-04	22	23.3	5.73	3.8	Positive	/	ND	0.85	0.90	4.84
G17-01	4	21.65	6.24	3.9	Positive	245.0	39.02	7.60	/	151.88
G17-02	10	/	/	/	/	9.2	4.46	9.23	/	15.36
G17-03	19	/	/	/	/	0.0	3.36	3.37	0.23	94.97
G17-04	25	/	/	/	/	/	1.86	0.85	0.90	4.84
G17-05	183	/	/	/	/	/	/	2.19	0.71	13.85
A30-01	1	23.53	5.67	4.2	Positive	3683.0	123.66	8.83	1.06	13.78
A30-02	8	26.59	4.79	2.8	Positive	173.2	27.65	4.32	0.60	13.46
A30-03	14	ND	1.65	-0.5	Positive low	0.0	1.41	2.16	0.33	5.61
A30-04	22	ND	ND	ND	Negative	ND	1.68	2.67	0.76	6.45
A30-05	183	/	/	/	/	/	/	1.84	0.24	3.47
L175-01	1	16.73	7.6	6.8	Positif	2.9	173.9	24.17	0.64	108.69
L175-02	8	/	/	/	/	25.2	18.27	3.58	0.47	18.12
L175-03	13	38.55	0.87	ND	Positive low	/	3.71	2.14	0.32	8.75
L175-04	20	/	/	/	/	/	2.74	2.01	0.27	9.73
L175-05	183	/	/	/	/	/	/	1.89	0.32	7.71
P29-01	2	22.73	5.91	3.9	Positive	774.0	76.76	9.04	/	203.40
P29-02	9	/	/	/	/	4.2	2.63	3.79	/	9.27
P29-03	16	/	/	/	/	1.4	2.3	1.97	0.39	13.34
P29-04	23	/	/	/	/	/	2.07	/	/	/
P29-05	30	/	/	/	/	/	/	2.32	0.27	7.88
P29-06	183	/	/	/	/	/	/	2.72	0.30	11.62

group: severe patients										
ID	day post symptom	Viral load				Cytokinic level in the blood of patients				
		Ct SARS N	log10 cp/ml	log10 copies /10^3 cell	Results	IFN-α (fg/mL)	IFN score	IFN-λ1 (pg/mL)	IL-6 (pg/mL)	IFN-γ (pg/mL)
C-01	27	30.8	/	/	Positive	22.4	0.9	3.69	11.09	ND
C-02	37	ND	/	/	Negative	13.3	< 0.4	1.34	0.86	4.59
B-01	9	/	/	/	Positive	0.0	2.7	21.99	/	48.12
B-02	11	/	/	/	Positive	0.0	0.6	14.29	/	11.37
B-03	15	/	/	/	Positive	0.0	0.8	12.76	/	5.07
H-01	15	25.5	/	/	Positive	0.0	0.5	9.00	107.49	15.27
H-02	18	pos	/	/	Positive	28.0	1	7.83	138.92	8.82
H-03	20	pos	/	/	Positive	16.7	0.9	6.02	260.51	79.26
F-01	12	35.7	/	/	Positive	0.8	2.37	/	/	/
F-02	28	ND	/	/	Negative	5.6	0.53	/	/	/

F-03	35	ND	/	/	Negative	0	0.39	/	/	/
O-01	8	19.7	/	/	Positive	1025	39.89	/	/	/
O-02	16	21.1	/	/	Positive	0	1.19	2.46	1.63	8.68
O-03	23	32.7	/	/	Positive	0	1.53	7.90	6.31	39.75
O-04	30	ND	/	/	Negatif	3.9	0.87	11.05	9.18	50.80
R-01	36	32.1	/	/	Positive	7.5	1.1	/	/	/
R-02	43	ND	/	/	Negative	7.3	0.74	3.02	18.83	7.36
R-03	50	ND	/	/	Negative	11	2.28	5.04	2.44	16.63

Supplementary Table 2. Primer sequences for quantitative real-time PCR

Gene	Forward Primer	Reverse Primer
MXA	ACAGGACCATCGGAATCTTG	CCCTTCTTCAGGTGGAACAC
ISG15	GACAAATGCGACGAACCTCT	CGGCCCTTGTTATTCCTCA
IFNλ1	TCCTAGACCAGCCCCTTCA	GTGGGCTGAGGCTGGATA
IL6	GTCAGGGGTGGTTATTGCAC	AGTGAGGAACAAGCCAGAGC
TNF	AGATGATCTGACTGCCTGGG	CTGCTGCACTTTGGAGTGAT
GAPDH	AGGTGAAGGTCGGAGTCAACG	TGGAAGATGGTGATGGGATTTC

Supplementary Table 3: Regulation of the selected biomarkers by the IRF/IFN-I *versus* NF-ϰB pathways. The identification of cis-acting regulatory elements in promotor of the set of analyzed markers was performed as described in the Method section, using the FIMO and AME tools available on https://meme-suite.org/meme/[98,99]. Additional searches were performed using website, as reference: https://www.bu.edu/nf-kb/gene-resources/target-genes/ and http://www.interferome.org/interferome/home.jspx and the supplemental source/retrieved publications are indicated. The color-code corresponds to the number of cis-acting regulatory elements in each promotor. Abbreviations: ND; not determined. Of note, IRF7 and VAF transcription factors binds to *IFNα* and *IFNβ* promoter regions and induce the IFNα and IFNβ mRNA.

		consensus sequence for regulators related to:									
		IRF/IFN-I							NF-κB		
**name in the text**	**Accession number**	**IRF 7**	**IRF 3**	**IRF 1**	**IRF 5**	**STAT1: STAT2**	**IRF 9**	**IRF 8**		**https://www.bu.edu/nf-kb/gene-resources/target-genes/ and http://www.interferome.org/interferome/home.jspx**	**references**
IFNα1	NM_024013.3	2	2	6	0	0	0	0	0	N.D for NF-κB	
IFNα2	NM_000605.4	0	0	1	0	0	0	0	0	N.D for NF-κB	100
IFNα4	NM_021068.3	0	0	3	0	0	0	0	0	N.D for NF-κB	100
IFNβ1	NM_002176.4	1	2	2	1	1	0	0	0	*not directly demonstrated - likely for B lymphocytes and fibroblasts	101,102
ISG15	NM_005101.4	1	5	6	2	4	4	2	1	N.D for NF-κB	
*MxA**	NM_001144925.2	1	3	4	4	7	2	1	8	*kB site in the promoter but has not been shown to be controlled by NF-kB or the gene expression is associated with increased NF-kB activity	103
IFNλ1 IL28A	NM_172140.2	1	2	2	0	1	2	0	0	N.D for NF-κB	
IFNλ2 IL28B	NM_172138.2	1	1	4	1	1	0	0	1	N.D for NF-κB	
IFNλ3 IL29	NM_001346937.2	1	2	2	1	2	0	0	1	N.D for NF-κB	
CD70	NM_001252.5	0	0	1	0	0	0	0	2	NF-kB site: NFKAPPAB & NFKAPPAB50	
CD83	NM_001040280.3	0	0	0	0	0	0	0	4	Target Genes of NF-kB	45,104
TRAIL	NM_003810.4	0	2	4	0	2	0	0	1	Target Genes of NF-kB	105,106
TNF	NM_000594.4	0	2	1	1	1	1	1	2	Target Genes of NF-kB	107,108
HLA-DRa	NM_019111.5	0	0	1	0	0	1	1	0	N.D for NF-κB	
IL6	NM_001371096.1	1	0	3	1	0	1	0	1	Target Genes of NF-kB	109-111
CD80	NM_005191.4	0	0	2	0	0	0	0	0	Target Genes of NF-kB	112,113
PD-L1 (CD274 B7-H1)	NM_014143.4	3	2	2	1	3	2	1	0	Target Genes of NF-kB	46,114

Supplementary Table 4. List of reagents and biological tools used for the methods. This includes antibodies, dyes and staining solutions; virus/strains; chemicals, peptides, recombinant proteins; critical commercial assays; cell lines; and recombinant DNA.

REAGENT or RESOURCE	SOURCE	IDENTIFIER	CONCENTRATION or DILUTION (when concentration unknown)	Application	Validation
Antibodies					
mouse anti-human CD2 Bv785-conjugated (clone RPA-2.10)	BioLegend	Cat# 300234, RRID:AB_2800717	2.5 µg/mL	flow cytometry	manufacturer's information
mouse anti-human CD5 A700-conjugated (clone UCHT2)	BioLegend	Cat# 300632, RRID:AB_2632671	1.25 µg/mL		
mouse anti-human CD70 PE/Dazzle594-conjugated (clone 113-16)	BioLegend	Cat# 355123, RRID:AB_2820005	3.33 µg/mL		
mouse anti-human CD80 APC-H7-conjugated (clone L307.4)	BD Biosciences	Cat# 561134, RRID:AB_10565974	10-fold dilution		
mouse anti-human CD83 PE/Dazzle594-conjugated (clone HB15e)	BioLegend	Cat# 305328, RRID:AB_2564260	10 µg/mL		
mouse anti-human CD123 Bv711-conjugated (clone 6H6)	BioLegend	Cat# 306030, RRID:AB_2566354	5 µg/mL		
mouse anti-humain CD274/PE-Cy7-conjugated (clone MIH1)	BD Biosciences	Cat# 558017, RRID:AB_396986	10 µg/mL		
mouse anti-human CD303 Bv421-conjugated (clone 201A)	BioLegend	Cat# 354212, RRID:AB_2563871	10 µg/mL		
mouse anti- human HLA-DR Bv510-conjugated (clone L243)	BioLegend	Cat# 307646, RRID:AB_2561948	1.2 µg/mL		
mouse anti-human TNF PE-conjugated (clone MAb11)	BioLegend	Cat# 502909, RRID:AB_315261	10 µg/mL		
mouse anti-human TRAIL PE-conjugated (clone RIK-2)	BioLegend	Cat# 308205, RRID:AB_345291	10 µg/mL		
mouse anti-human IFN-a APC-conjugated (clone LT27:295)	Miltenyi Biotec	Cat# 130-092-602, RRID:AB_871558	10-fold dilution		
mouse anti-human IL-29 (IFN-λ1) purified (Clone 247801)	R & D Systems	Cat# MAB15981, RRID:AB_2125340	200 µg/mL		
mouse anti-human AXL A488-conjugated (clone 108724R)	R & D Systems	Cat# FAB154RG	10 µg/mL		
mouse anti-human CD123 PE or APC-conjugated (clone AC145)	Miltenyi	Cat# 130-090-901, RRID:AB_244209	20-fold dilution		
mouse anti-human BDCA-2 APC-conjugated (clone AC144)	Miltenyi	Cat# 130-090-905, RRID:AB_244165	20-fold dilution		
mouse anti-human CD11c BV605-conjugated (clone B-ly6)	BD Horizon	Cat# 563929, RRID:AB_2744276	50-fold dilution		
mouse anti-human CD56/NCAM FITC-conjugated (clone TULY56)	eBiscience	Cat# 11-0566-42, RRID:AB_2572459	5 µg/mL		
mouse anti-human HLA-DR BV711-conjugated (clone L243)	BioLegend	Cat# 307644, RRID:AB_2562913	1 µg/mL		
mouse anti-human IL-6 PE-conjugated (clone MQ2-13A5)	BioLegend	Cat# 501107, RRID:AB_315155	2,5 µg/mL		
mouse anti-human CD14 BV785-conjugated (clone M5E2)	BioLegend	Cat# 301840, RRID:AB_2563425	5 µg/mL		
mouse anti-human CD14 FITC-conjugated (clone M5E2)	BD Pharmingen	Cat# 555397, RRID:AB_395798	20-fold dilution		
mouse anti-human CD141 PerCP/Cy5.5-conjugated (clone M80)	Biolegend	Cat# 344112, RRID:AB_2561625	20 µg/mL		
mouse anti-human CD16 FITC-conjugated (clone B73.1)	BioLegend	Cat# 360716, RRID:AB_2563071	20 µg/mL		
mouse anti-human CD16 PacificBlue-conjugated (clone 3G8)	Biolegend	Cat# 302032, RRID:AB_2104003	10 µg/mL		
mouse anti-human CD19 FITC-conjugated (clone HIB19)	eBiscience	Cat# 11-0199-42, RRID:AB_10669461	10 µg/mL		
mouse anti-human CD1c PerCP-eFluor710-conjugated (clone L161)	eBiscience	Cat# 46-0015-42, RRID:AB_10548936	1.2 µg/mL		
mouse anti-human CD20 FITC-conjugated (clone 2H7)	BD Pharmingen	Cat# 555622, RRID:AB_395988	20-fold dilution		
mouse anti-human CD3 FITC-conjugated (clone UCHT1)	eBiscience	Cat# 11-0038-42, RRID:AB_2043831	10 µg/mL		
mouse anti-dsRNA unconjugated (clone J2 IgG2a)	SCICONS	Cat# 10010200, RRID:AB_2651015	10 µg/mL	flow cytometry & imaging	validated by serial dilution - uninfected cells as neg control
rabbit anti-SARS-CoV-2 Spike S2 unconjugated (polyclonal)	Sino Biological	40590-T62-100	200-fold dilution		
mouse anti-human integrin αL subunit unconjugated (clone 38)	antibodies online	Cat# ABIN375486, RRID:AB_10782956	10 µg/mL	coculture	validated by serial dilution, no pDC as neg control
mouse anti-human ICAM-1 unconjugated (clone LB-2)	BD Pharmingen	Cat# 559047, RRID:AB_397183	10 µg/mL		
goat anti-Rabbit IgG (H+L) AlexaFluor 546-conjugated	Invitrogen	Cat# A-11035, RRID:AB_143051	2 µg/mL	flow cytometry & imaging	manufacturer's information
goat anti-Rabbit IgG (H+L) AlexaFluor 647-conjugated	Invitrogen	Cat# A-31573, RRID:AB_2536183	2 µg/mL		
goat anti-mouse IgG (H+L) AlexaFluor 555-conjugated	Invitrogen	Cat# A32727, RRID:AB_2536164	2 µg/mL		
Dyes and staining solutions					
human TruStain FcX (Fc receptor blocking solution)	Biolegend	Cat# 422302, RRID:AB_2818986	N.A.	flow cytometry	manufacturer's information
BD Cytofix/Cytoperm Plus Kit (with BD GolgiPlug)	BD Biosciences	Cat# 555028	N.A.		
ZombieAqua fixable viability kit	Biolegend	Cat# 423101	1000-fold dilution		
ZombieGreen fixable viability kit	Biolegend	Cat# 423111	1000-fold dilution		
Fixable viability dye eFluor450	eBioscience	Cat# 65-0863-14	1000-fold dilution		
FITC Annexin V Apoptosis Detection Kit with 7-AAD	BioLegend	Cat#640922	N.A.		

Mix-n-Stain CF568 Dye Antibody Labeling Kit	Biotium	Cat# 92235	N.A.		
LIVE/DEAD Fixable Near-IR Dead Cell Stain Kit	Life Technologies	Cat# L10119	1000-fold dilution	Live imaging	manufacturer's information
vybrant cell-labeling solution (CM-DiI)	Life Technologies	Cat# V22888, CAS:180854-97-1	1000-fold dilution		
celltrace Violet cell Proliferation kit (CTV)	Life Technologies	Cat# C34557	1000-fold dilution		
Virus / strains					
SARS-CoV-2 clinical isolate	GISAID EpiCoVTM database: BetaCoV/France/IDF0571/2020; accession ID: EPI_ISL_411218	N/A	N.A.	flow cytometry, imaging, coculture	
icSARS-CoV-2-mNG (Wuhan)	kindly provided by Dr Pei-Yong Shi	N/A	N.A.		
Influenza A Virus (Flu A/H1N1/New caledonia)	kindly provided by Dr V. Lotteau (CIRI, Lyon, France	N/A	N.A.		
Chemicals, Peptides, and Recombinant Proteins					
Arp2/3 complex inhibitor I (CK-666)	Merck Millipore	Cat# 182515, CAS:442633-00-3	see in Figure Legend	coculture	validated by serial dilution
Imiquimod	Invivogen	Cat# tlrl-imqs, CAS:99011-78-6	20 µM		
polyICLMW	Invivogen	Cat# tlrl-picw, CAS : 31852-29-6	42.22 µM		
R848	Invivogen	Cat# vac-r848, CAS : 144875-48-9	31.8 µM		
IRS661 (5'-TGCTTGCAAGCTTGCAAGCA-3')	N.A.	synthesized on phosphorothioate backbone	see in Figure Legend		
poly-L-lysin (P6282)	Sigma-Aldrich	Cat# P6282, CAS:25988-63-0	N.A.	Live imaging	N.A.
Dulbecco's modified Eagle medium / Nutrient Mixture F-12 Ham (DMEM/F-12 1:1)	Life Technologies	Cat# 31331028	N.A.	coculture	manufacturer's information
RPMI 1640 Medium	Life Technologies	Cat# 31870025	N.A.		
Dulbecco's modified Eagle medium (DMEM)	Life Technologies	Cat# 41966029	N.A.		
Penicillin-Streptomycin	Life Technologies	Cat# 15140122	100 U/ml		
L-Glutamine	Life Technologies	Cat# 25030024	2 mM		
Non-Essential Amino Acids Solution	Life Technologies	Cat# 11140035	N.A.		
Sodium Pyruvate	Life Technologies	Cat# 11360039	1 mM		
Hepes	Life Technologies	Cat# 15630056	10 mM		
Trypsin-EDTA (0.05%), phenol red	Life Technologies	Cat# 25300054	N.A.	N.A.	
EDTA	Life Technologies	Cat# 15575038	0.48 mM	N.A.	
DMSO	Sigma-Aldrich	Cat# D2438, CAS: 67-68-5	10% final volume	N.A.	
Critical Commercial Assays					
BDCA-4-magnetic beads	Miltenyi	Cat# 130-090-532	N.A.	N.A.	manufacturer's information
VeriKine Human Interferon Alpha Multi-Subtype ELISA Kit	PBL Interferon Source	Cat# 41105-2	N.A.	N.A.	
IFN-lambda ELISA	PBL Interferon Source	Cat# 61840-1	N.A.	N.A.	
VeriKine Human Interferon Beta ELISA Kit	PBL Interferon Source	Cat# 41410-1	N.A.	N.A.	
High-Capacity cDNA Reverse Transcription Kit	Life Technologies	Cat# 4368813	N.A.	N.A.	N.A.
PowerUp SYBR Green Master Mix	Life Technologies	Cat# A25742	N.A.	N.A.	N.A.
Simoa IFN-α Reagent Kit	Quanterix	Cat# 100860	N.A.	N.A.	N.A.
Cell lines					
A549 cell line	ATCC	CCL-185	N.A.		
Calu-3 cell line	ATCC	HTB-55	N.A.		
NCI-H358 cell line	ATCC	CRL-5807	N.A.		
293T cell line	ATCC	CRL-3216	N.A.		
Human hepatoma cell line	Nakabayashi et al., 1982	Huh-7.5.1 (RRID:CVCL_E049)	N.A.		
Vero cell line (clone Vero E6)	Dr Bouloy; Institut Pasteur, France	CRL-1586, ATCC	N.A.		
Recombinant DNA					
lentivirus-based vector expressing ACE2	Dr C. Goujon, IRIM, France	https://www.addgene.org/145839/	RRL.sin.cPPT.SFFV/Ace2.IRES-puromycin.WPRE		
