# Supplementary material for: Severe COVID-19 patients have impaired plasmacytoid dendritic cell-mediated control of SARS-CoV-2
Source: Nat Commun. 2023 Feb 8;14:694. doi: 10.1038/s41467-023-36140-9 (PMC9907212; doi:10.1038/s41467-023-36140-9)
Supplement: Supplementary file 3 — Reporting Summary [file 41467_2023_36140_MOESM3_ESM.pdf]

nature portfolio

Corresponding author(s): DREUX Marlene

Last updated by author(s): 12/02/2022

# Reporting Summary

Nature Portfolio wishes to improve the reproducibility of the work that we publish. This form provides structure for consistency and transparency in reporting. For further information on Nature Portfolio policies, see our Editorial Policies and the Editorial Policy Checklist.

## Statistics

For all statistical analyses, confirm that the following items are present in the figure legend, table legend, main text, or Methods section.

| n/a | Confirmed | |
|---|---|---|
| ☐ | ☒ | The exact sample size ($n$) for each experimental group/condition, given as a discrete number and unit of measurement |
| ☐ | ☒ | A statement on whether measurements were taken from distinct samples or whether the same sample was measured repeatedly |
| ☐ | ☒ | The statistical test(s) used AND whether they are one- or two-sided<br>*Only common tests should be described solely by name; describe more complex techniques in the Methods section.* |
| ☐ | ☒ | A description of all covariates tested |
| ☐ | ☒ | A description of any assumptions or corrections, such as tests of normality and adjustment for multiple comparisons |
| ☐ | ☒ | A full description of the statistical parameters including central tendency (e.g. means) or other basic estimates (e.g. regression coefficient) AND variation (e.g. standard deviation) or associated estimates of uncertainty (e.g. confidence intervals) |
| ☐ | ☒ | For null hypothesis testing, the test statistic (e.g. $F$, $t$, $r$) with confidence intervals, effect sizes, degrees of freedom and $P$ value noted<br>*Give P values as exact values whenever suitable.* |
| ☒ | ☐ | For Bayesian analysis, information on the choice of priors and Markov chain Monte Carlo settings |
| ☐ | ☒ | For hierarchical and complex designs, identification of the appropriate level for tests and full reporting of outcomes |
| ☒ | ☐ | Estimates of effect sizes (e.g. Cohen's $d$, Pearson's $r$), indicating how they were calculated |

*Our web collection on statistics for biologists contains articles on many of the points above.*

## Software and code

Policy information about availability of computer code

| | |
|---|---|
| Data collection | Clinical data were recorded by a trained Clinical Research Associate using Clinsight software (version _ Csonline 7.5.720.1).<br>Flow Cytometry data were collected with BD FACSDIVA v8.01. |
| Data analysis | Statistical analysis were performed using GraphPad PRISM software (version 8.4.3)<br>and R Statistical Computing software (version 3.3.2)<br>Flow Cytometry data were analyzed with FlowJo software (version 10.8.1) and analytes measurement using Discovery workbench software (version 4.0, MSD) and nSolver software (version 4.0).<br>Gradient Boosting machine learning analysis, the following software and packages were used:<br>1/ Python 3.8.10 [GCC 9.4.0] on linux with the following packages indicated as package name [Version]:<br>joblib [1.1.0]; matplotlib [3.5.1]; numpy [1.22.2]; pandas [1.4.0]; scikit-learn [1.0.2]<br>2/ R (version 4.2.1): RStudio 2021.09.1+372; "Ghost Orchid" Release (8b9ced188245155642d024aa3630363df 611088a, 2021-11-08) for Ubuntu Bionic; Mozilla/5.0 (X11; Linux x86_64) AppleWebKit/537.36 (KHTML, like Gecko) QtWebEngine/5.12.8 Chrome/69.0.3497.128 Safari/537.36 ; and with the following packages indicated as package name [version]: caret [6.0.90]; ggplot2 [3.3.5]; knitr [1.37]; nnet [7.3.17]; reticulate [1.25]; tidyverse [1.3.1]; tinytex [0.36].<br>PCA analyses were performed using the R princomp function, R Software (version 4.1.0) with the correlation matrix approach.<br>Confocal mircroscopy analysis using Image J software (version 1.53k Java 1.8.0-_172)<br>RT-qPCR analysis using StepOnePlus Real-Time PCR sofware (version 2.3 ). |

For manuscripts utilizing custom algorithms or software that are central to the research but not yet described in published literature, software must be made available to editors and reviewers. We strongly encourage code deposition in a community repository (e.g. GitHub). See the Nature Portfolio guidelines for submitting code & software for further information.

## Data

Policy information about availability of data

All manuscripts must include a data availability statement. This statement should provide the following information, where applicable:

- Accession codes, unique identifiers, or web links for publicly available datasets
- A description of any restrictions on data availability
- For clinical datasets or third party data, please ensure that the statement adheres to our policy

Authors can confirm that all relevant data are included in the article and/or its supplementary information files.
we have included a Data availability section to mention that 'Authors confirm that all relevant data are included in the article and/or its supplementary information files, i.e. datasets used in the study along with appropriately accessible links/accession-codes. Source data are provided with this paper.'

# Field-specific reporting

Please select the one below that is the best fit for your research. If you are not sure, read the appropriate sections before making your selection.

[x] Life sciences [ ] Behavioural & social sciences [ ] Ecological, evolutionary & environmental sciences

For a reference copy of the document with all sections, see nature.com/documents/nr-reporting-summary-flat.pdf

# Life sciences study design

All studies must disclose on these points even when the disclosure is negative.

| | |
|---|---|
| Sample size | For the patient cohort analysis, our outcome variable is a frequency circulating in a beta-distribution. Since it falls within the standard unit interval ] 0; 1 [, we, in a preliminary study, logit-transformed the frequency and performed an Anova power test on some randomly selected biomarkers in order to assess the minimum sample size required in each group. With a power test (probability 1-β) = 0.80, alpha risk = 0.05 and Effect Size = 0.94 (ES = the difference between the largest and smallest means divided by the square root of the mean square error). Our result showed that we need at least 5 people in each patient group. The minimum observed in our data = 8, for the Rea group.<br>Thus, we are confident that no significant sample size problem has occurred.<br>For in vitro experiments, the number of used samples was sufficient to reach required statistical power for all of our statistical tests. Our rational is that when there are not enough samples, the test will not reject our null hypothesis (H0) regardless of the data, regardless H0 is true or false. Thus, before proceeding with the statistical analysis, we ensure by mathematical simulation that H0 can be rejected using our sample sizes when H0 is actually false. To this aim, we generate highly contrasted dummy data having the same shape as our observed data (i.e., same number of samples and same number of experimental conditions). We test the dummy data, if the test cannot reject H0 despite maximum contrasts, this means that the sample size is not sufficient for the choosen test, then the analysis is stopped and no result is presented.<br>In addition, there is not assumption on the shape distribution (i.e., small sample size) and thus we used non-parametric test. |
| Data exclusions | Data exclusion of flow cytometry analysis when the total count of cells were too low to be accurate when analyzed in the gated population.. |
| Replication | The reproducibility of the results was addressed by performing at least three independent experiments and using for each one cells collected from distinct donors. The numbers of independent experiments are indicated in the figure legend section. The results show the means of the parameters measured in the independent experiments, with error-bars representing the standard deviations. When appropriate, each individual experiment is represented by a single dot or symbol, as indicated in the legends. |
| Randomization | The constitution of the cohort was done by the collaboration of the Hospices Civils de Lyon (HCL). This cohort consists of patient groups recognized by clinicians as:<br>i) patients admitted in intensive care units for severe disease at hospital admission (i.e., acute respiratory distress syndrome or severe pneumonia requiring mechanical ventilation, sepsis and septic shock) are referred to as Severe group<br>ii) patients with mild symptoms (i.e., low-grade fever, cough, malaise, rhinorrhea, sore throat) are referred to as Mild early group when collected in the first two weeks and Mild late group for later time points.<br><br>For in vitro test, assigning samples in different experimental conditions was completely random because: i/ our data were homogeneous across factors that can substantially affect our measurements and ii/, our study had an unidimential effect to test. In addition, the collected cells were carefully divided and put in each well without preconceptions before their subjection to the different experimental conditions. Then cells underwent treatments in the same environmental circumstances, i.e., same time, laboratory bench, instruments for the analysis, experimenter etc... to avoid introducing any known technical bias. Thus, statistical analysis valides that the treatment is solely responsible for the biological response and test for the treatment effect without correcting for potential interfering technical bias. |
| Blinding | The processing of samples for flow cytometry analysis was performed with coded samples, thus investigators were blinded to group allocation during data collection.<br>For other types of experiments the blinding was not possible and/or not relevant (e.g., analysis with automatic measurement for ELISA, RT-qPCR etc..). |

# Reporting for specific materials, systems and methods

We require information from authors about some types of materials, experimental systems and methods used in many studies. Here, indicate whether each material, system or method listed is relevant to your study. If you are not sure if a list item applies to your research, read the appropriate section before selecting a response.

## Materials & experimental systems

| n/a | Involved in the study |
|---|---|
| ☐ | ☒ Antibodies |
| ☐ | ☒ Eukaryotic cell lines |
| ☒ | ☐ Palaeontology and archaeology |
| ☒ | ☐ Animals and other organisms |
| ☐ | ☒ Human research participants |
| ☐ | ☒ Clinical data |
| ☒ | ☐ Dual use research of concern |

## Methods

| n/a | Involved in the study |
|---|---|
| ☒ | ☐ ChIP-seq |
| ☐ | ☒ Flow cytometry |
| ☒ | ☐ MRI-based neuroimaging |

## Antibodies

Antibodies used

mouse anti-human CD2 Bv785-conjugated (clone RPA-2.10) BioLegend Cat# 300234, RRID:AB_2800717 2.5 µg/mL
mouse anti-human CD5 A700-conjugated (clone UCHT2 ) BioLegend Cat# 300632, RRID:AB_2632671 1.25 µg/mL
mouse anti-human CD70 PE/Dazzle594-conjugated (clone 113-16) BioLegend Cat# 355123, RRID:AB_2820005 3.33 µg/mL
mouse anti-human CD80 APC-H7-conjugated (clone L307.4) BD Biosciences Cat# 561134, RRID:AB_10565974 10-fold dilution
mouse anti-human CD83 PE/Dazzle594-conjugated (clone HB15e) BioLegend Cat# 305328, RRID:AB_2564260 10 µg/mL
mouse anti-human CD123 Bv711-conjugated (clone 6H6) BioLegend Cat# 306030, RRID:AB_2566354 5 µg/mL
mouse anti-humain CD274/PE-Cy™7 (clone MIH1) BD Biosciences Cat# 558017, RRID:AB_396986 10 µg/mL
mouse anti-human CD303 Bv421-conjugated (clone 201A) BioLegend Cat# 354212, RRID:AB_2563871 10 µg/mL
mouse anti- human HLA-DR Bv510-conjugated (clone L243) BioLegend Cat# 307646, RRID:AB_2561948 1.2 µg/mL
mouse anti-human TNFa PE-conjugated (clone MAb11) BioLegend Cat# 502909, RRID:AB_315261 10 µg/mL
mouse anti-human TRAIL PE-conjugated (clone RIK-2 ) BioLegend Cat# 308205, RRID:AB_345291 10 µg/mL
mouse anti-human IFNa APC-conjugated (clone LT27:295) Miltenyi Biotec Cat# 130-092-602, RRID:AB_871558 10-fold dilution
mouse anti-human IL-29 (IFN-λ1) purified (Clone 247801) R and D Systems Cat# MAB15981, RRID:AB_2125340 200 µg/mL
mouse anti-human AXL A488-conjugated (clone 108724R) R and D Systems Cat# FAB154RG 10 µg/mL
mouse anti-human CD123 PE or APC-conjugated (clone AC145) Miltenyi Cat# 130-090-901, RRID:AB_244209 20-fold dilution
mouse anti-human BDCA-2 APC-conjugated (clone AC144) Miltenyi Cat# 130-090-905, RRID:AB_244165 20-fold dilution
mouse anti-human CD11c BV605-conjugated (clone B-ly6) BD Horizon Cat# 563929, RRID:AB_2744276 50-fold dilution
mouse anti-human CD56/NCAM FITC-conjugated (clone TULY56) eBiscience Cat# 11-0566-42, RRID:AB_2572459 5 µg/mL
mouse anti-human HLA-DR BV711-conjugated (clone L243) BioLegend Cat# 307644, RRID:AB_2562913 1 µg/mL
mouse anti-human IL-6 PE-conjugated (clone MQ2-13A5) BioLegend Cat# 501107, RRID:AB_315155 2,5 µg/mL
mouse anti-human CD14 BV785-conjugated (clone M5E2 ) BioLegend Cat# 301840, RRID:AB_2563425 5 µg/mL
mouse anti-human CD14 FITC-conjugated (clone M5E2) BD Pharmingen Cat# 555397, RRID:AB_395798 20-fold dilution
mouse anti-human CD141 PerCP/Cy5.5-conjugated (clone M80) Biolegend Cat# 344112, RRID:AB_2561625 20 µg/mL
mouse anti-human CD16 FITC-conjugated (clone B73.1) BioLegend Cat# 360716, RRID:AB_2563071 20 µg/mL
mouse anti-human CD16 PacificBlue-conjugated (clone 3G8) Biolegend Cat# 302032, RRID:AB_2104003 10 µg/mL
mouse anti-human CD19 FITC-conjugated (clone HIB19) eBiscience Cat# 11-0199-42, RRID:AB_10669461 10 µg/mL
mouse anti-human CD1c PerCP-eFluor710-conjugated (clone L161)eBiscience Cat# 46-0015-42, RRID:AB_10548936 1.2 µg/mL
mouse anti-human CD20 FITC-conjugated (clone 2H7) BD Pharmingen Cat# 555622, RRID:AB_395988 20-fold dilution
mouse anti-human CD3 FITC-conjugated (clone UCHT1) eBiscience Cat# 11-0038-42, RRID:AB_2043831 10 µg/mL
mouse anti-dsRNA unconjugated (clone J2 IgG2a) SCICONS Cat# 10010200, RRID:AB_2651015 10 µg/mL
rabbit anti-SARS-CoV-2 Spike S2 unconjugated (polyclonal) Sino Biological 40590-T62-100 200-fold dilution
mouse anti-human integrin αL subunit unconjugated (clone 38) antibodies online Cat# ABIN375486, RRID:AB_10782956 10 µg/mL
mouse anti-human ICAM-1 unconjugated (clone LB-2) BD Pharmingen Cat# 559047, RRID:AB_397183 10 µg/mL
goat anti-Rabbit IgG (H+L) AlexaFluor 546-conjugated Invitrogen Cat# A-11035, RRID:AB_143051 2 µg/mL
goat anti-Rabbit IgG (H+L) AlexaFluor 647-conjugated Invitrogen Cat# A-31573, RRID:AB_2536183 2 µg/mL
goat anti-mouse IgG (H+L) AlexaFluor 555-conjugated Invitrogen Cat# A32727, RRID:AB_2536164 2 µg/mL

Validation

Antibody are commercially available and used according to the manufacturer's protocol/information - Applications: Flow Cytometry
mouse anti-human CD2 Bv785-conjugated (clone RPA-2.10) BioLegend Cat# 300234, RRID:AB_2800717 2.5 µg/mL
mouse anti-human CD5 A700-conjugated (clone UCHT2 ) BioLegend Cat# 300632, RRID:AB_2632671 1.25 µg/mL
mouse anti-human CD70 PE/Dazzle594-conjugated (clone 113-16) BioLegend Cat# 355123, RRID:AB_2820005 3.33 µg/mL
mouse anti-human CD80 APC-H7-conjugated (clone L307.4) BD Biosciences Cat# 561134, RRID:AB_10565974 10-fold dilution
mouse anti-human CD83 PE/Dazzle594-conjugated (clone HB15e) BioLegend Cat# 305328, RRID:AB_2564260 10 µg/mL
mouse anti-human CD123 Bv711-conjugated (clone 6H6) BioLegend Cat# 306030, RRID:AB_2566354 5 µg/mL
mouse anti-humain CD274/PE-Cy™7 (clone MIH1) BD Biosciences Cat# 558017, RRID:AB_396986 10 µg/mL
mouse anti-human CD303 Bv421-conjugated (clone 201A) BioLegend Cat# 354212, RRID:AB_2563871 10 µg/mL

mouse anti- human HLA-DR Bv510-conjugated (clone L243) BioLegend Cat# 307646, RRID:AB_2561948 1.2 µg/mL
mouse anti-human TNFa PE-conjugated (clone MAb11) BioLegend Cat# 502909, RRID:AB_315261 10 µg/mL
mouse anti-human TRAIL PE-conjugated (clone RIK-2 ) BioLegend Cat# 308205, RRID:AB_345291 10 µg/mL
mouse anti-human IFNa APC-conjugated (clone LT27:295) Miltenyi Biotec Cat# 130-092-602, RRID:AB_871558 10-fold dilution
mouse anti-human IL-29 (IFN-λ1) purified (Clone 247801) R and D Systems Cat# MAB15981, RRID:AB_2125340 200 µg/mL
mouse anti-human AXL A488-conjugated (clone 108724R) R and D Systems Cat# FAB154RG 10 µg/mL
mouse anti-human CD123 PE or APC-conjugated (clone AC145) Miltenyi Cat# 130-090-901, RRID:AB_244209 20-fold dilution
mouse anti-human BDCA-2 APC-conjugated (clone AC144) Miltenyi Cat# 130-090-905, RRID:AB_244165 20-fold dilution
mouse anti-human CD11c BV605-conjugated (clone B-ly6) BD Horizon Cat# 563929, RRID:AB_2744276 50-fold dilution
mouse anti-human CD56/NCAM FITC-conjugated (clone TULY56) eBiscience Cat# 11-0566-42, RRID:AB_2572459 5 µg/mL
mouse anti-human HLA-DR BV711-conjugated (clone L243) BioLegend Cat# 307644, RRID:AB_2562913 1 µg/mL
mouse anti-human IL-6 PE-conjugated (clone MQ2-13A5) BioLegend Cat# 501107, RRID:AB_315155 2,5 µg/mL
mouse anti-human CD14 BV785-conjugated (clone M5E2 ) BioLegend Cat# 301840, RRID:AB_2563425 5 µg/mL
mouse anti-human CD14 FITC-conjugated (clone M5E2) BD Pharmingen Cat# 555397, RRID:AB_395798 20-fold dilution
mouse anti-human CD141 PerCP/Cy5.5-conjugated (clone M80) Biolegend Cat# 344112, RRID:AB_2561625 20 µg/mL
mouse anti-human CD16 FITC-conjugated (clone B73.1) BioLegend Cat# 360716, RRID:AB_2563071 20 µg/mL
mouse anti-human CD16 PacificBlue-conjugated (clone 3G8) Biolegend Cat# 302032, RRID:AB_2104003 10 µg/mL
mouse anti-human CD19 FITC-conjugated (clone HIB19) eBiscience Cat# 11-0199-42, RRID:AB_10669461 10 µg/mL
mouse anti-human CD1c PerCP-eFluor710-conjugated (clone L161)eBiscience Cat# 46-0015-42, RRID:AB_10548936 1.2 µg/mL
mouse anti-human CD20 FITC-conjugated (clone 2H7) BD Pharmingen Cat# 555622, RRID:AB_395988 20-fold dilution
mouse anti-human CD3 FITC-conjugated (clone UCHT1) eBiscience Cat# 11-0038-42, RRID:AB_2043831 10 µg/mL

Antibody are commercially available and used according to the manufacturer's protocol/information – Applications : Flow cytometry & imaging
goat anti-Rabbit IgG (H+L) AlexaFluor 546-conjugated Invitrogen Cat# A-11035, RRID:AB_143051 2 µg/mL
goat anti-Rabbit IgG (H+L) AlexaFluor 647-conjugated Invitrogen Cat# A-31573, RRID:AB_2536183 2 µg/mL
goat anti-mouse IgG (H+L) AlexaFluor 555-conjugated Invitrogen Cat# A32727, RRID:AB_2536164 2 µg/mL

Validated by serial dilution using uninfected cells as neg control – Applications : Flow cytometry & imaging
mouse anti-dsRNA unconjugated (clone J2 IgG2a) SCICONS Cat# 10010200, RRID:AB_2651015 10 µg/mL
rabbit anti-SARS-CoV-2 Spike S2 unconjugated (polyclonal) Sino Biological 40590-T62-100 200-fold dilution

Validated by serial dilution using oculture without pDC as neg control – Application : Coculture
mouse anti-human integrin αL subunit unconjugated (clone 38) antibodies online Cat# ABIN375486, RRID:AB_10782956 10 µg/mL
mouse anti-human ICAM-1 unconjugated (clone LB-2) BD Pharmingen Cat# 559047, RRID:AB_397183 10 µg/mL

## Eukaryotic cell lines

Policy information about cell lines

| | |
|---|---|
| Cell line source(s) | A549 cell line ATCC CCL-185<br>Calu-3 cell line ATCC HTB-55<br>NCI-H358 cell line ATCC CRL-5807<br>293T cell line ATCC CRL-3216<br>Human hepatoma cell line Nakabayashi et al., 1982 Huh-7.5.1 (RRID:CVCL_E049)<br>Vero cell line (clone Vero E6) Dr M Bouloy; Institut Pasteur, France ATCC CRL-1586 |
| Authentication | Cell lines were from ATCC, already referenced. No additional authentication was performed. |
| Mycoplasma contamination | All cell line were test negative for Mycoplasma contamination |
| Commonly misidentified lines<br>(See ICLAC register) | None |

# Human research participants

Policy information about studies involving human research participants

| | |
|---|---|
| Population characteristics | Symptomatic health care workers (COVID-SER patients) and patients admitted in ICU positive for COVID-19 (COVID-rea patients) were included in a prospective longitudinal cohort study conducted in the Hospices Civils de Lyon (Lyon, France). Demographic characteristics are depicted in Table 1. |
| Recruitment | Consent was obtained from all participants.<br>The participants were recruited at the hospital (Hospices Civils de Lyon, France) and without criteria of exclusion other than pregnancy.<br>The blood units from adult human healthy donors were obtained according to procedures approved by the "Etablissement Français du sang" (EFS) Committee. |
| Ethics oversight | COVID-SER patients: For the mild adult COVID-19 cohort, the clinical study registered on ClinicalTrial.gov (NCT04341142) has been fully detailed 48. In the present study, only patients with mild symptoms of COVID-19 were included. Written informed consent was obtained from all participants and approval was obtained from the national review board for biomedical research in April 2020 (Comité de Protection des Personnes Sud Méditerranée I, Marseille, France; ID RCB 2020-A00932-37).<br><br>COVID-rea patients: For the severe adult COVID-19 cohort, the study was registered to the French National Data Protection Agency under the number 20-097 and was approved by an ethical committee for biomedical research (Comité de Protection des Personnes HCL) under the number N°20-41. In agreement with the General Data Protection Regulation (Regulation (EU) 2016/679 and Directive 95/46/EC) and the French data protection law (Law n°78-17 on 06/01/1978 and Décret n°2019-536 on 29/05/2019), we obtained consent from each patient or his/her next of kin.<br><br>Blood samples from healthy donors were used as reference and experimentally processed similarly. These samples were obtained from the national blood service, called 'Etablissement Français du sang' (EFS) according to stand-ardized procedures for blood donation approved by the EFS Committee and followed provisions of articles R.1243–49 and the French public health code to obtain written non-opposition to the use of donated blood for research purposes from healthy volunteers. The personal data were deidentified before transfer to our research labor-atory. We obtained the favorable notice of the local ethics committee (Comité de Protection des Personnes Sud-Est II, Bâtiment Pinel, 59 Boulevard Pinel, 69 500 Bron) and acceptance from the French ministry of research (Ministère de l'Enseignement supérieur, de la Recherche et de l'Innovation, DC-2008-64) for the handling and conserva-ion of these samples. To limit the risk of inclusion of asymptotic healthy donor: i/ part of the blood samples was collected prior to the pandemic and ii/ for blood collected during the SARS-CoV-2 pandemic, systemic examination and questioning/interview of the donors were performed and included symptoms, prior contacts at risk and vaccination, Thus, blood samples were excluded from our study if ongoing and/or recent COVID-positivity was suspected. |

Note that full information on the approval of the study protocol must also be provided in the manuscript.

# Clinical data

Policy information about clinical studies

All manuscripts should comply with the ICMJEguidelines for publication of clinical research and a completedCONSORT checklist must be included with all submissions.

| | |
|---|---|
| Clinical trial registration | NCT04341142 |
| Study protocol | For mild covid-19 subjects, we described the protocol (https://bmjopen.bmj.com/content/10/11/e041268). |
| Data collection | For immunological analyses, clinical data were collected using the Clinsight software, during January-April 2021.<br>Blood samples were processed and stored at the Centre de Ressource Biologique Neurobiotec, 69500 Bron.<br>Serological and immunological analyses were performed at the Lyon-Sud hospital or at the Centre International de recherche en infectiologie (CIRI) in Lyon. |
| Outcomes | We predefined a panel of cell type and activation/response marker that were analysed for all samples by flow cytometry. |

# Flow Cytometry

## Plots

Confirm that:

- [x] The axis labels state the marker and fluorochrome used (e.g. CD4-FITC).
- [x] The axis scales are clearly visible. Include numbers along axes only for bottom left plot of group (a 'group' is an analysis of identical markers).
- [x] All plots are contour plots with outliers or pseudocolor plots.
- [x] A numerical value for number of cells or percentage (with statistics) is provided.

## Methodology

| | |
|---|---|
| Sample preparation | At the indicated times, harvested cells were resuspended using 2 mM EDTA-PBS solution for the coculture with PBMCs and 0.48 mM EDTA-PBS solution for pDC cocultures. Cells were incubated with 1 µL/mL viability marker diluted in PBS for 20 minutes at RT. After a 10-minute incubation with Fc receptor blocking reagent (MACS Miltenyi Biotec) at 4°C followed by two PBS washes, cells were stained for surface markers for 30 minutes at 4°C with antibodies diluted in staining buffer (PBS without calcium and magnesium, with 2% FBS and 2mM EDTA), followed by two PBS washes. For the identification of apoptotic and necrotic cells, surface-stained cells were labelled using FITC Annexin V Apoptosis Detection Kit with 7-AAD (BioLegend) according to the manufacturer's instructions, and then were fixed with 4% PFA for 30 minutes at 4°C. For intracellular-immunostaining, after surface staining and fixation with cytoperm/cytofix solution (BD Bioscience) for 20 minutes at 4°C, IFNα, IL6, IFNλ1, and TNFα were stained by a 45-minute incubation at 4°C with antibodies diluted in permeabilization buffer (BD Bioscience ; antibodies are listed in the Key resource table). Cells were then washed with permeabilization buffer and resuspended in staining buffer. |
| Instrument | Flow cytometric analysis was performed using a BD LSR Fortessa 4L |
| Software | Compensation beads were used as reference for the analysis. The data were analyzed using Flow Jo software (Tree Star). |
| Cell population abundance | pDCs used in our study were positively selected from PBMCs using BDCA-4-magnetic beads (MACS Miltenyi Biotec) as previously described (Décembre et al., PLoS Pathog.2014,doi:10.1371/journal.ppat.1004434; Assil et al., Cell Host Microbe.2019,doi: 10.1016/j.chom.2019.03.005). The purity of immuno-isolated lived pDCs was ≤90% as assessed by viability dye and gating using anti-CD123 and anti-BDCA2 (along with negative for anti-CD11c and BDCA3) specific markers. |
| Gating strategy | pDCs were gated as (see Supplementary Figures S1A and S3A):<br>- live cells : FSC-A (20K-150K), negative for viability marker (10^2 to 103)<br>- singulets : SSC-H (20K-100K), SSC-A (20K-120K)<br>- BDCA2+ (6.10^2-10^4) CD123+ (7.10^3-10^5) cells |

☒ Tick this box to confirm that a figure exemplifying the gating strategy is provided in the Supplementary Information.
