# Supplementary figures and images for: Severe COVID-19 patients have impaired plasmacytoid dendritic cell-mediated control of SARS-CoV-2
Source: Nat Commun. 2023 Feb 8;14:694. doi: 10.1038/s41467-023-36140-9 (PMC9907212; doi:10.1038/s41467-023-36140-9)

Parameters importance

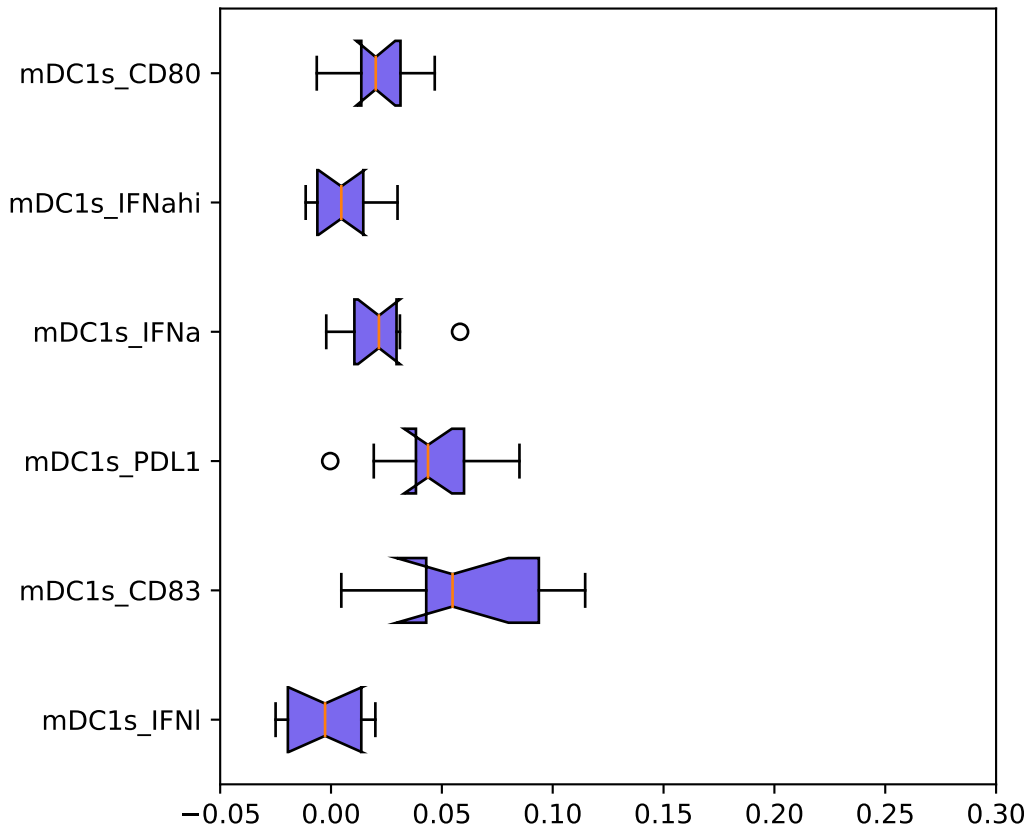

Supplement: Supplementary file 4 — Source Data [file 41467_2023_36140_MOESM4_ESM.zip › Source data/Venet Fig 2b_mDC1s_def/Parameters_importance_Validation_mean.pdf]

Accuracy over the Build datasets sets  
mean = 0.47 +/- 0.08

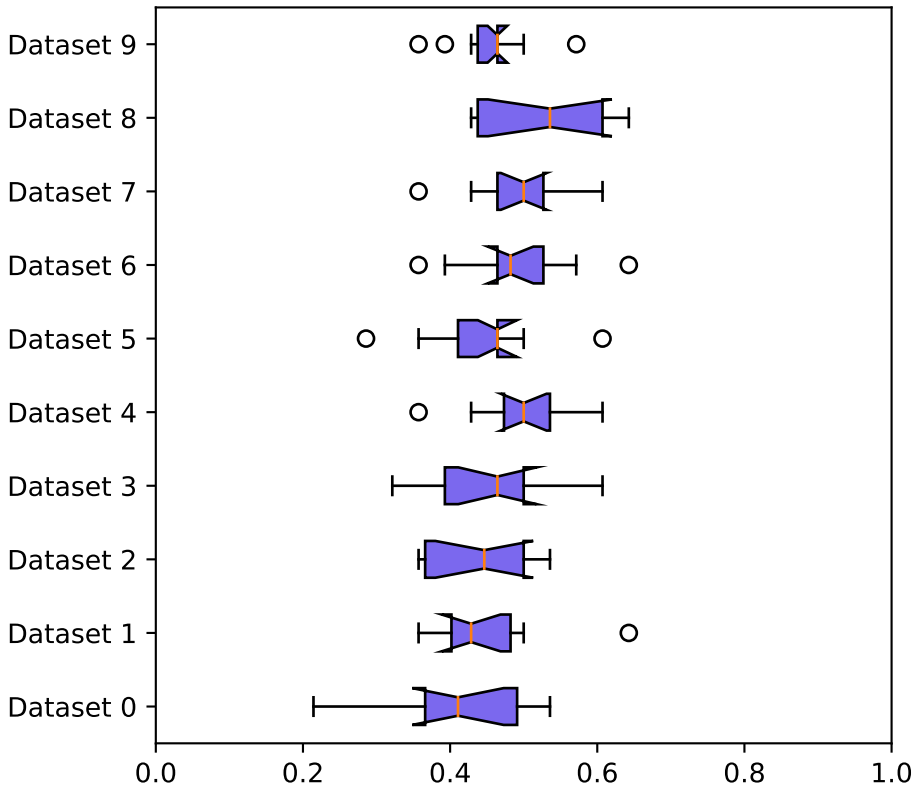

Supplement: Supplementary file 4 — Source Data [file 41467_2023_36140_MOESM4_ESM.zip › Source data/Venet Fig 2b_mDC1s_def/Tracking_Validation_accuracies.pdf]

# Accuracy over the Build datasets sets

mean = 0.93 +/- 0.04

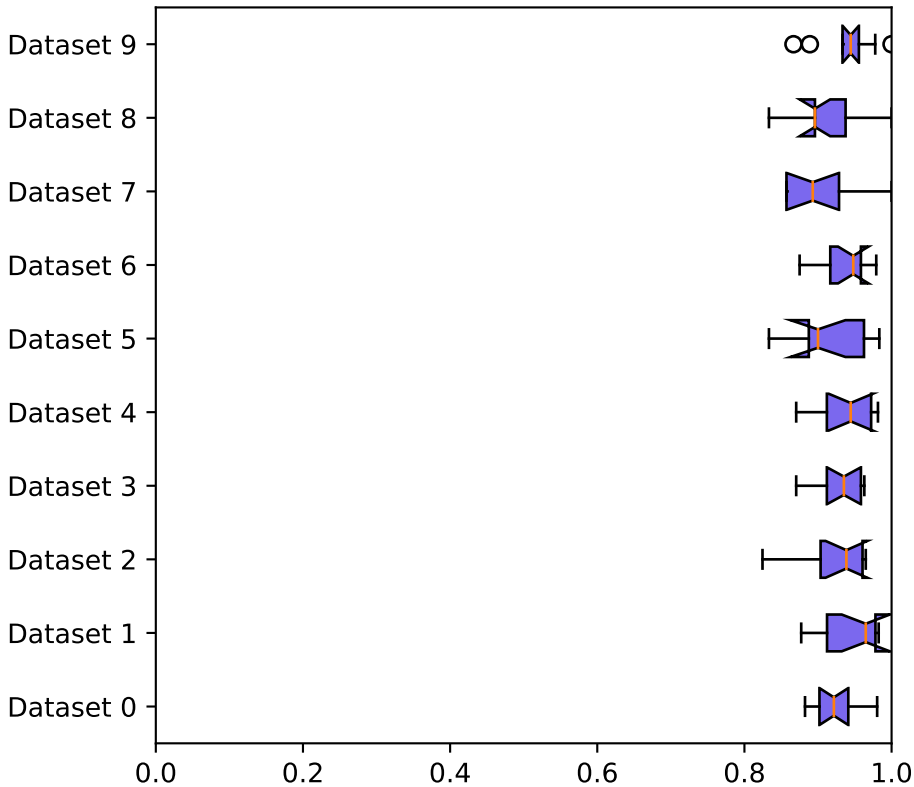

Supplement: Supplementary file 4 — Source Data [file 41467_2023_36140_MOESM4_ESM.zip › Source data/Venet Fig 2b_mDC1s_def/Tracking_Build_accuracies.pdf]

Parameters importance

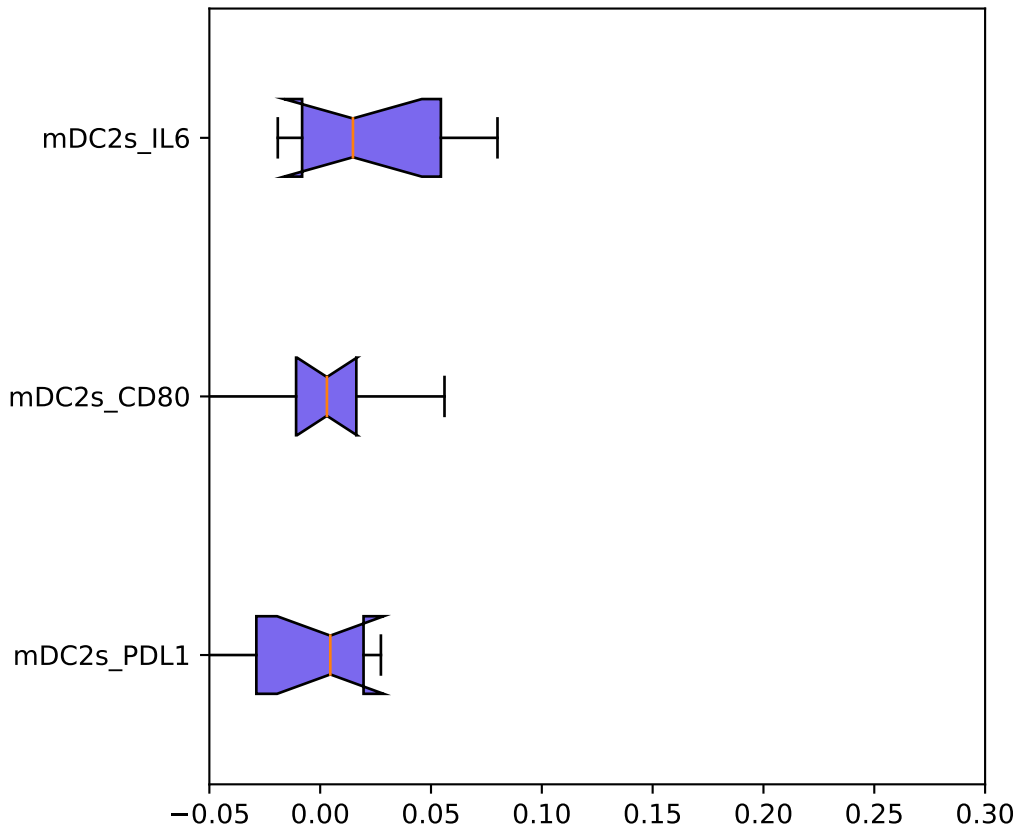

Supplement: Supplementary file 4 — Source Data [file 41467_2023_36140_MOESM4_ESM.zip › Source data/Venet Fig 2b_mDC2s_def/Parameters_importance_Validation_mean.pdf]

Accuracy over the Build datasets sets  
mean = 0.34 +/- 0.11

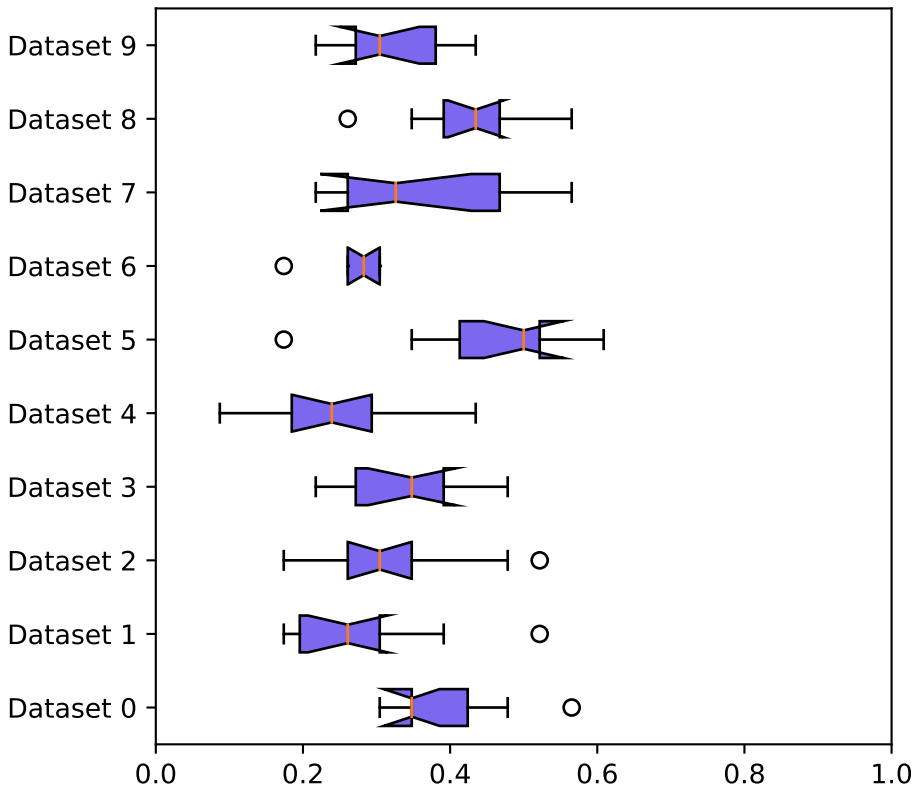

Supplement: Supplementary file 4 — Source Data [file 41467_2023_36140_MOESM4_ESM.zip › Source data/Venet Fig 2b_mDC2s_def/Tracking_Validation_accuracies.pdf]

Accuracy over the Build datasets sets  
mean = 0.88 +/- 0.06

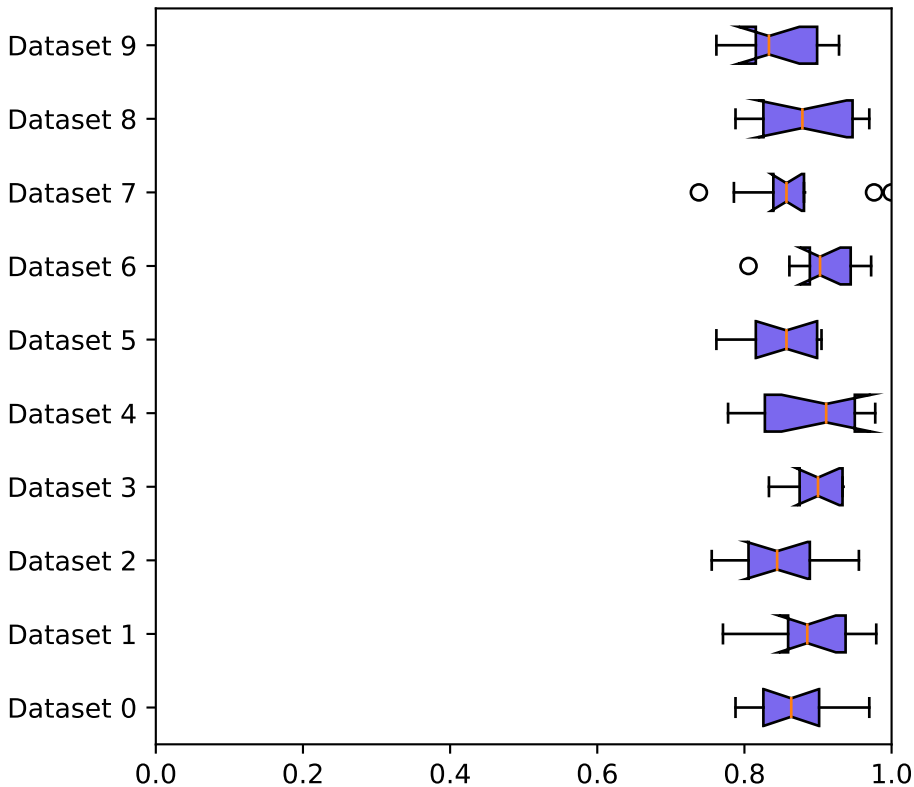

Supplement: Supplementary file 4 — Source Data [file 41467_2023_36140_MOESM4_ESM.zip › Source data/Venet Fig 2b_mDC2s_def/Tracking_Build_accuracies.pdf]

Parameters importance

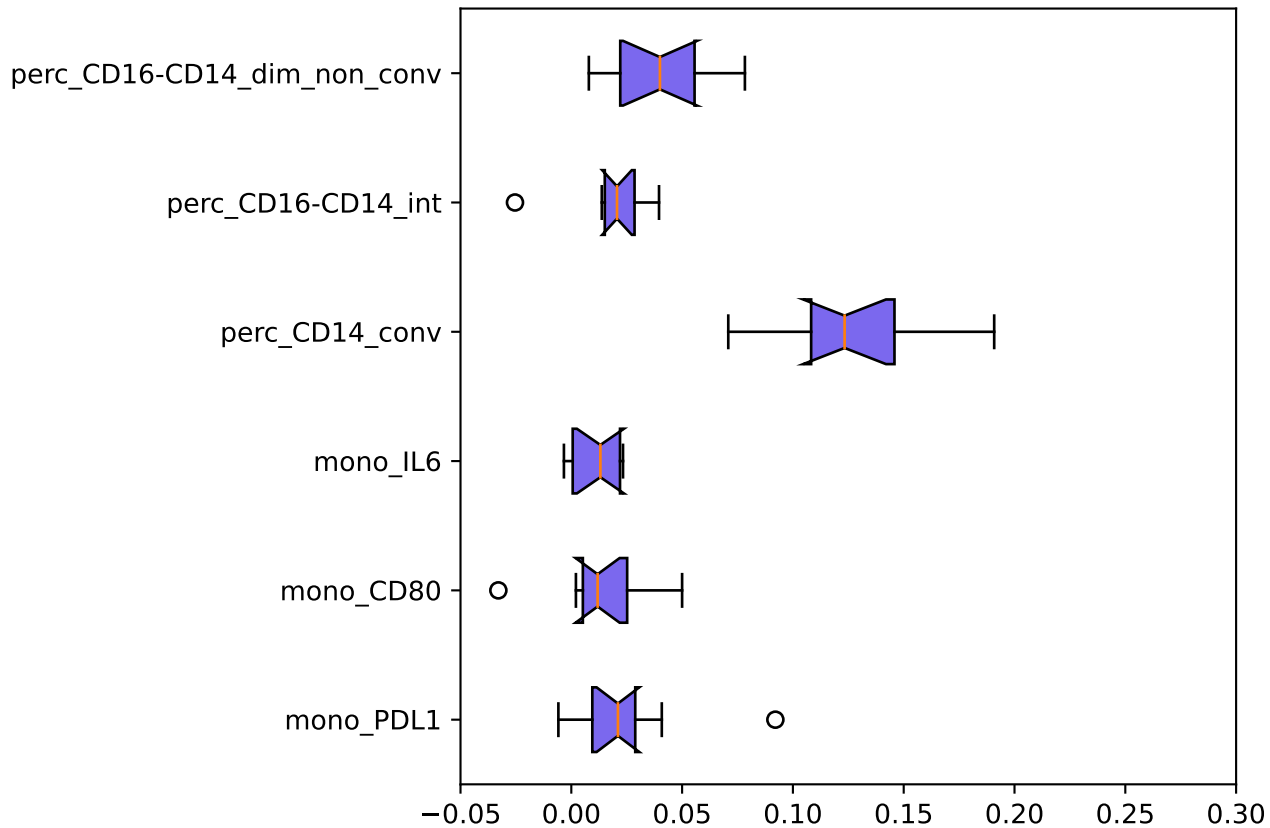

Supplement: Supplementary file 4 — Source Data [file 41467_2023_36140_MOESM4_ESM.zip › Source data/Venet Fig 2b_monos_def/Parameters_importance_Validation_mean.pdf]

Accuracy over the Build datasets sets  
mean = 0.48 +/- 0.09

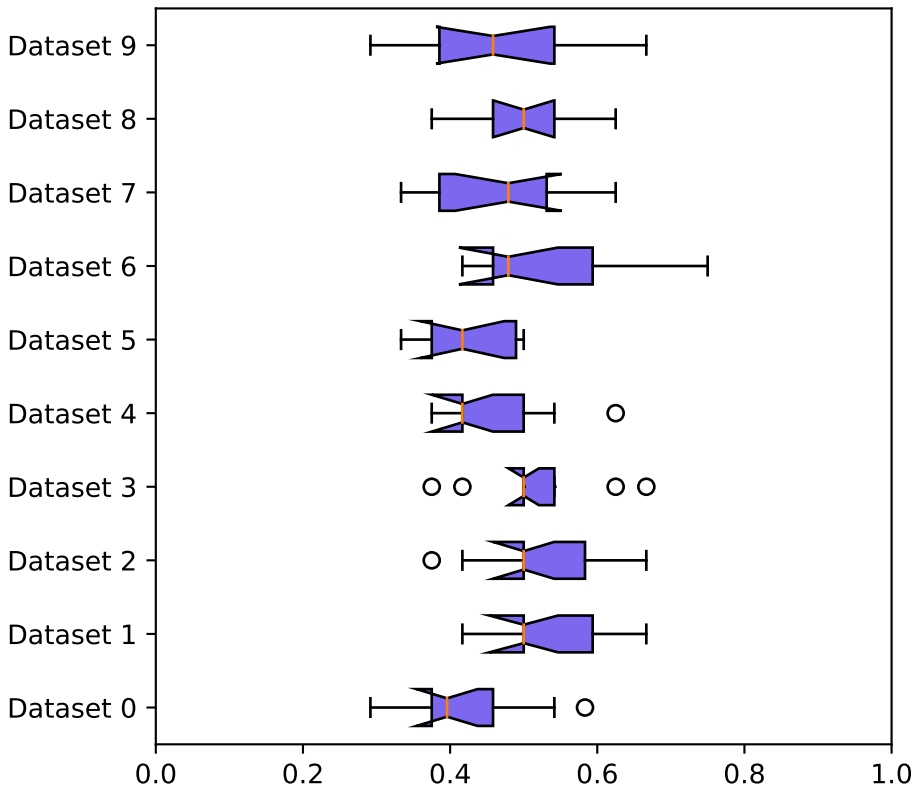

Supplement: Supplementary file 4 — Source Data [file 41467_2023_36140_MOESM4_ESM.zip › Source data/Venet Fig 2b_monos_def/Tracking_Validation_accuracies.pdf]

Accuracy over the Build datasets sets  
mean = 0.94 +/- 0.04

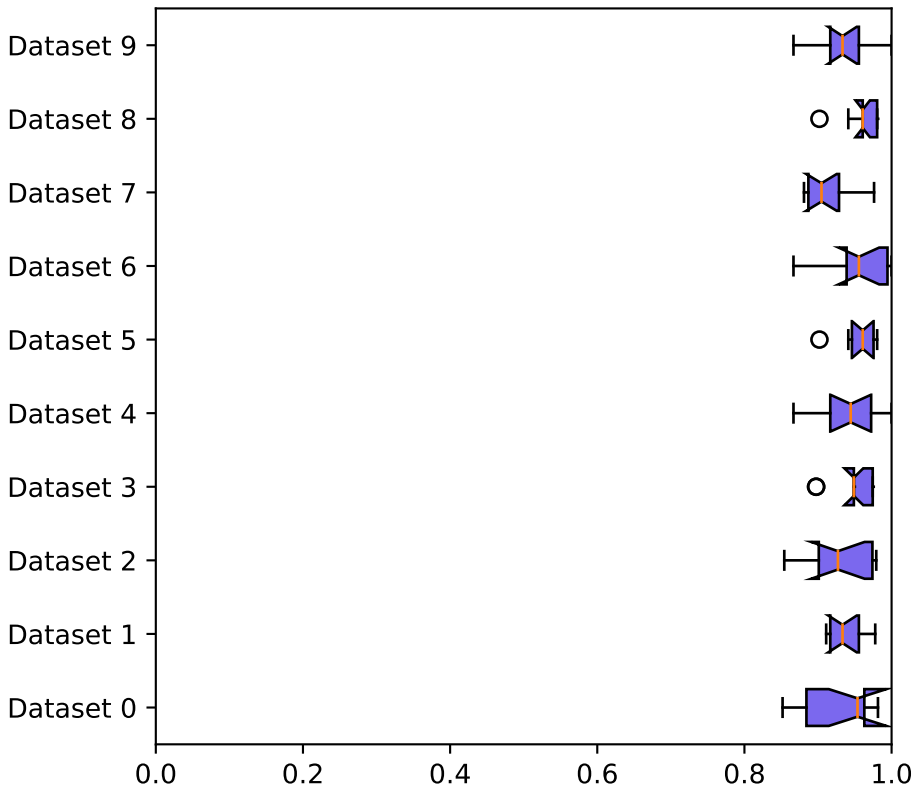

Supplement: Supplementary file 4 — Source Data [file 41467_2023_36140_MOESM4_ESM.zip › Source data/Venet Fig 2b_monos_def/Tracking_Build_accuracies.pdf]

Parameters importance

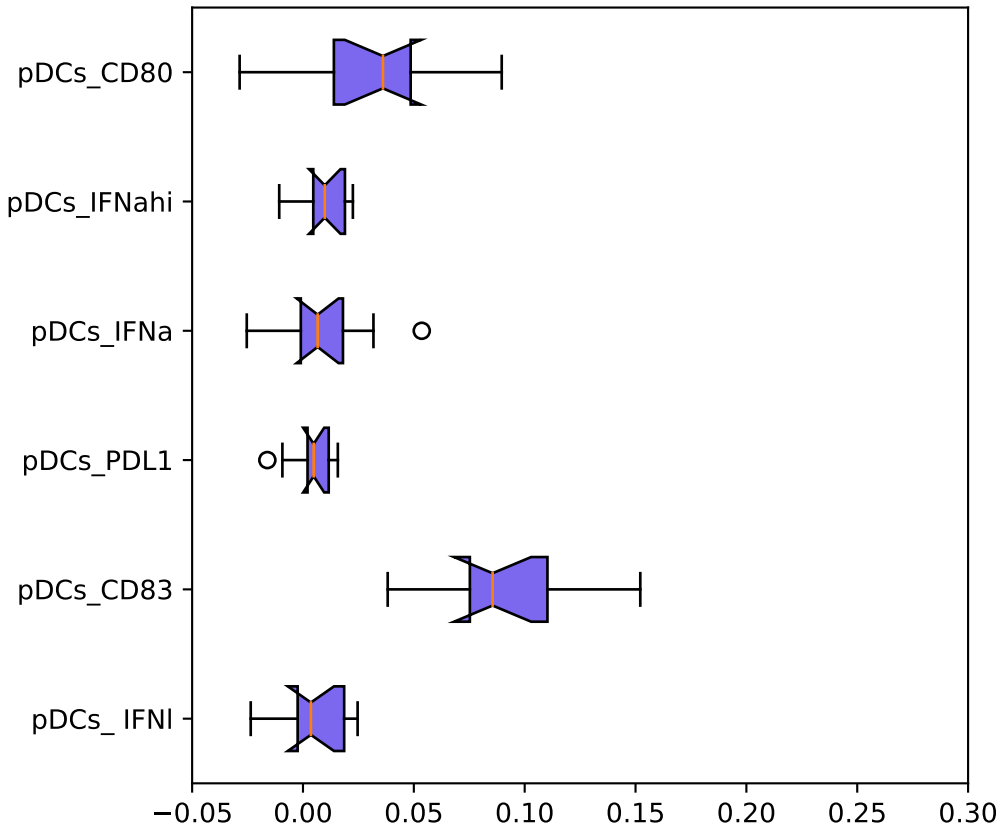

Supplement: Supplementary file 4 — Source Data [file 41467_2023_36140_MOESM4_ESM.zip › Source data/Venet Fig 2b_pDCs_def/Parameters_importance_Validation_mean.pdf]

Accuracy over the Build datasets sets  
mean = 0.5 +/- 0.1

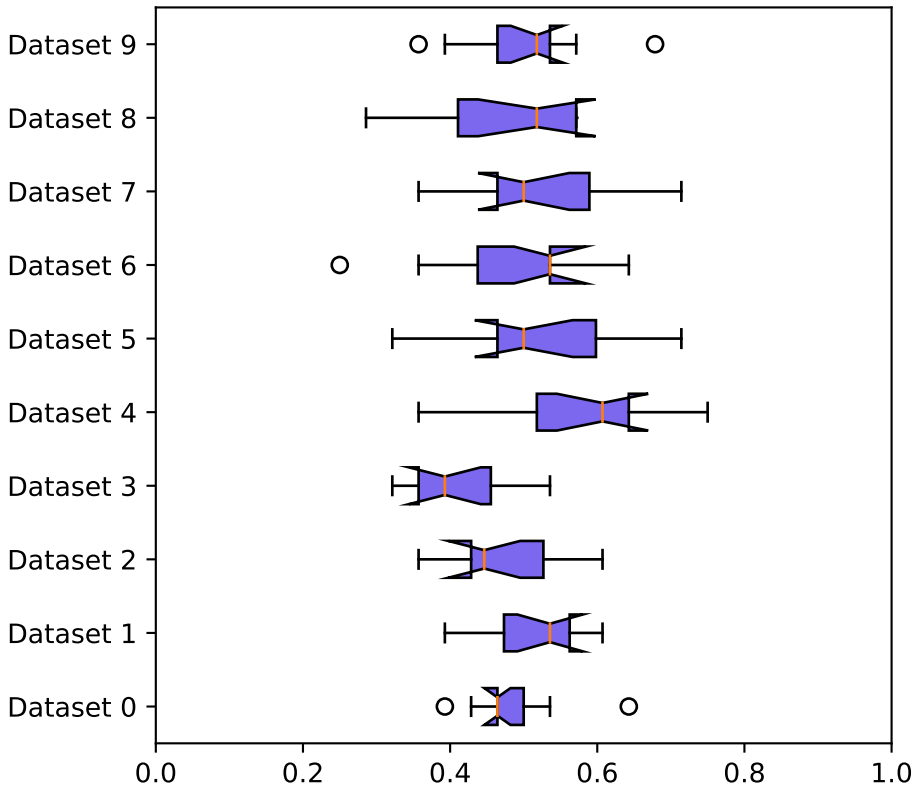

Supplement: Supplementary file 4 — Source Data [file 41467_2023_36140_MOESM4_ESM.zip › Source data/Venet Fig 2b_pDCs_def/Tracking_Validation_accuracies.pdf]

Accuracy over the Build datasets sets  
mean = 0.93 +/- 0.04

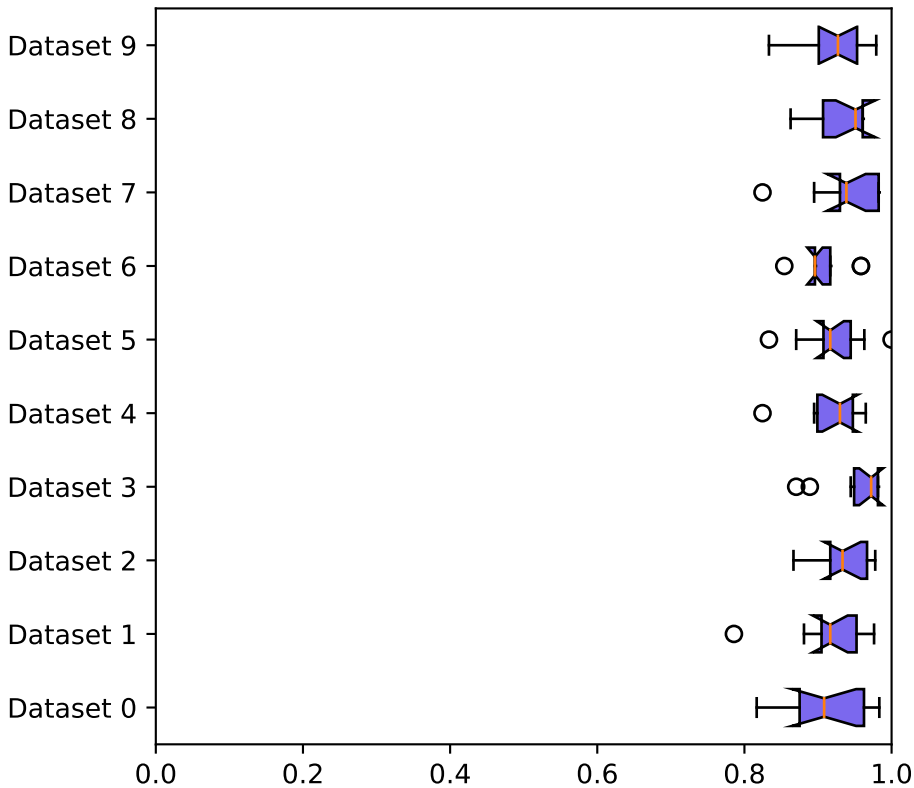

Supplement: Supplementary file 4 — Source Data [file 41467_2023_36140_MOESM4_ESM.zip › Source data/Venet Fig 2b_pDCs_def/Tracking_Build_accuracies.pdf]

Confusion matrix Downsampling 7

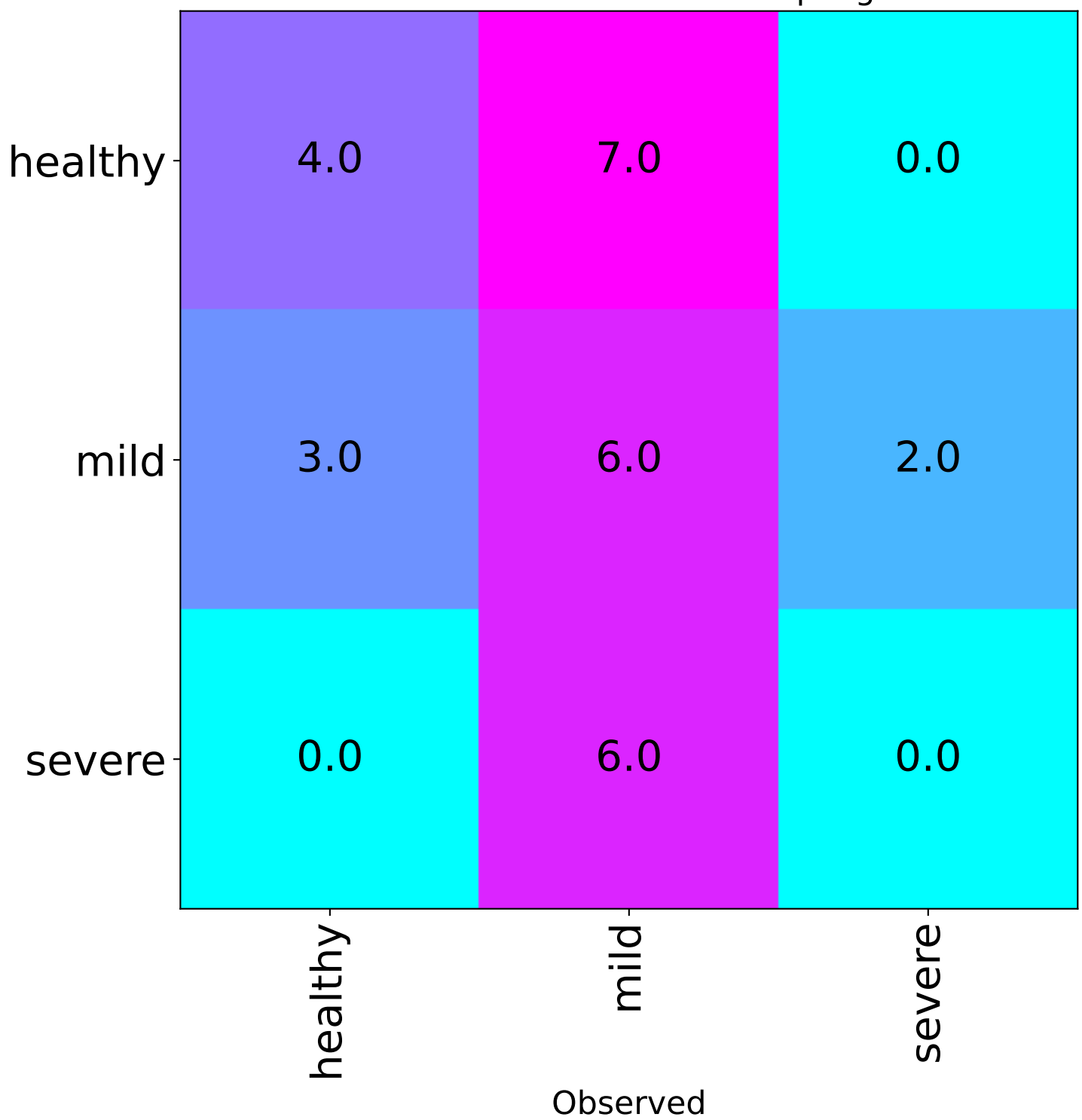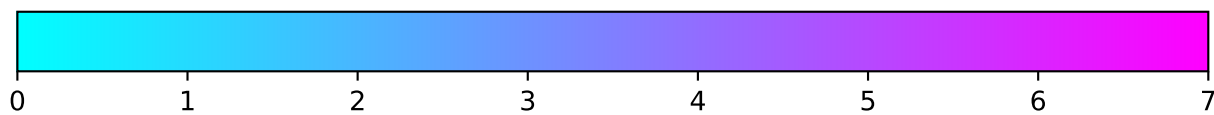

Supplement: Supplementary file 4 — Source Data [file 41467_2023_36140_MOESM4_ESM.zip › Source data/Venet Fig 2b_mDC1s_def/2022-04-28_Thu_16-59-36_GradientBoostClassifier_6/Confusion_matrix_Downsampling 7.pdf]

Confusion matrix Downsampling 4

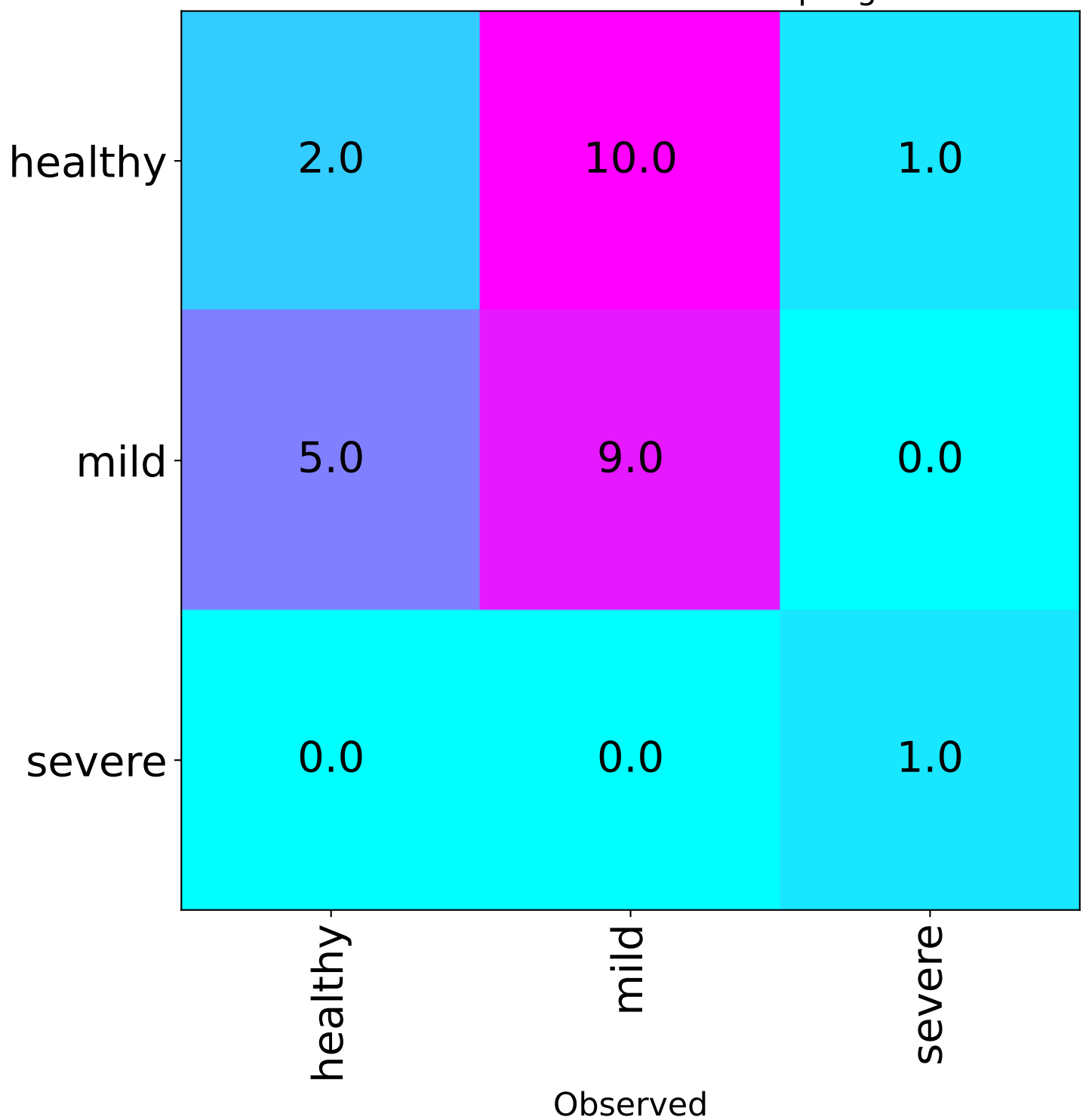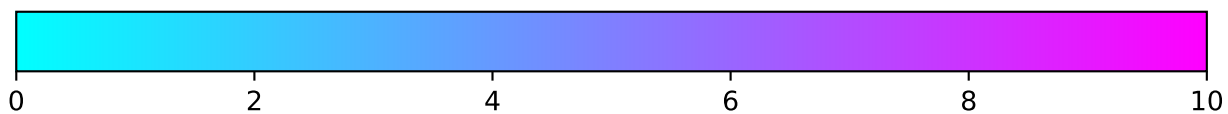

Supplement: Supplementary file 4 — Source Data [file 41467_2023_36140_MOESM4_ESM.zip › Source data/Venet Fig 2b_mDC1s_def/2022-04-28_Thu_16-59-36_GradientBoostClassifier_6/Confusion_matrix_Downsampling 4.pdf]

Confusion matrix Downsampling 5

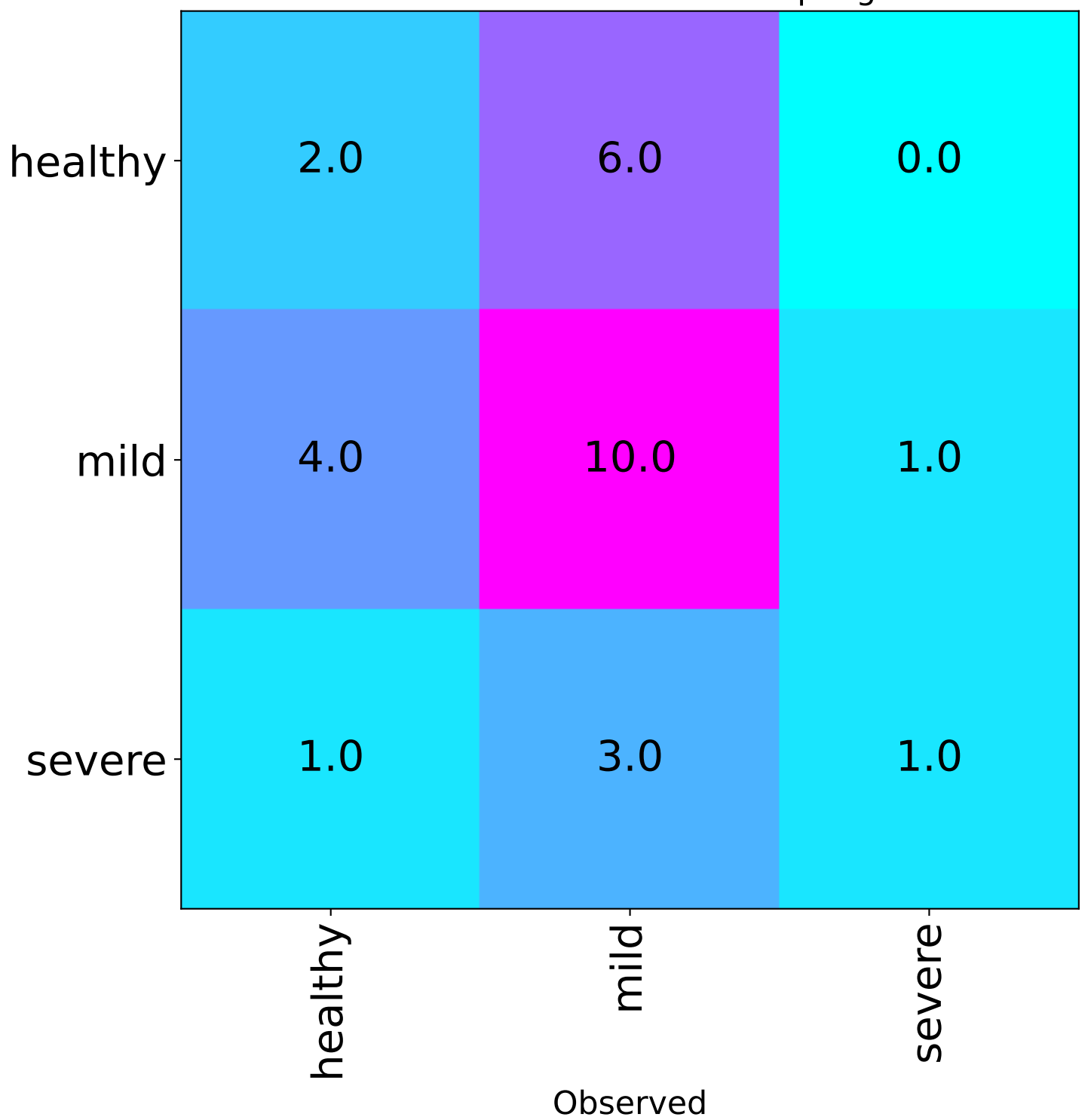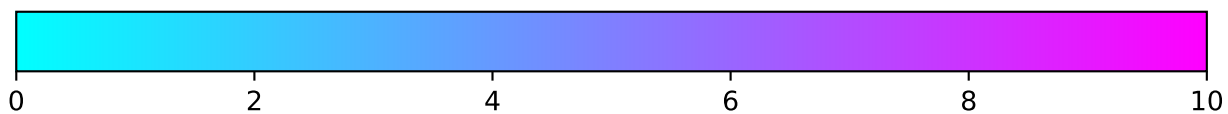

Supplement: Supplementary file 4 — Source Data [file 41467_2023_36140_MOESM4_ESM.zip › Source data/Venet Fig 2b_mDC1s_def/2022-04-28_Thu_16-59-36_GradientBoostClassifier_6/Confusion_matrix_Downsampling 5.pdf]

Confusion matrix Downsampling 1

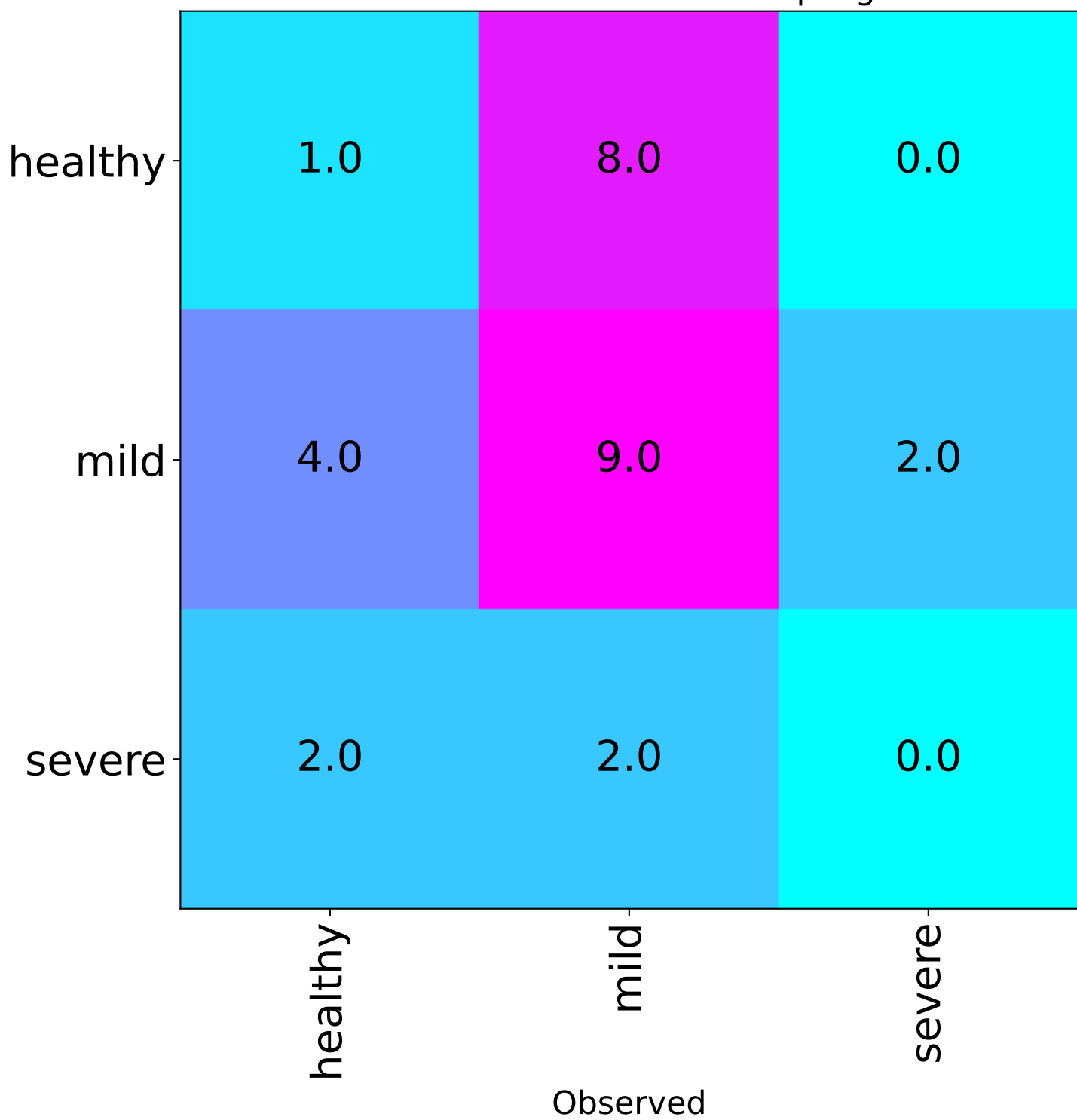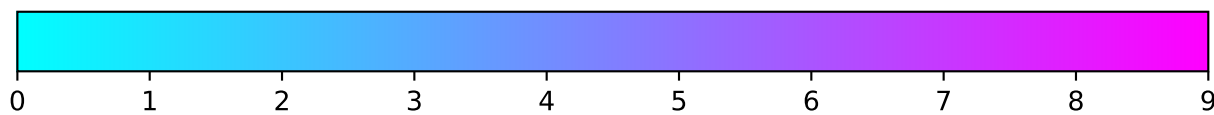

Supplement: Supplementary file 4 — Source Data [file 41467_2023_36140_MOESM4_ESM.zip › Source data/Venet Fig 2b_mDC1s_def/2022-04-28_Thu_16-59-36_GradientBoostClassifier_6/Confusion_matrix_Downsampling 1.pdf]

Confusion matrix Downsampling 2

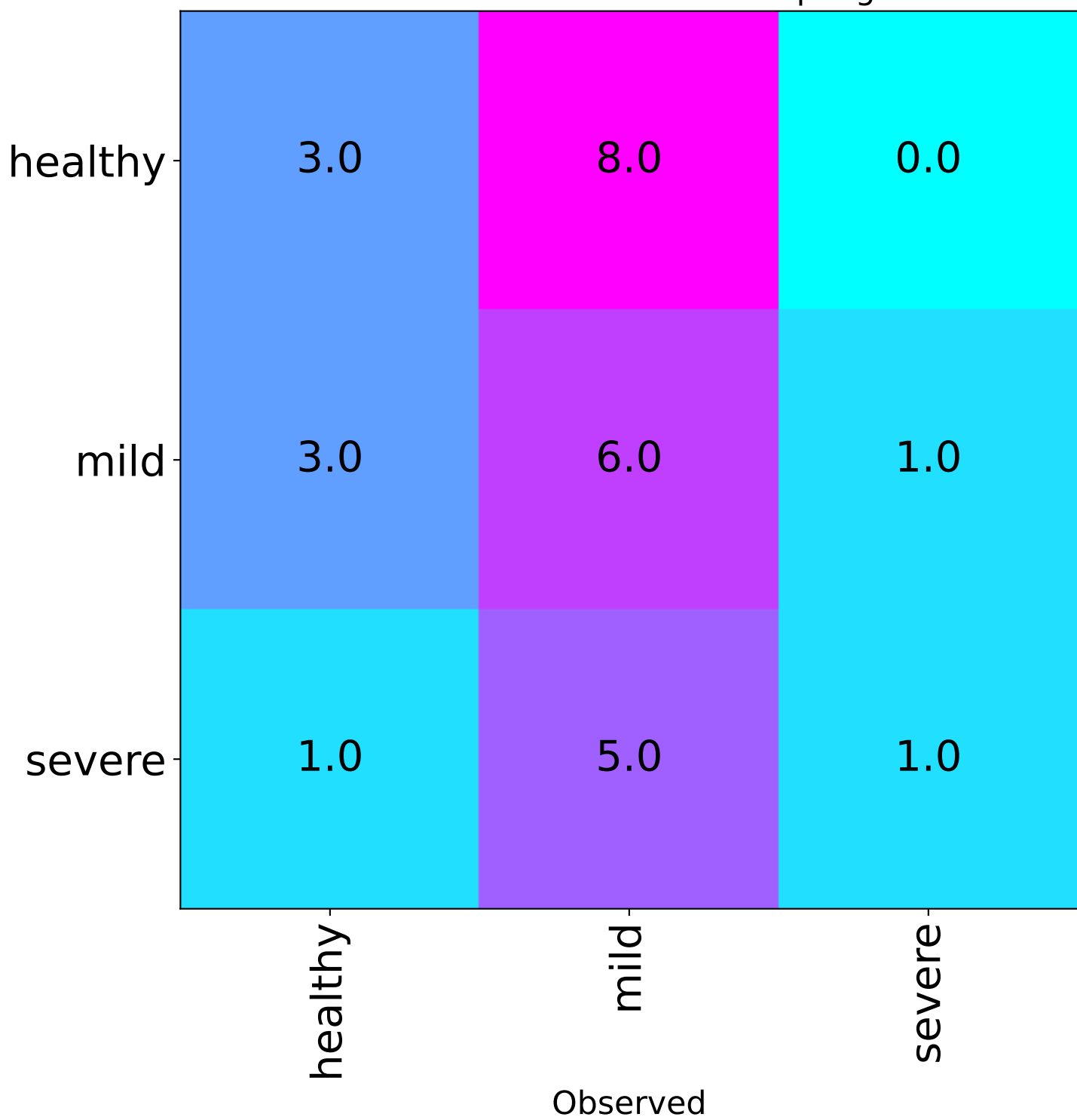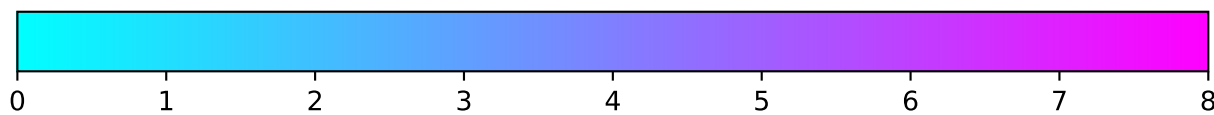

Supplement: Supplementary file 4 — Source Data [file 41467_2023_36140_MOESM4_ESM.zip › Source data/Venet Fig 2b_mDC1s_def/2022-04-28_Thu_16-59-36_GradientBoostClassifier_6/Confusion_matrix_Downsampling 2.pdf]

Confusion matrix Downsampling 3

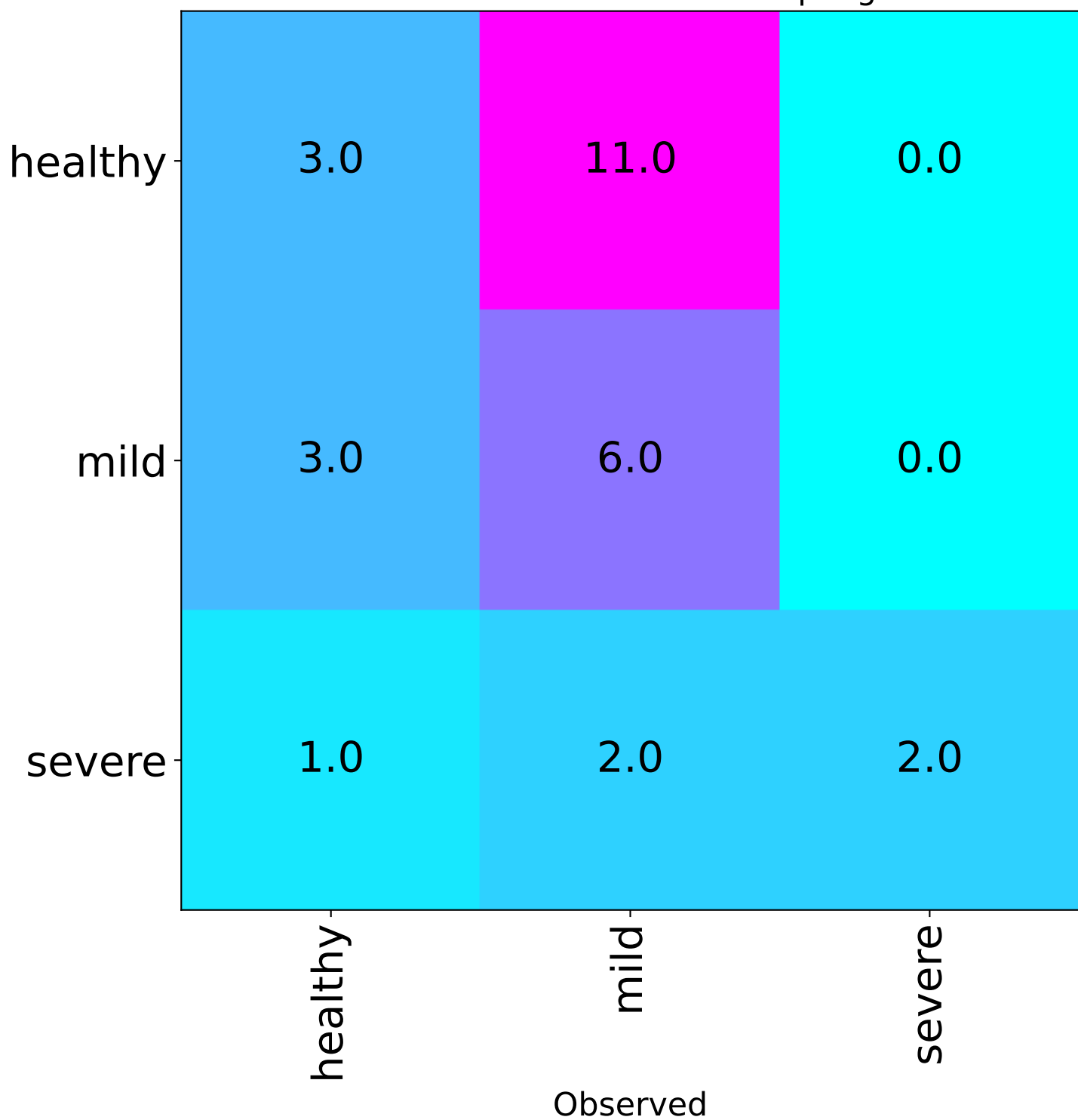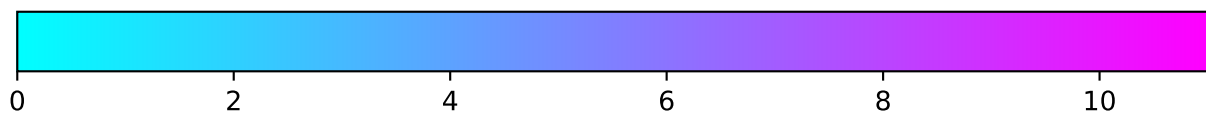

Supplement: Supplementary file 4 — Source Data [file 41467_2023_36140_MOESM4_ESM.zip › Source data/Venet Fig 2b_mDC1s_def/2022-04-28_Thu_16-59-36_GradientBoostClassifier_6/Confusion_matrix_Downsampling 3.pdf]

Confusion matrix Downsampling 8

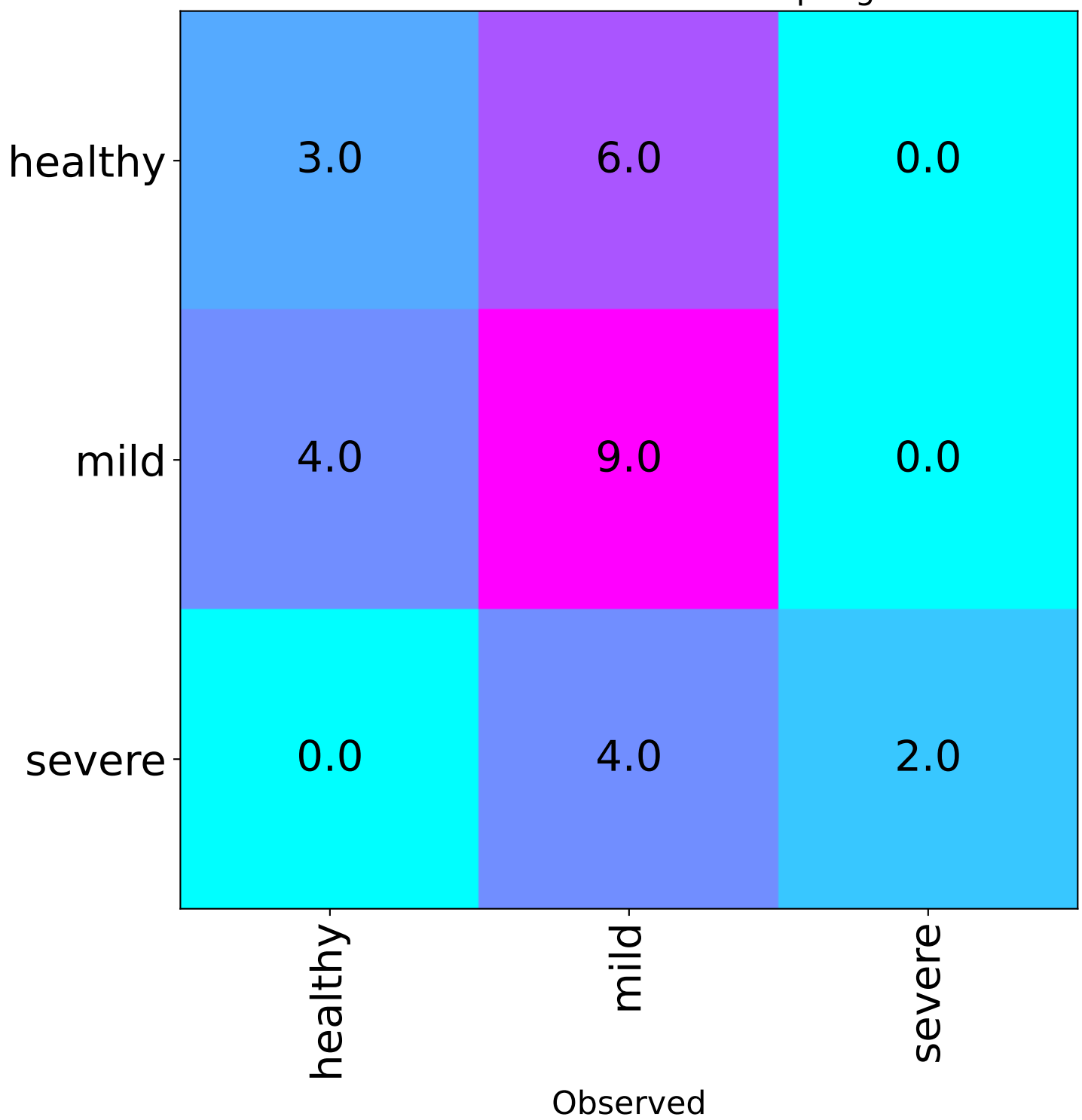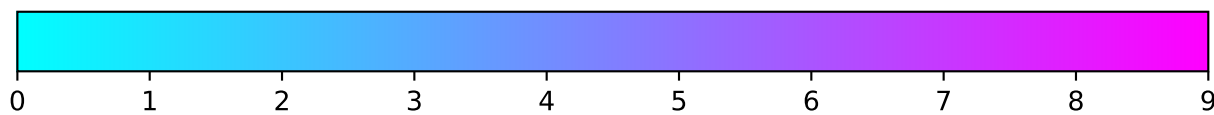

Supplement: Supplementary file 4 — Source Data [file 41467_2023_36140_MOESM4_ESM.zip › Source data/Venet Fig 2b_mDC1s_def/2022-04-28_Thu_16-59-36_GradientBoostClassifier_6/Confusion_matrix_Downsampling 8.pdf]

Confusion matrix Downsampling 9

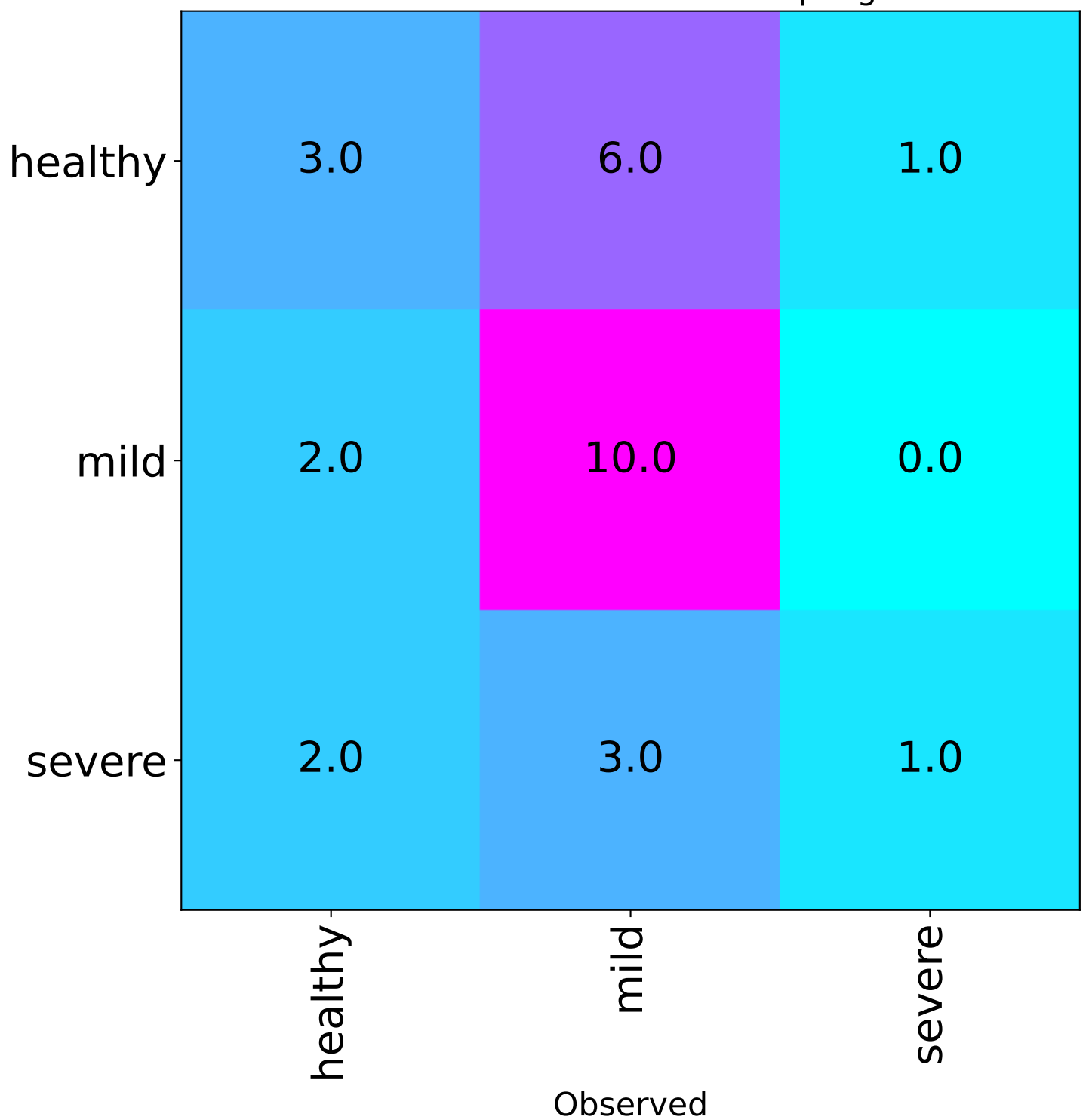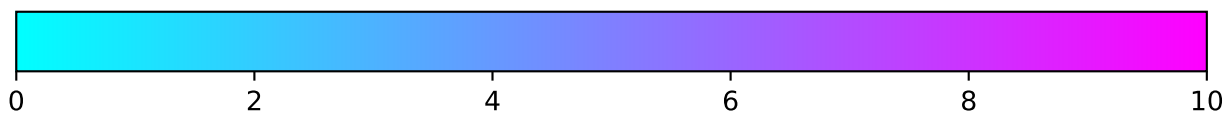

Supplement: Supplementary file 4 — Source Data [file 41467_2023_36140_MOESM4_ESM.zip › Source data/Venet Fig 2b_mDC1s_def/2022-04-28_Thu_16-59-36_GradientBoostClassifier_6/Confusion_matrix_Downsampling 9.pdf]

Confusion matrix Downsampling 7

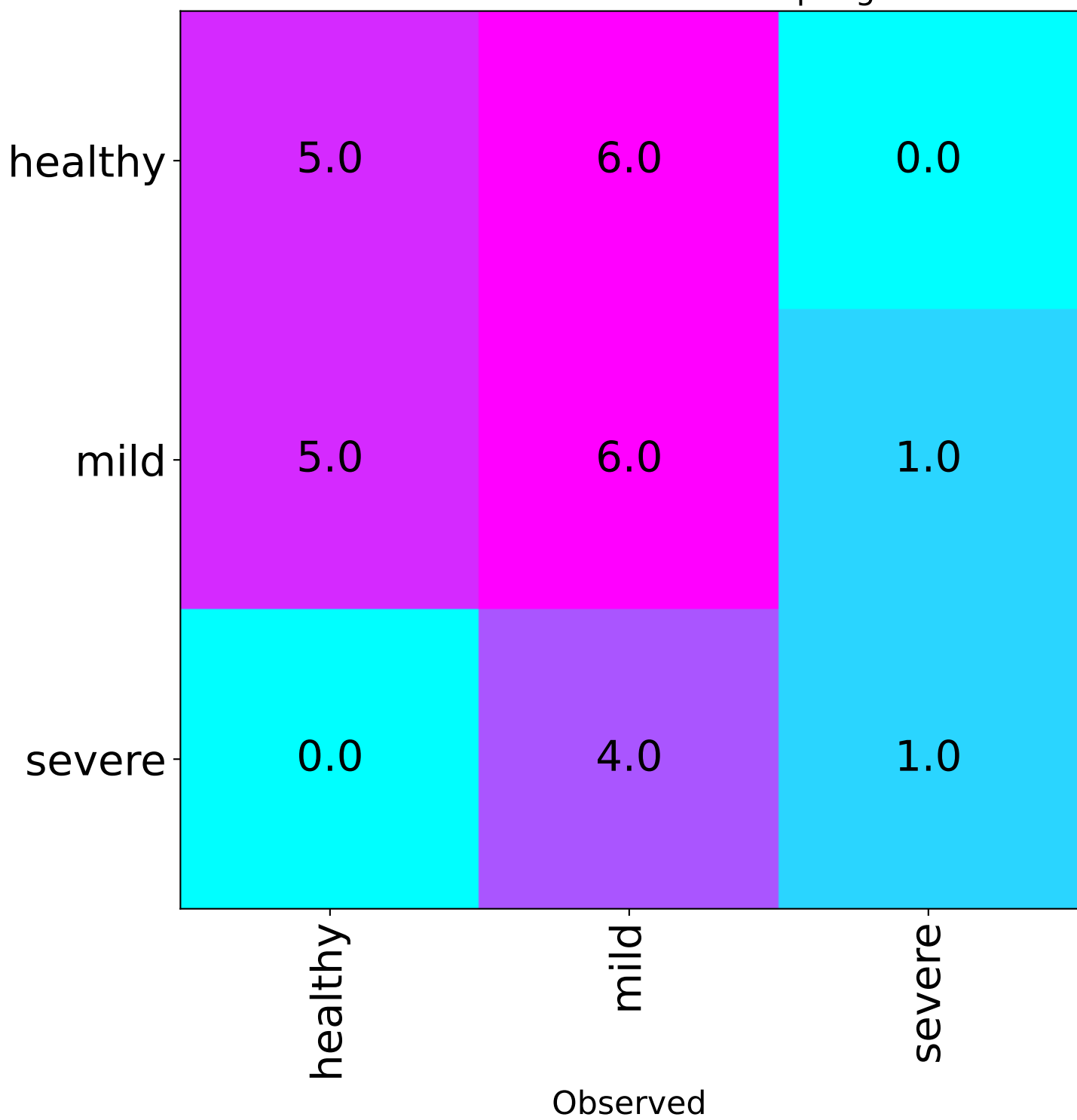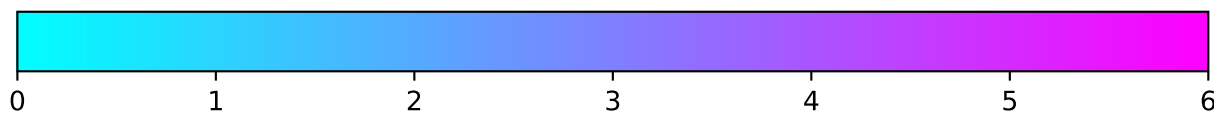

Supplement: Supplementary file 4 — Source Data [file 41467_2023_36140_MOESM4_ESM.zip › Source data/Venet Fig 2b_mDC1s_def/2022-04-28_Thu_16-59-04_GradientBoostClassifier_2/Confusion_matrix_Downsampling 7.pdf]

Confusion matrix Downsampling 6

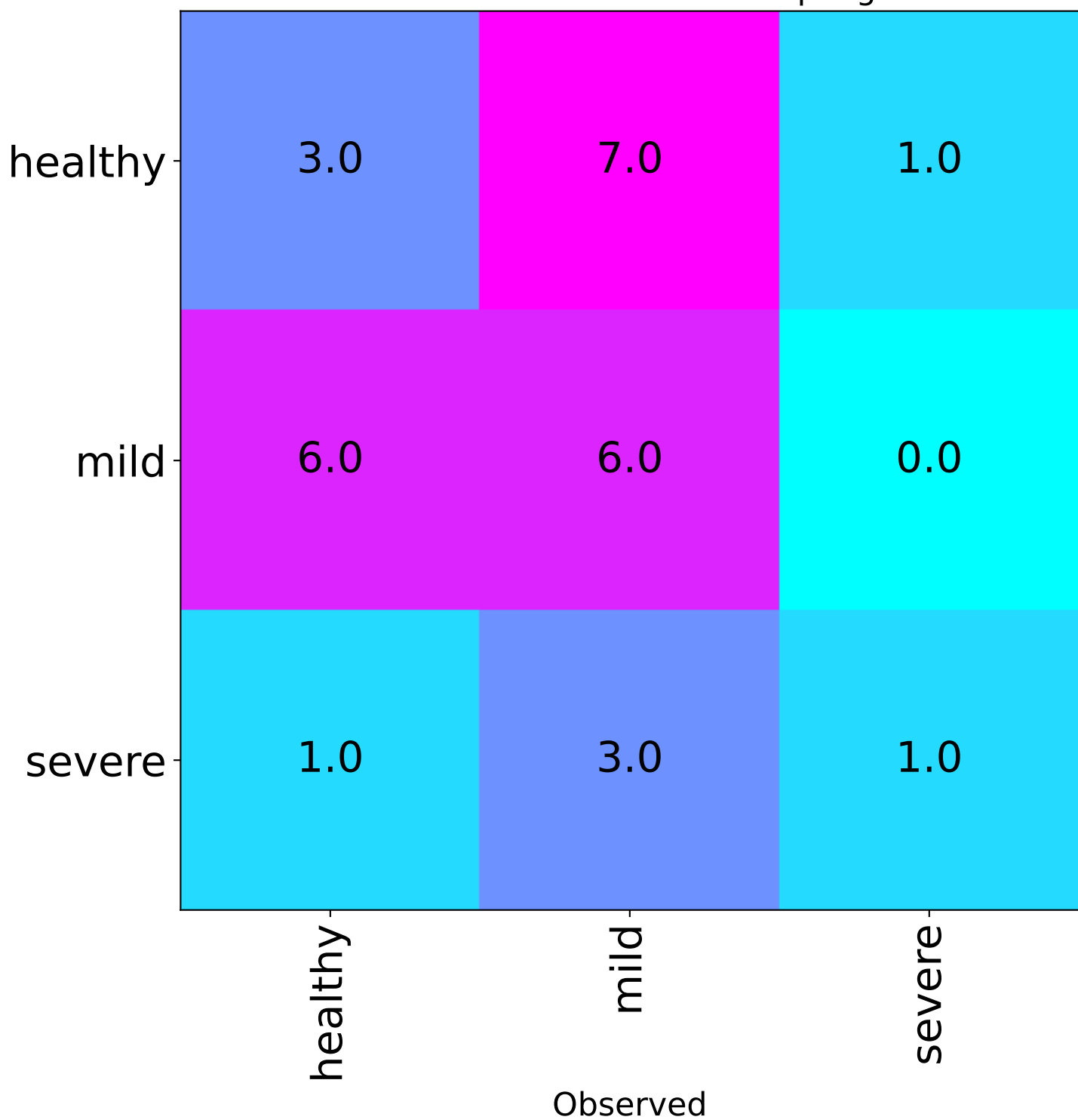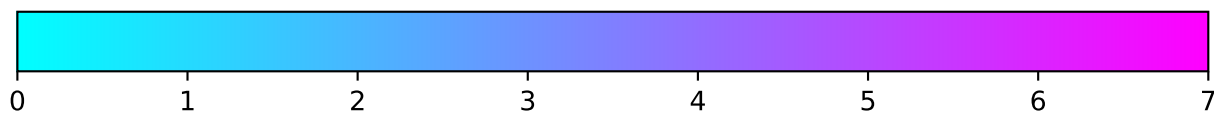

Supplement: Supplementary file 4 — Source Data [file 41467_2023_36140_MOESM4_ESM.zip › Source data/Venet Fig 2b_mDC1s_def/2022-04-28_Thu_16-59-04_GradientBoostClassifier_2/Confusion_matrix_Downsampling 6.pdf]

Confusion matrix Downsampling 4

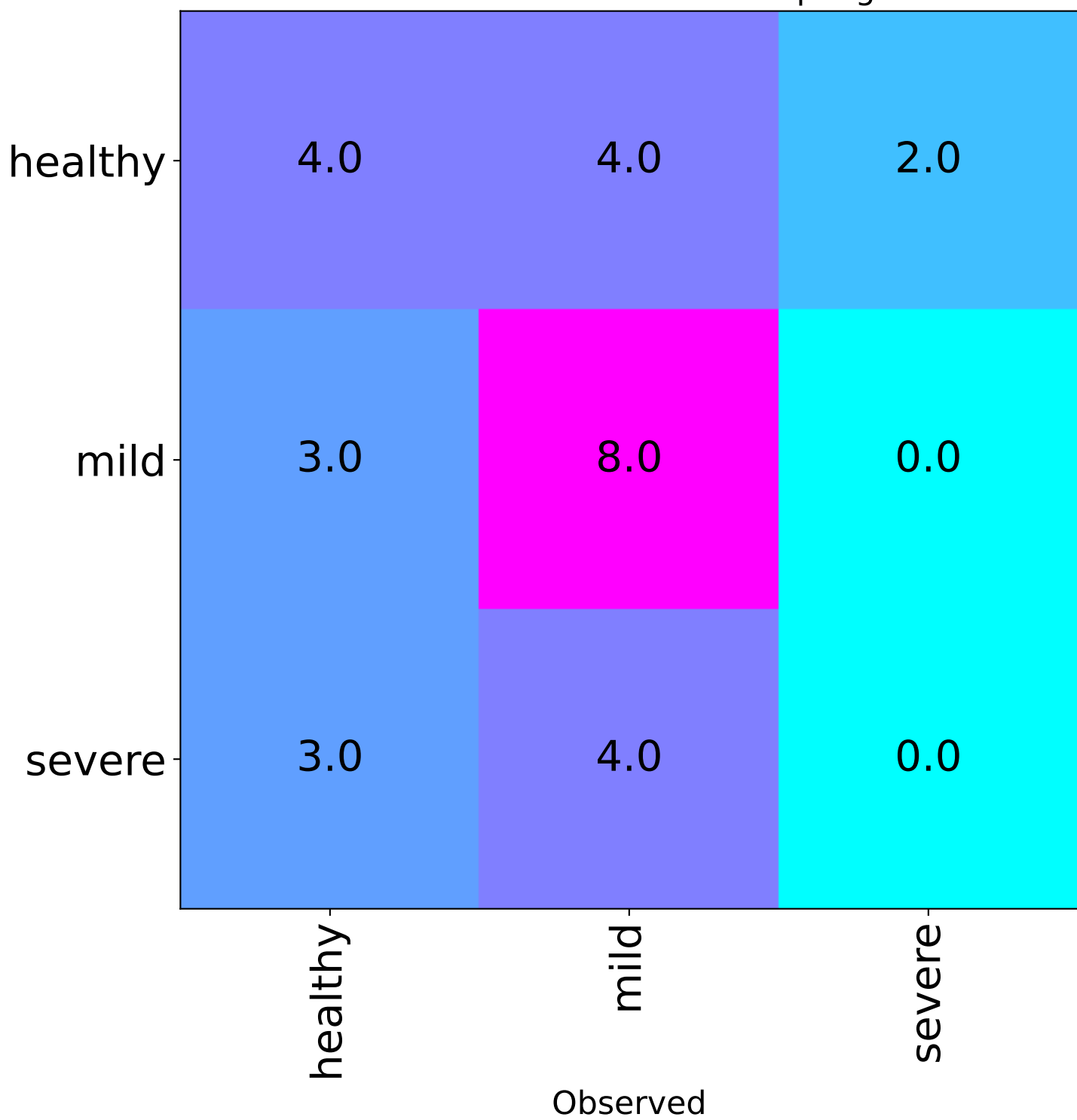

Supplement: Supplementary file 4 — Source Data [file 41467_2023_36140_MOESM4_ESM.zip › Source data/Venet Fig 2b_mDC1s_def/2022-04-28_Thu_16-59-04_GradientBoostClassifier_2/Confusion_matrix_Downsampling 4.pdf]

Confusion matrix Downsampling 5

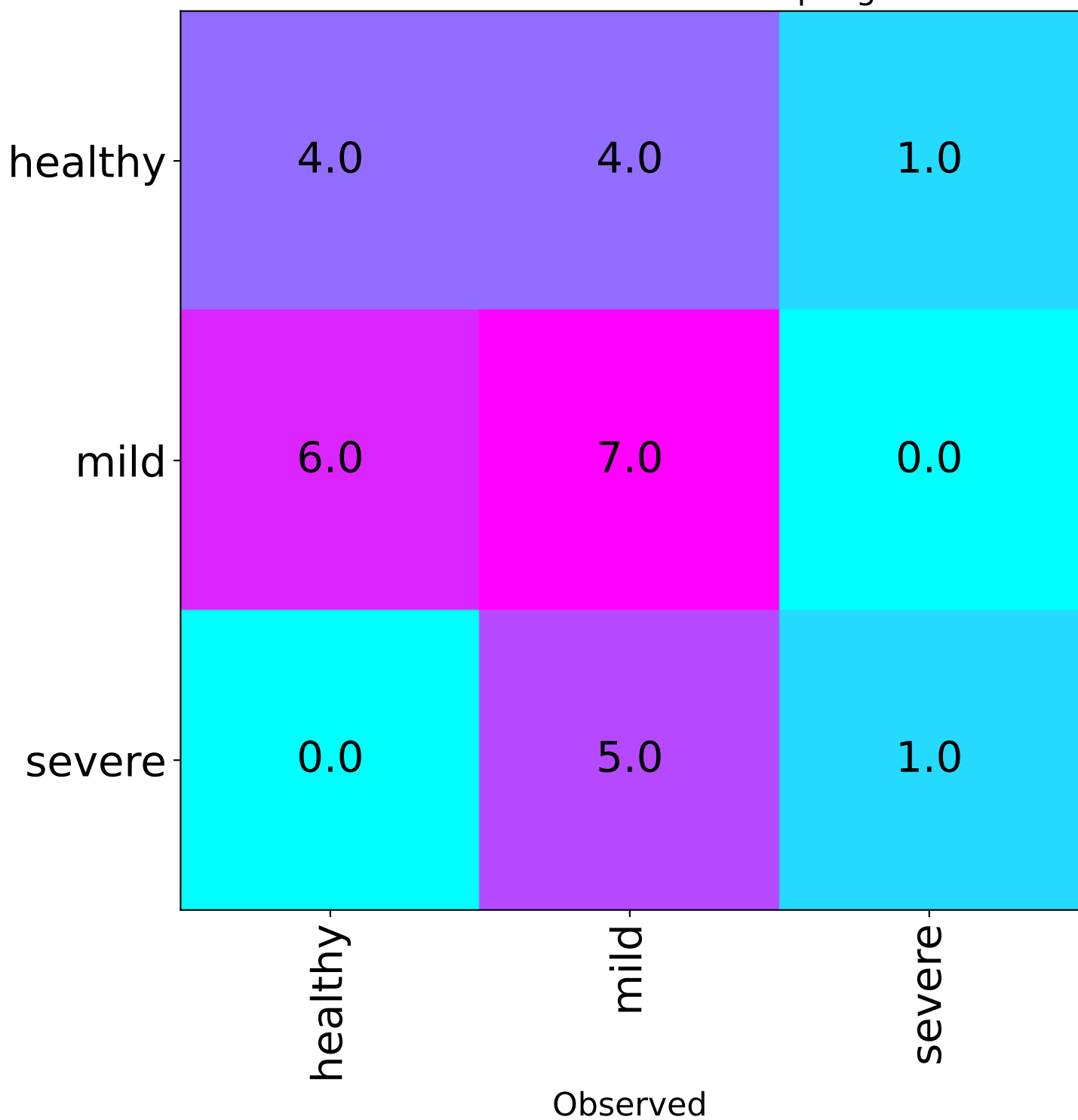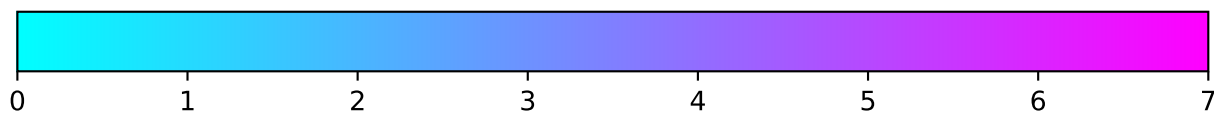

Supplement: Supplementary file 4 — Source Data [file 41467_2023_36140_MOESM4_ESM.zip › Source data/Venet Fig 2b_mDC1s_def/2022-04-28_Thu_16-59-04_GradientBoostClassifier_2/Confusion_matrix_Downsampling 5.pdf]

Confusion matrix Downsampling 1

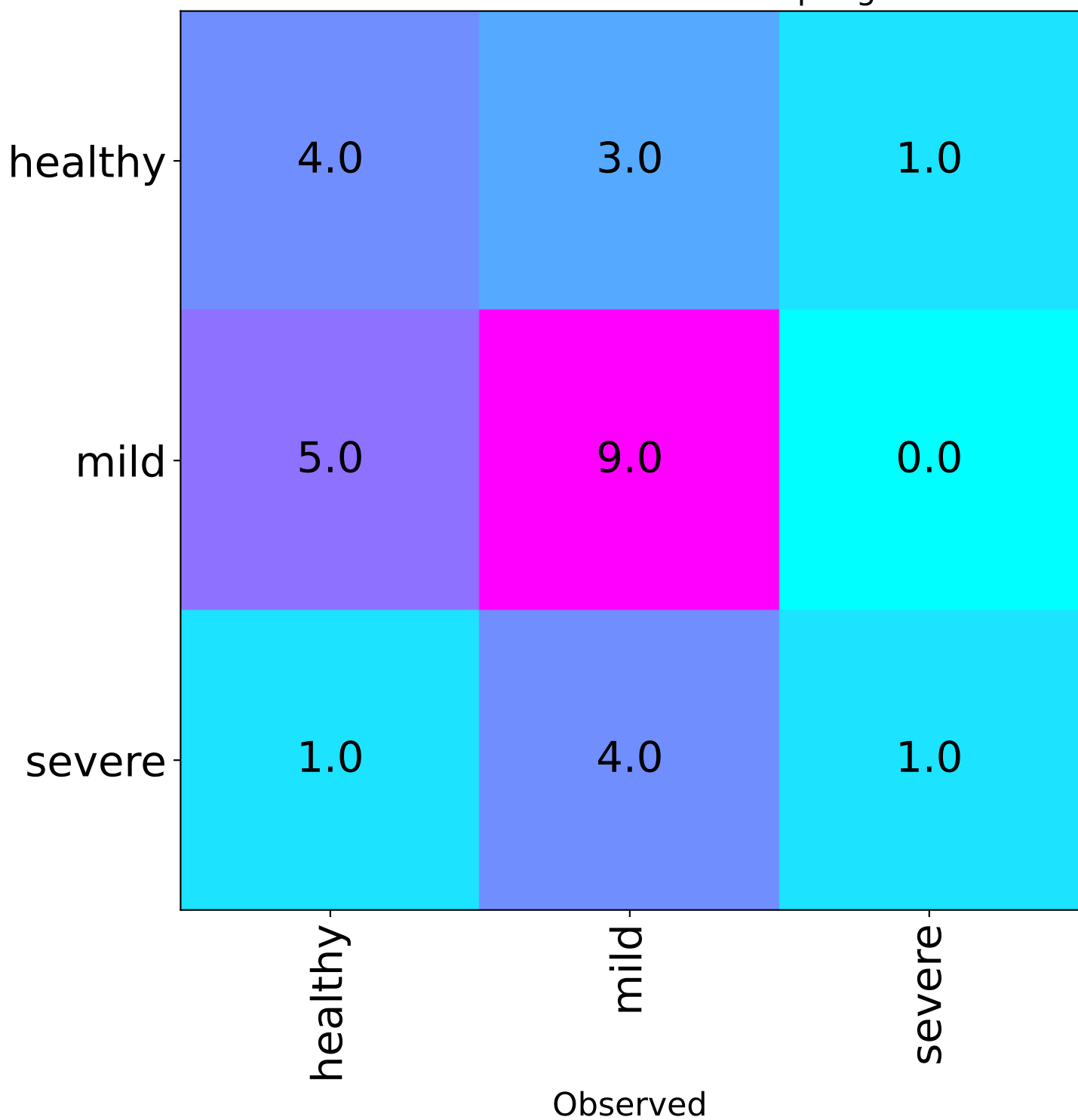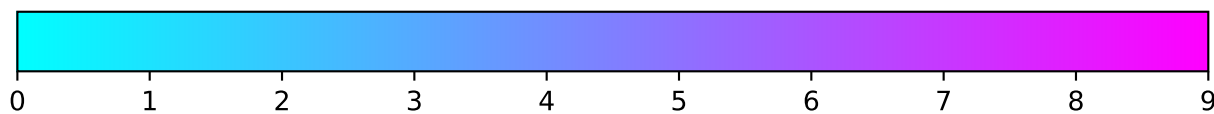

Supplement: Supplementary file 4 — Source Data [file 41467_2023_36140_MOESM4_ESM.zip › Source data/Venet Fig 2b_mDC1s_def/2022-04-28_Thu_16-59-04_GradientBoostClassifier_2/Confusion_matrix_Downsampling 1.pdf]

Confusion matrix Downsampling 0

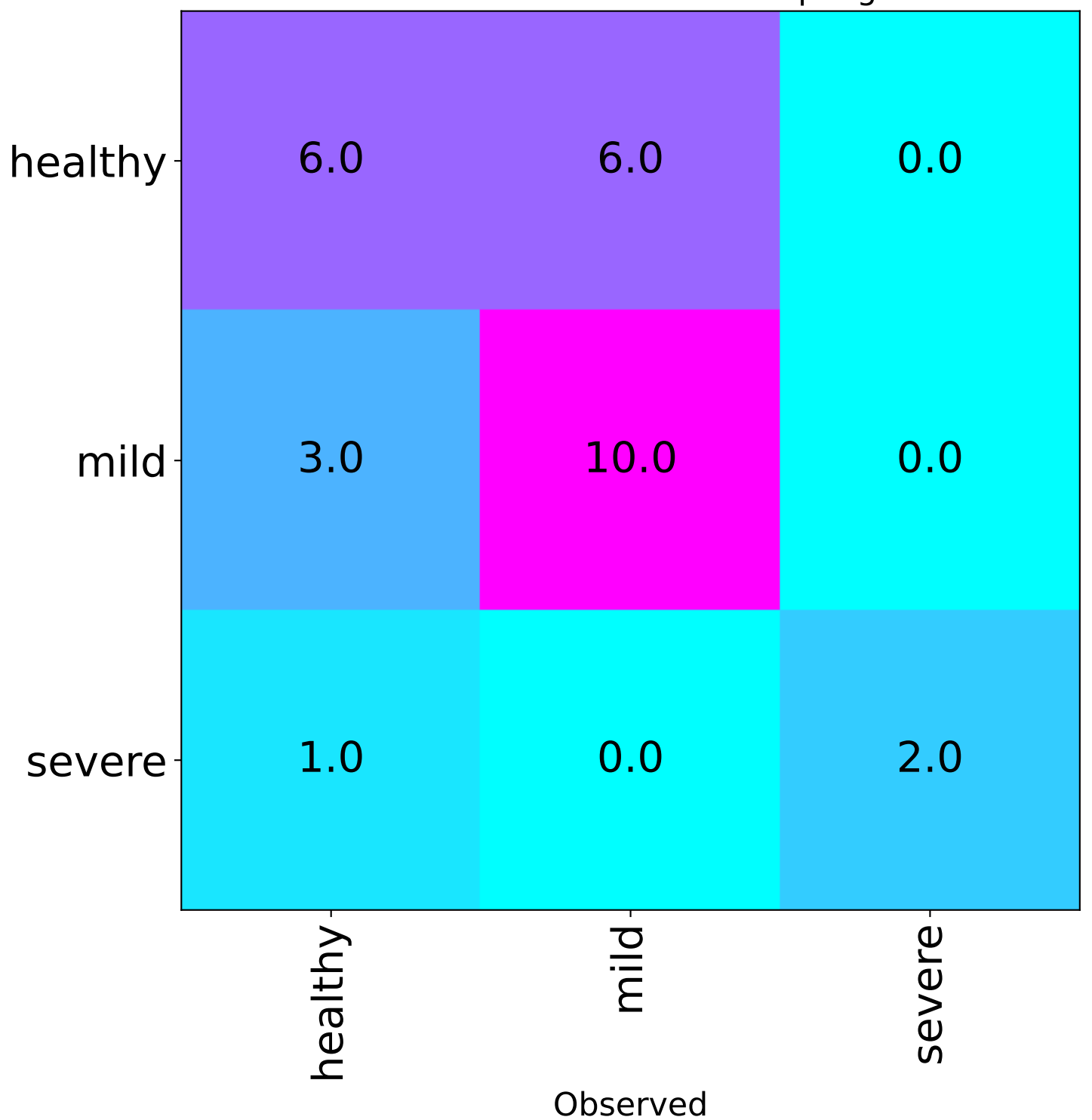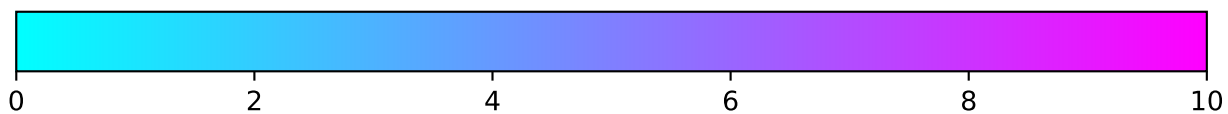

Supplement: Supplementary file 4 — Source Data [file 41467_2023_36140_MOESM4_ESM.zip › Source data/Venet Fig 2b_mDC1s_def/2022-04-28_Thu_16-59-04_GradientBoostClassifier_2/Confusion_matrix_Downsampling 0.pdf]

Confusion matrix Downsampling 2

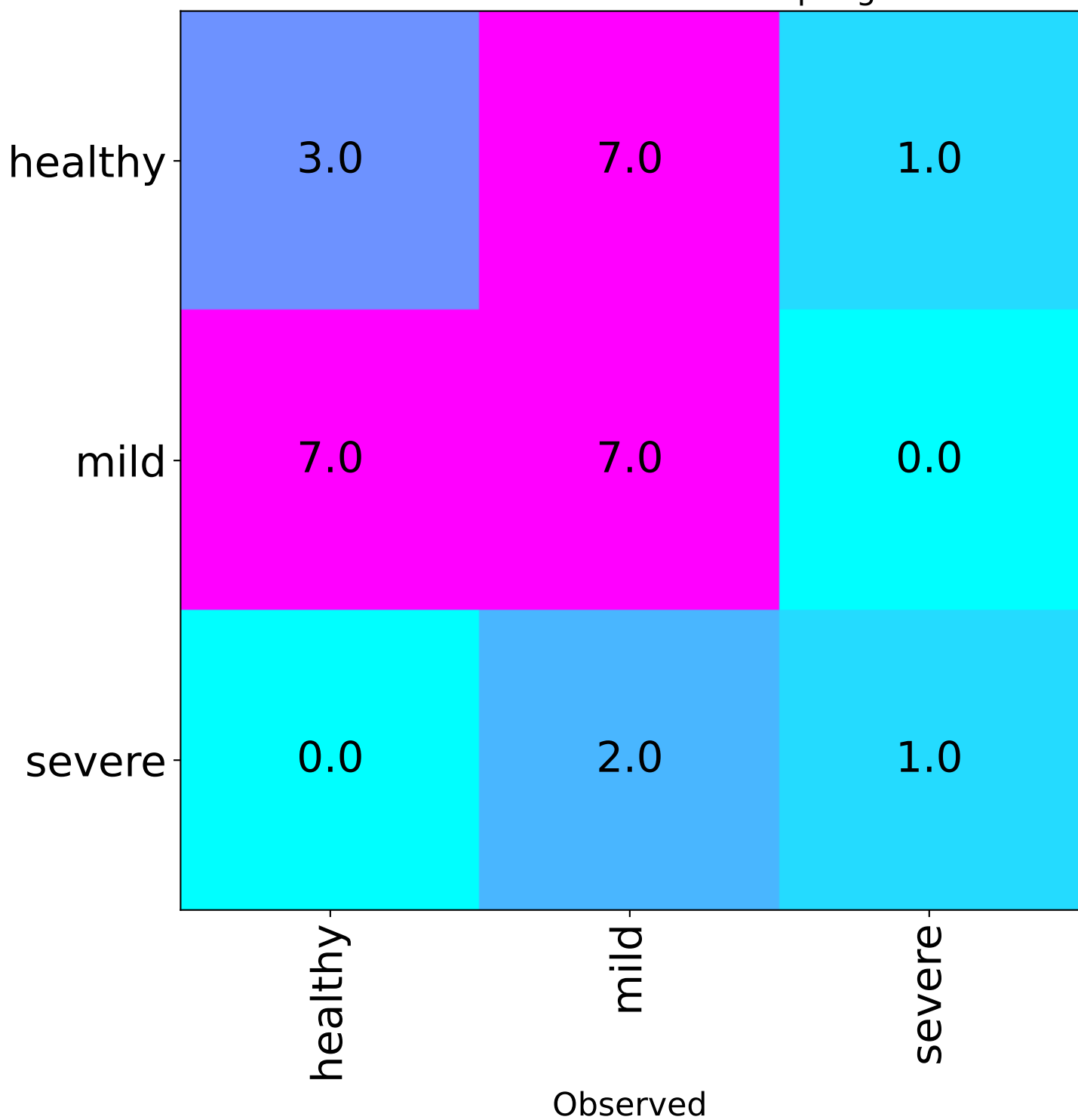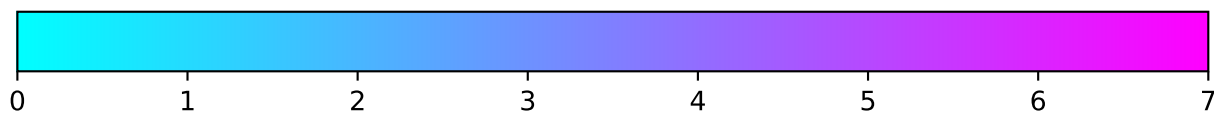

Supplement: Supplementary file 4 — Source Data [file 41467_2023_36140_MOESM4_ESM.zip › Source data/Venet Fig 2b_mDC1s_def/2022-04-28_Thu_16-59-04_GradientBoostClassifier_2/Confusion_matrix_Downsampling 2.pdf]

Confusion matrix Downsampling 3

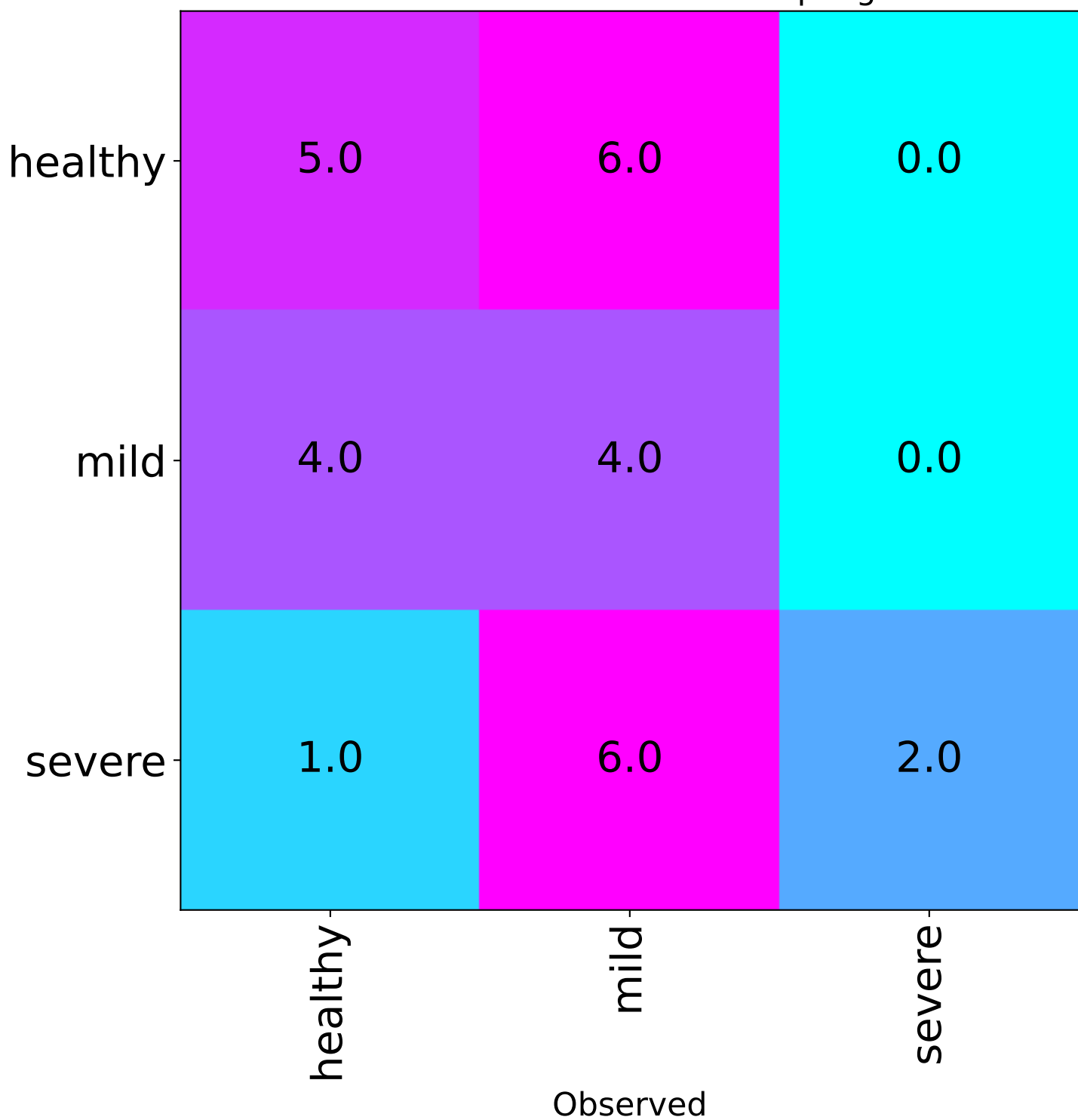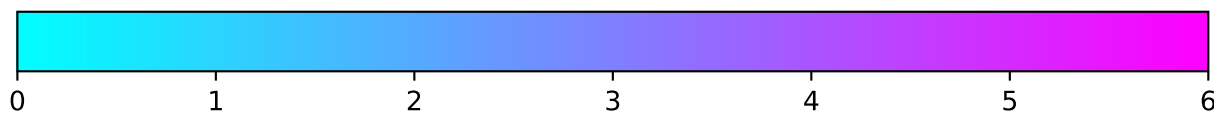

Supplement: Supplementary file 4 — Source Data [file 41467_2023_36140_MOESM4_ESM.zip › Source data/Venet Fig 2b_mDC1s_def/2022-04-28_Thu_16-59-04_GradientBoostClassifier_2/Confusion_matrix_Downsampling 3.pdf]

Confusion matrix Downsampling 9

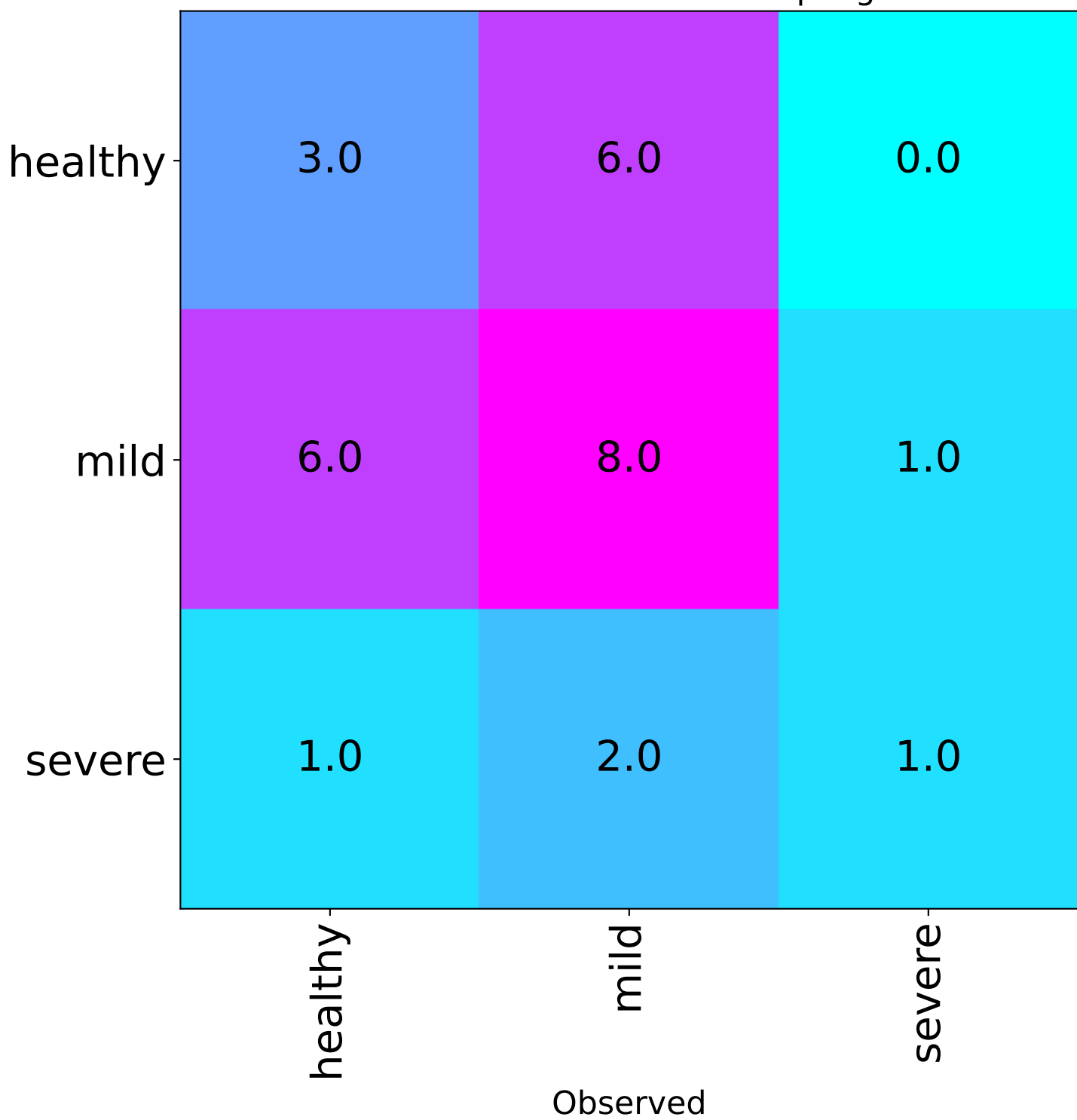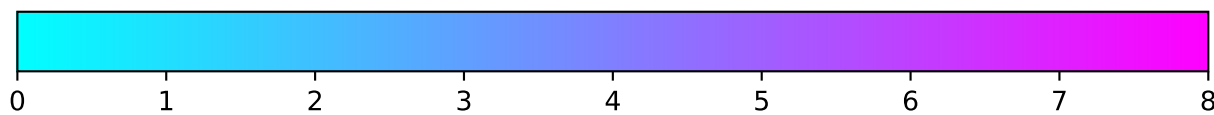

Supplement: Supplementary file 4 — Source Data [file 41467_2023_36140_MOESM4_ESM.zip › Source data/Venet Fig 2b_mDC1s_def/2022-04-28_Thu_16-59-04_GradientBoostClassifier_2/Confusion_matrix_Downsampling 9.pdf]

Confusion matrix Downsampling 7

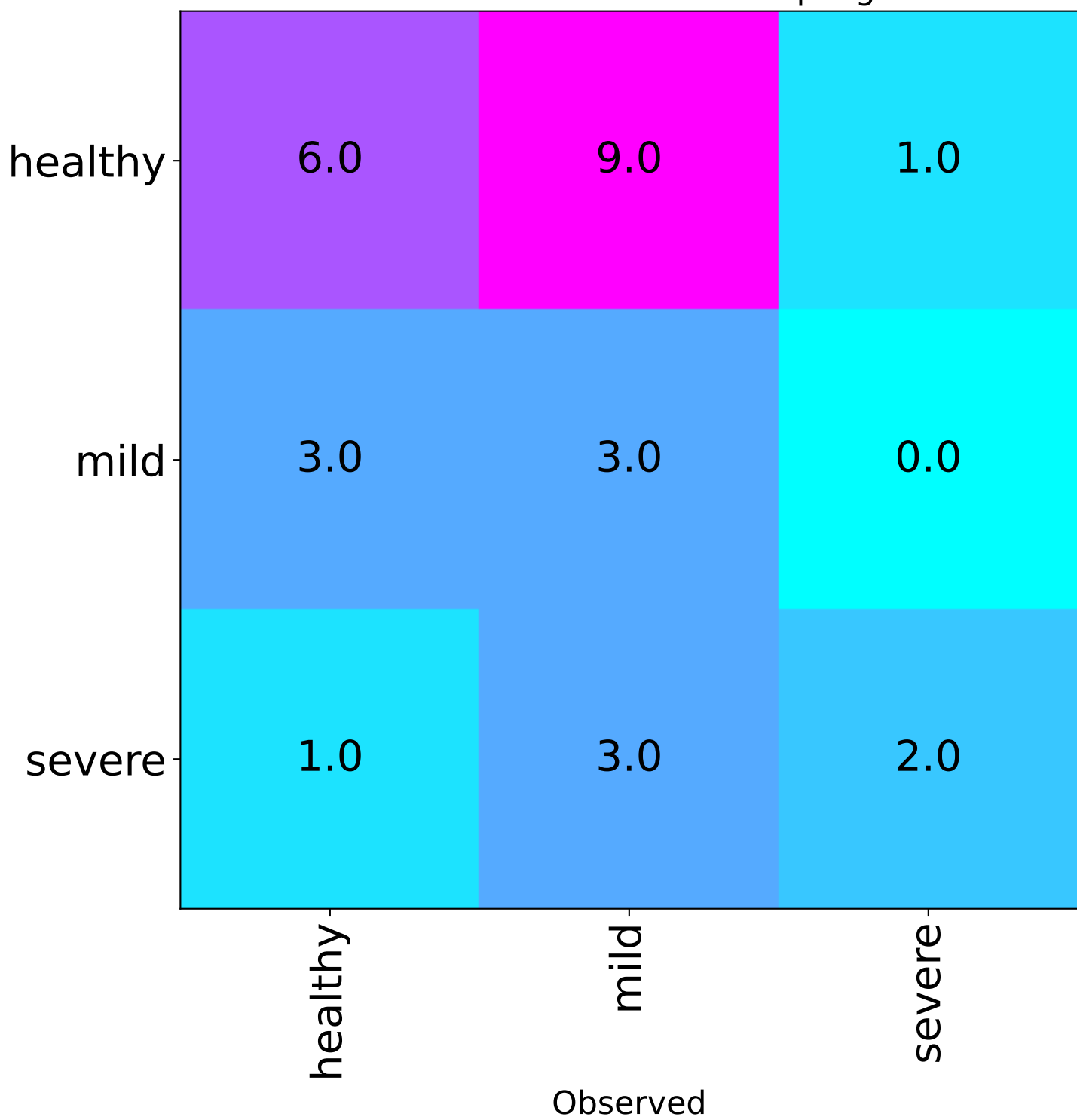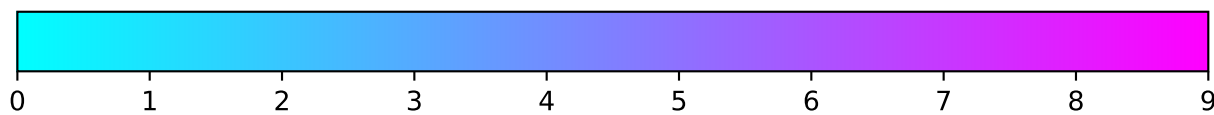

Supplement: Supplementary file 4 — Source Data [file 41467_2023_36140_MOESM4_ESM.zip › Source data/Venet Fig 2b_mDC1s_def/2022-04-28_Thu_16-59-44_GradientBoostClassifier_7/Confusion_matrix_Downsampling 7.pdf]

Confusion matrix Downsampling 6

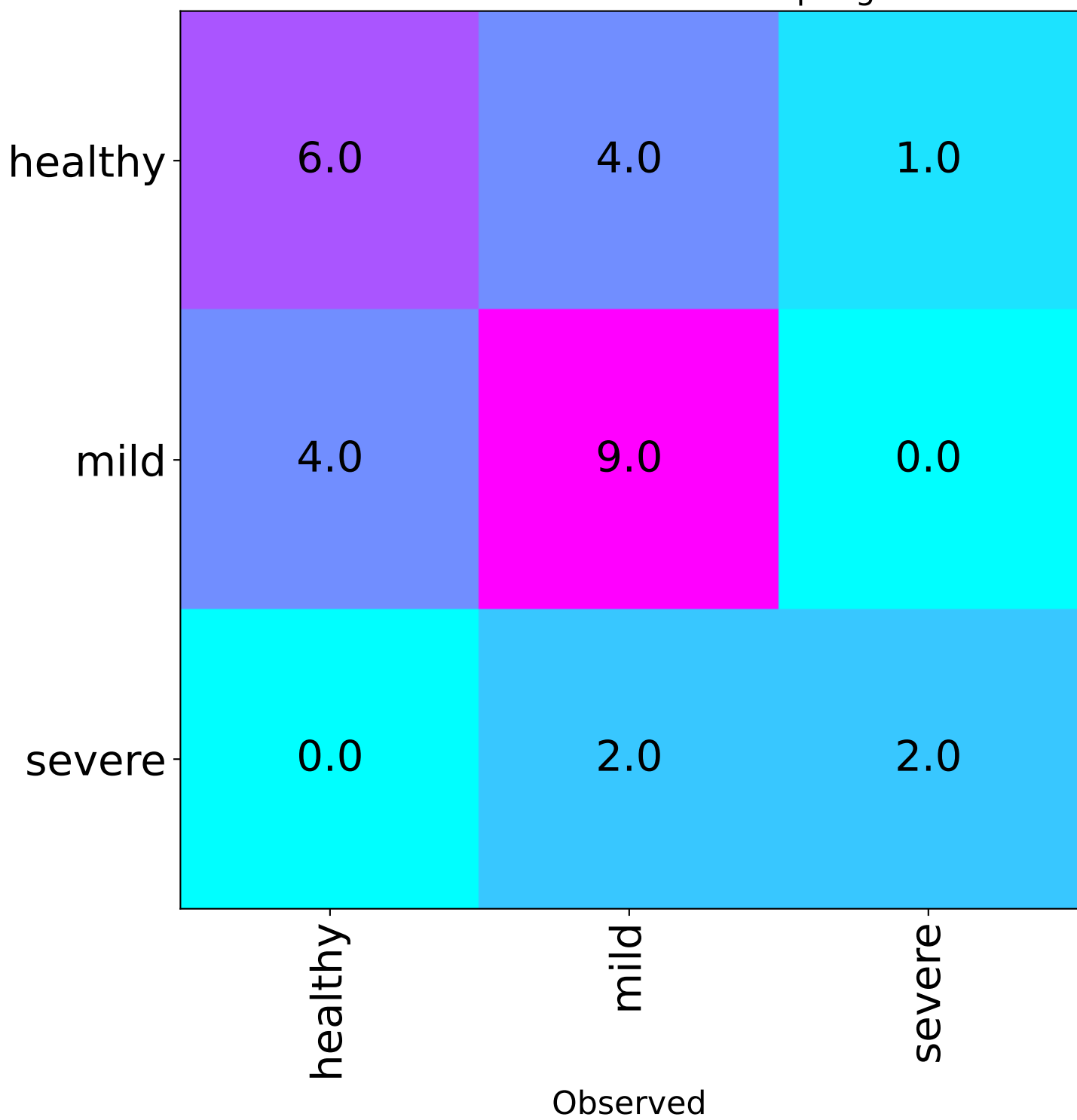

Supplement: Supplementary file 4 — Source Data [file 41467_2023_36140_MOESM4_ESM.zip › Source data/Venet Fig 2b_mDC1s_def/2022-04-28_Thu_16-59-44_GradientBoostClassifier_7/Confusion_matrix_Downsampling 6.pdf]

Confusion matrix Downsampling 4

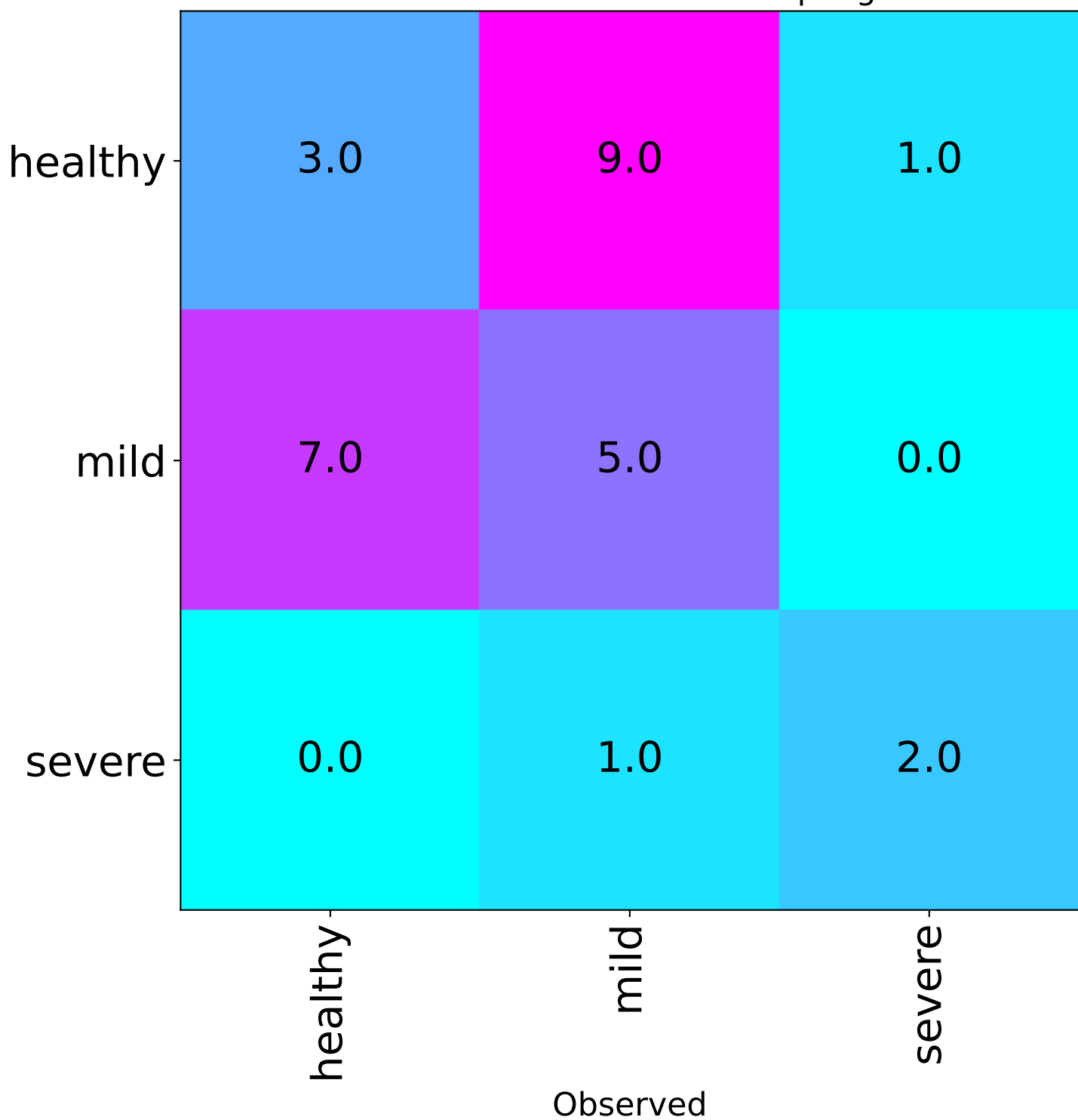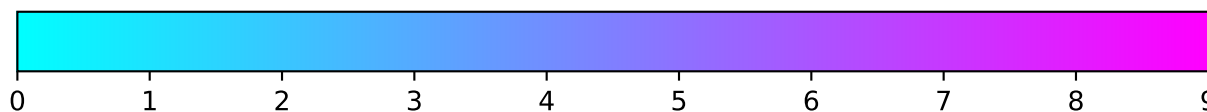

Supplement: Supplementary file 4 — Source Data [file 41467_2023_36140_MOESM4_ESM.zip › Source data/Venet Fig 2b_mDC1s_def/2022-04-28_Thu_16-59-44_GradientBoostClassifier_7/Confusion_matrix_Downsampling 4.pdf]

Confusion matrix Downsampling 5

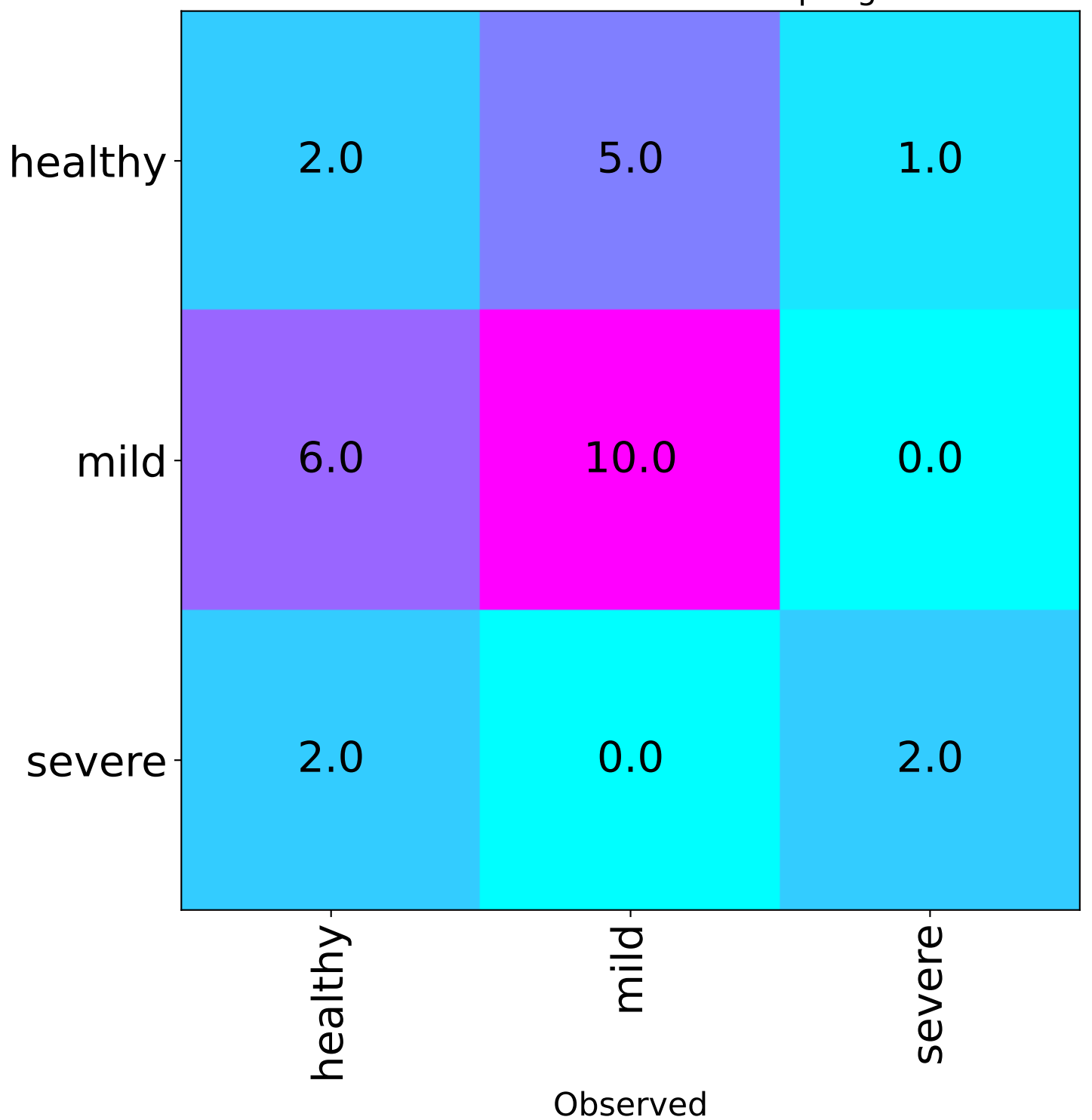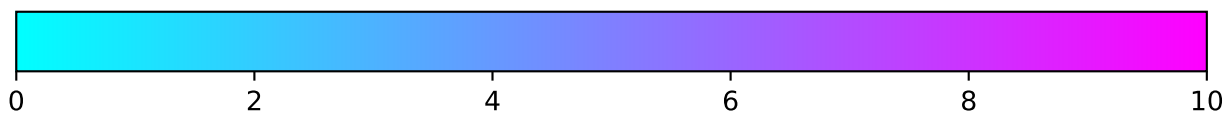

Supplement: Supplementary file 4 — Source Data [file 41467_2023_36140_MOESM4_ESM.zip › Source data/Venet Fig 2b_mDC1s_def/2022-04-28_Thu_16-59-44_GradientBoostClassifier_7/Confusion_matrix_Downsampling 5.pdf]

Confusion matrix Downsampling 1

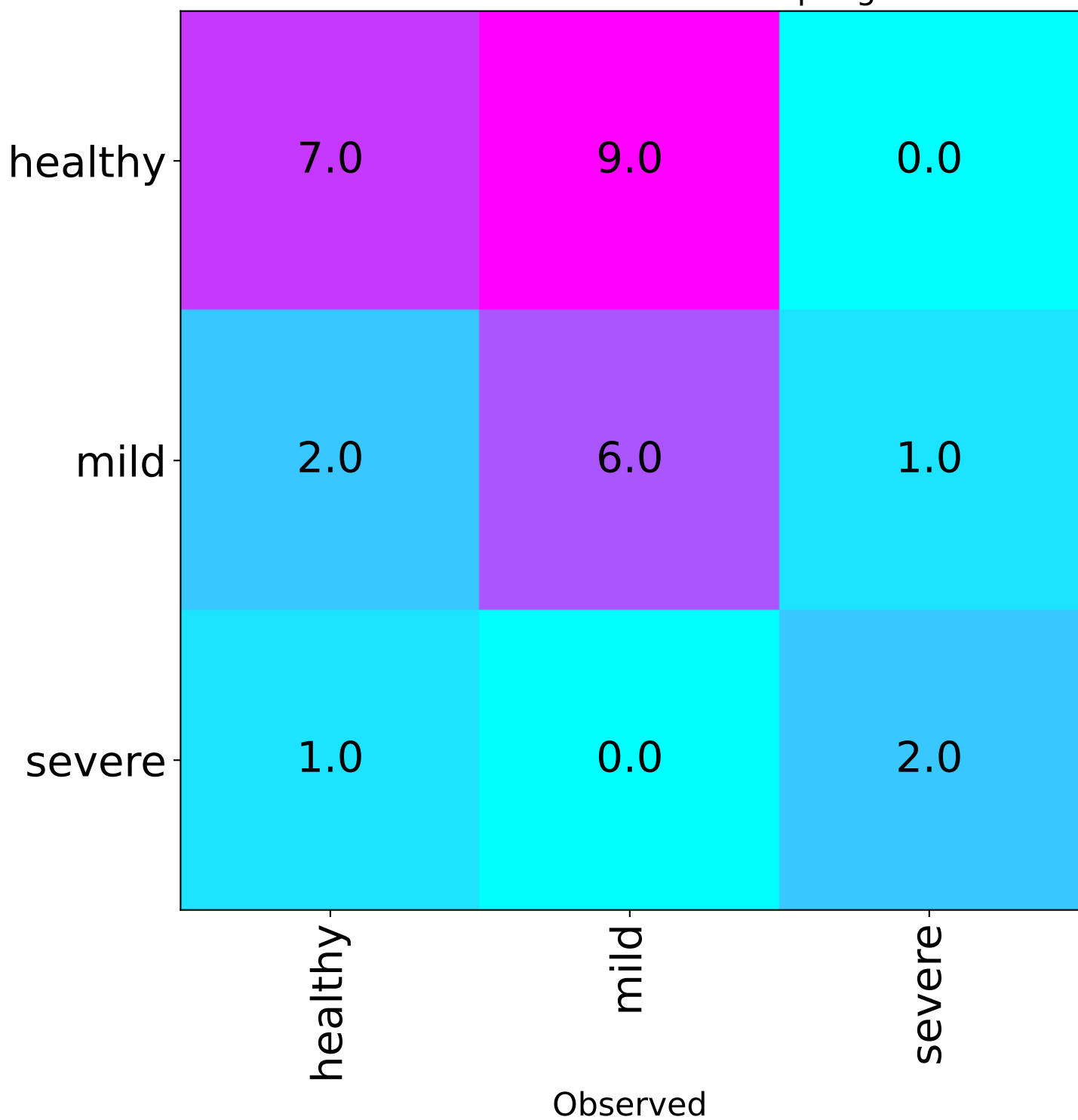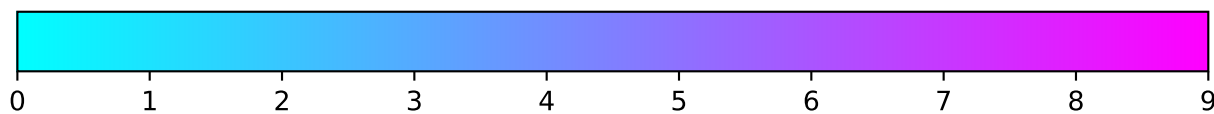

Supplement: Supplementary file 4 — Source Data [file 41467_2023_36140_MOESM4_ESM.zip › Source data/Venet Fig 2b_mDC1s_def/2022-04-28_Thu_16-59-44_GradientBoostClassifier_7/Confusion_matrix_Downsampling 1.pdf]

Confusion matrix Downsampling 0

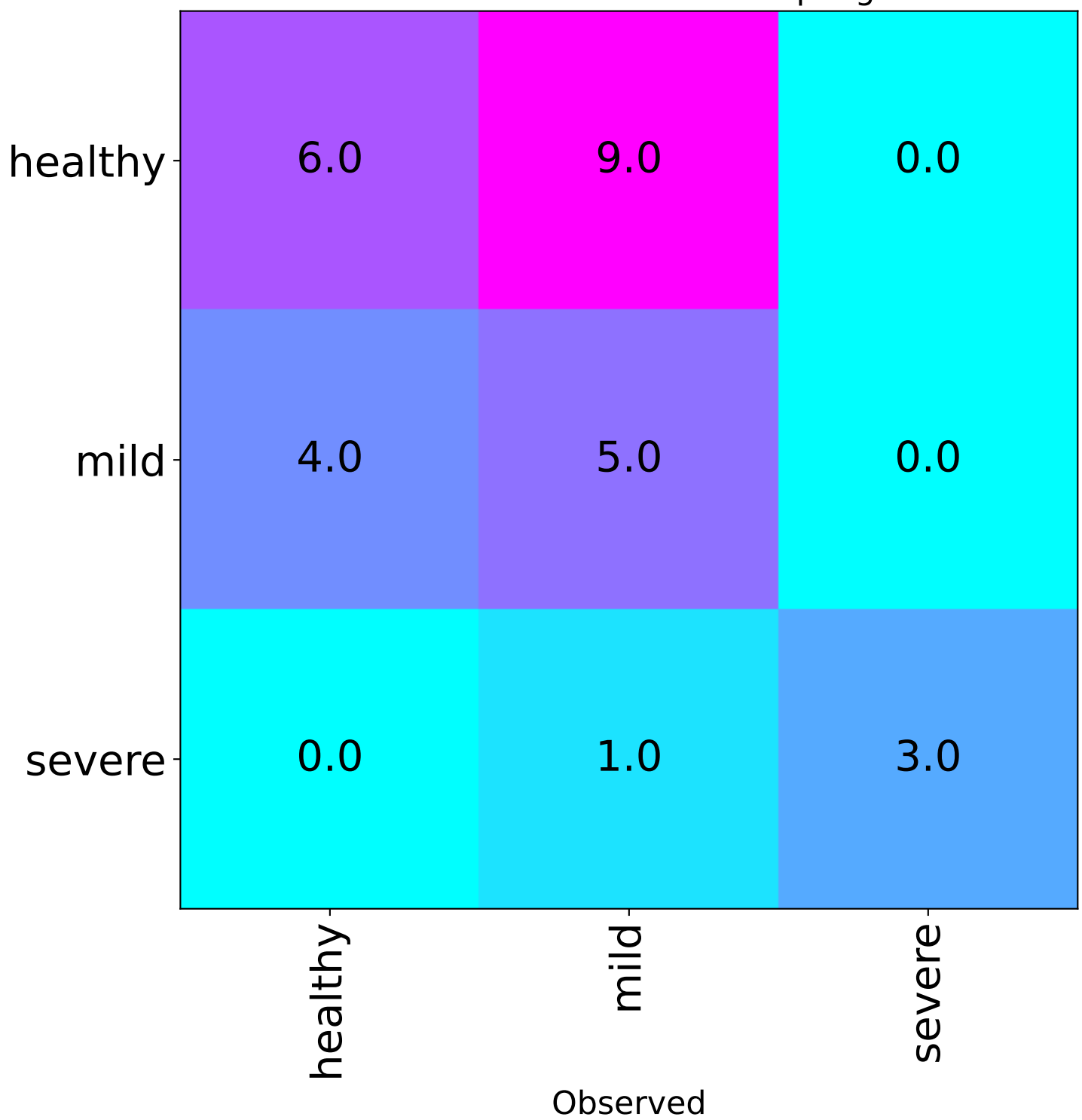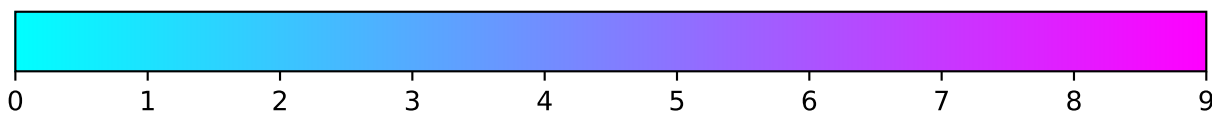

Supplement: Supplementary file 4 — Source Data [file 41467_2023_36140_MOESM4_ESM.zip › Source data/Venet Fig 2b_mDC1s_def/2022-04-28_Thu_16-59-44_GradientBoostClassifier_7/Confusion_matrix_Downsampling 0.pdf]

Confusion matrix Downsampling 2

Predicted

healthy

4.0

7.0

1.0

mild

3.0

6.0

0.0

severe

3.0

2.0

2.0

healthy

mild

severe

Observed

0 1 2 3 4 5 6 7

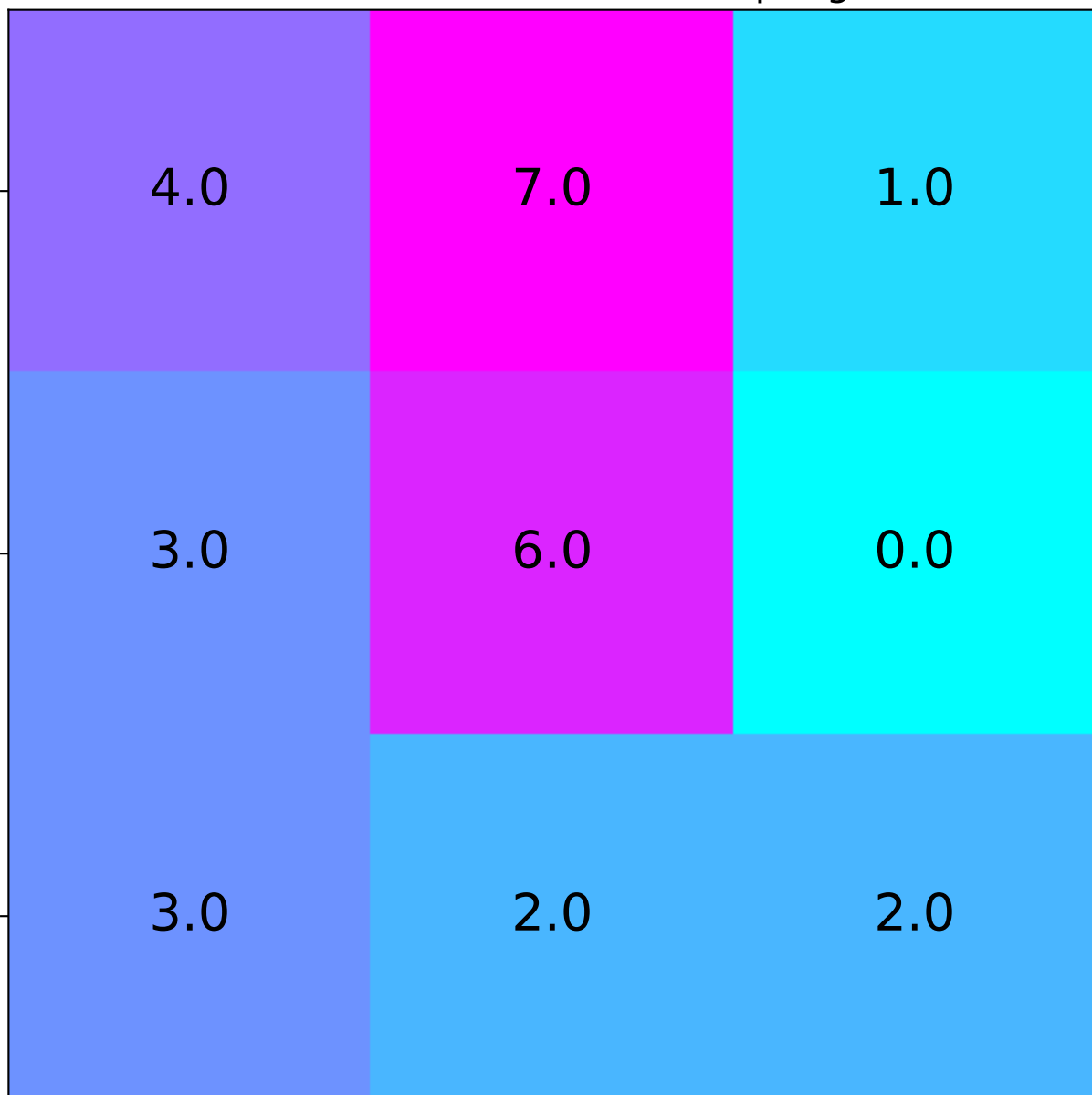

Supplement: Supplementary file 4 — Source Data [file 41467_2023_36140_MOESM4_ESM.zip › Source data/Venet Fig 2b_mDC1s_def/2022-04-28_Thu_16-59-44_GradientBoostClassifier_7/Confusion_matrix_Downsampling 2.pdf]

Confusion matrix Downsampling 8

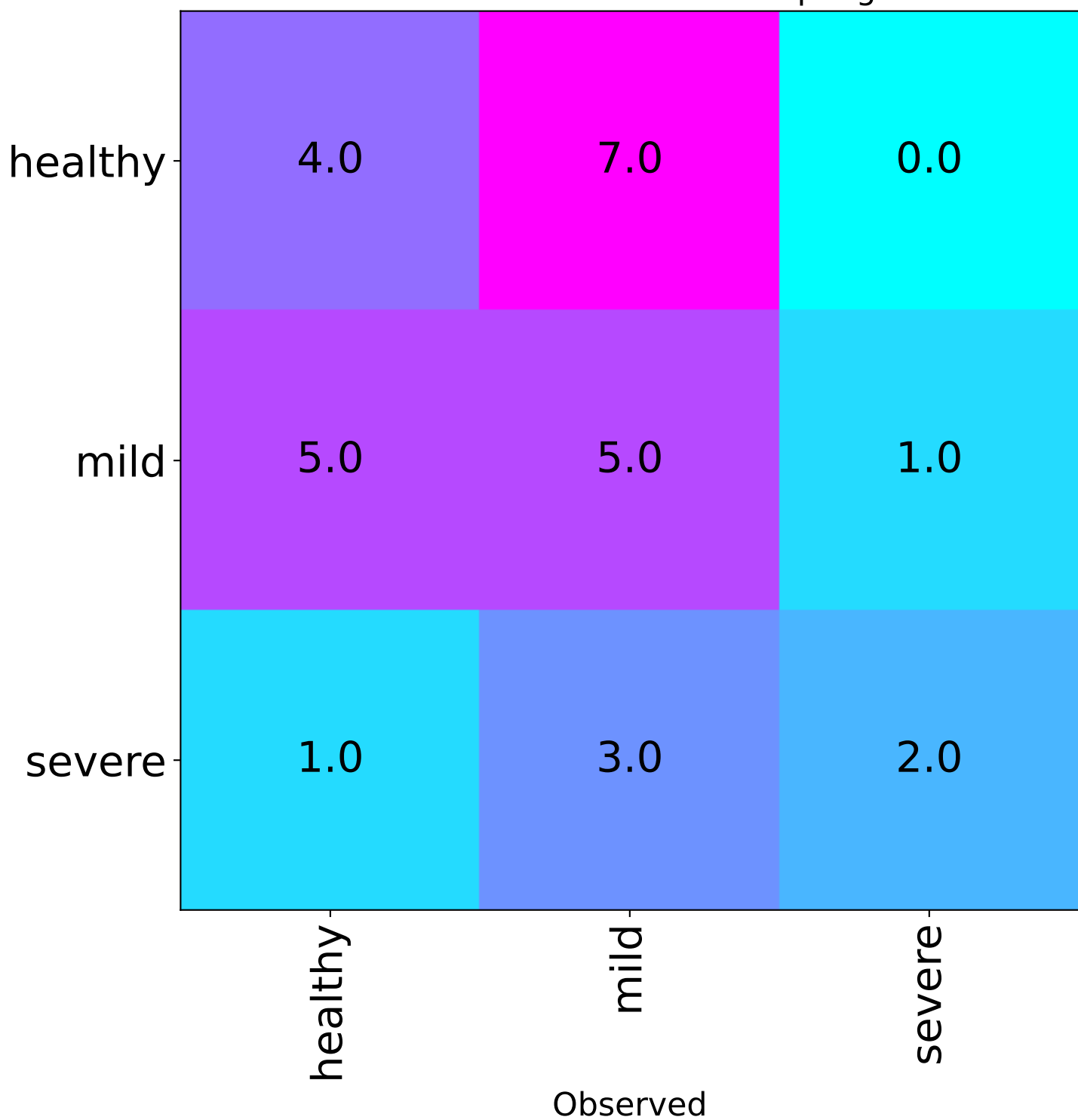

Supplement: Supplementary file 4 — Source Data [file 41467_2023_36140_MOESM4_ESM.zip › Source data/Venet Fig 2b_mDC1s_def/2022-04-28_Thu_16-59-44_GradientBoostClassifier_7/Confusion_matrix_Downsampling 8.pdf]

Confusion matrix Downsampling 9

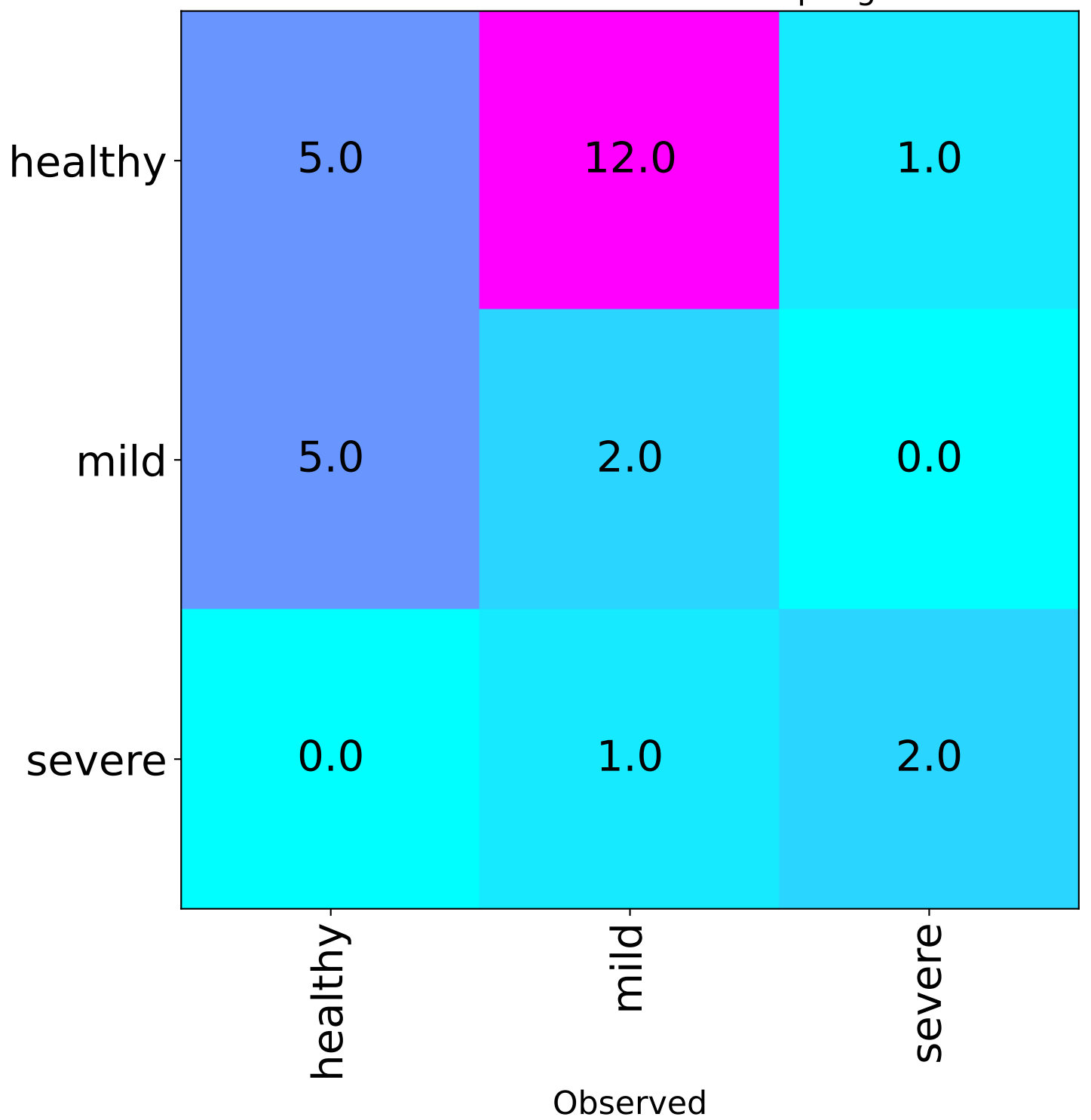

Supplement: Supplementary file 4 — Source Data [file 41467_2023_36140_MOESM4_ESM.zip › Source data/Venet Fig 2b_mDC1s_def/2022-04-28_Thu_16-59-44_GradientBoostClassifier_7/Confusion_matrix_Downsampling 9.pdf]

Confusion matrix Downsampling 7

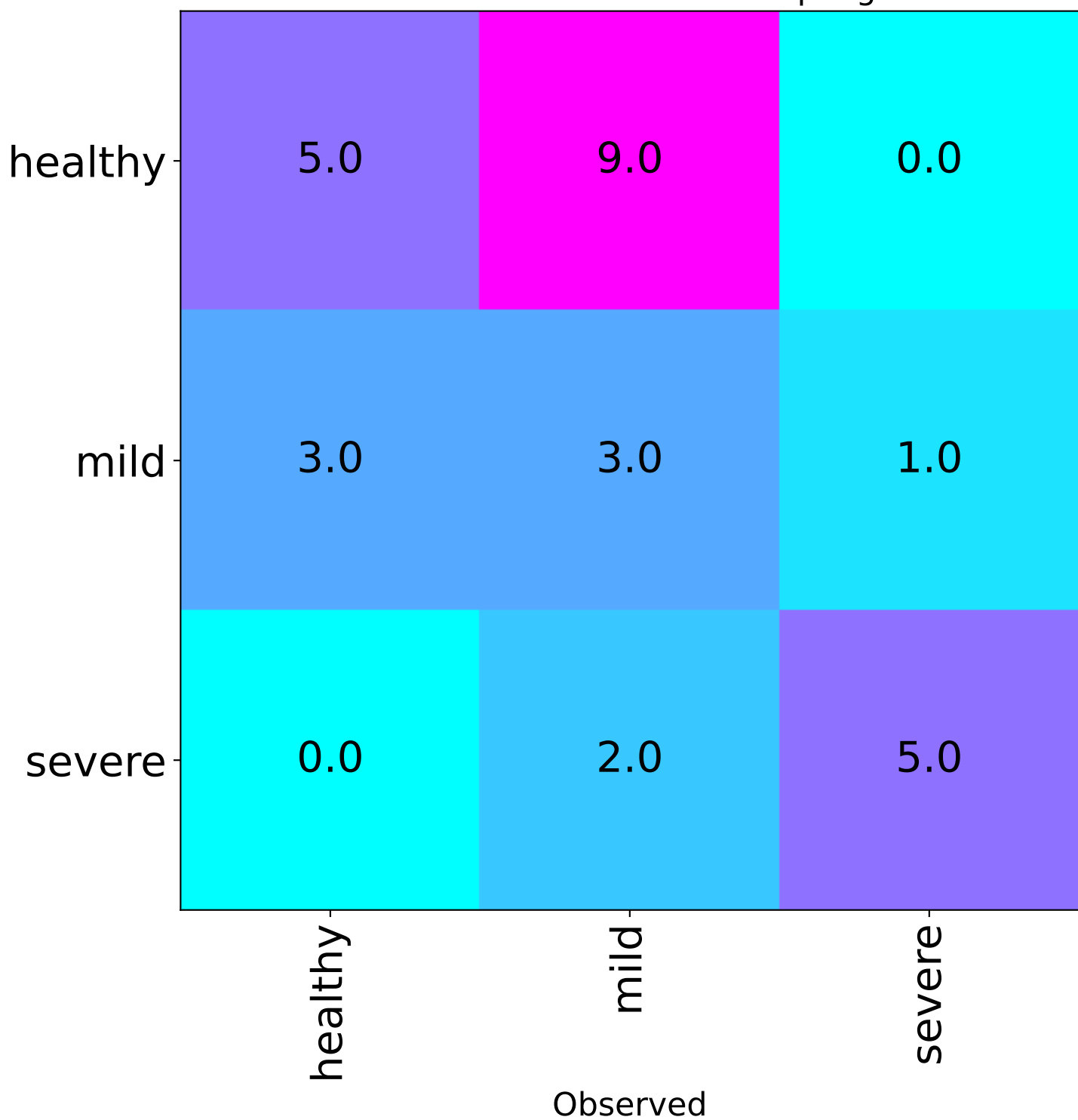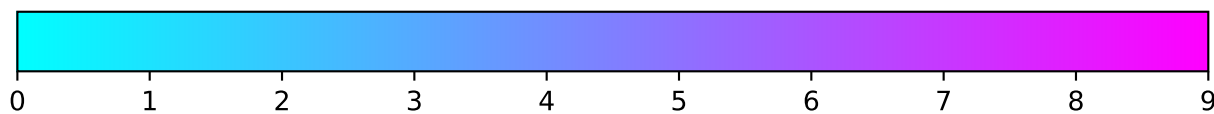

Supplement: Supplementary file 4 — Source Data [file 41467_2023_36140_MOESM4_ESM.zip › Source data/Venet Fig 2b_mDC1s_def/2022-04-28_Thu_16-58-56_GradientBoostClassifier_1/Confusion_matrix_Downsampling 7.pdf]

Confusion matrix Downsampling 6

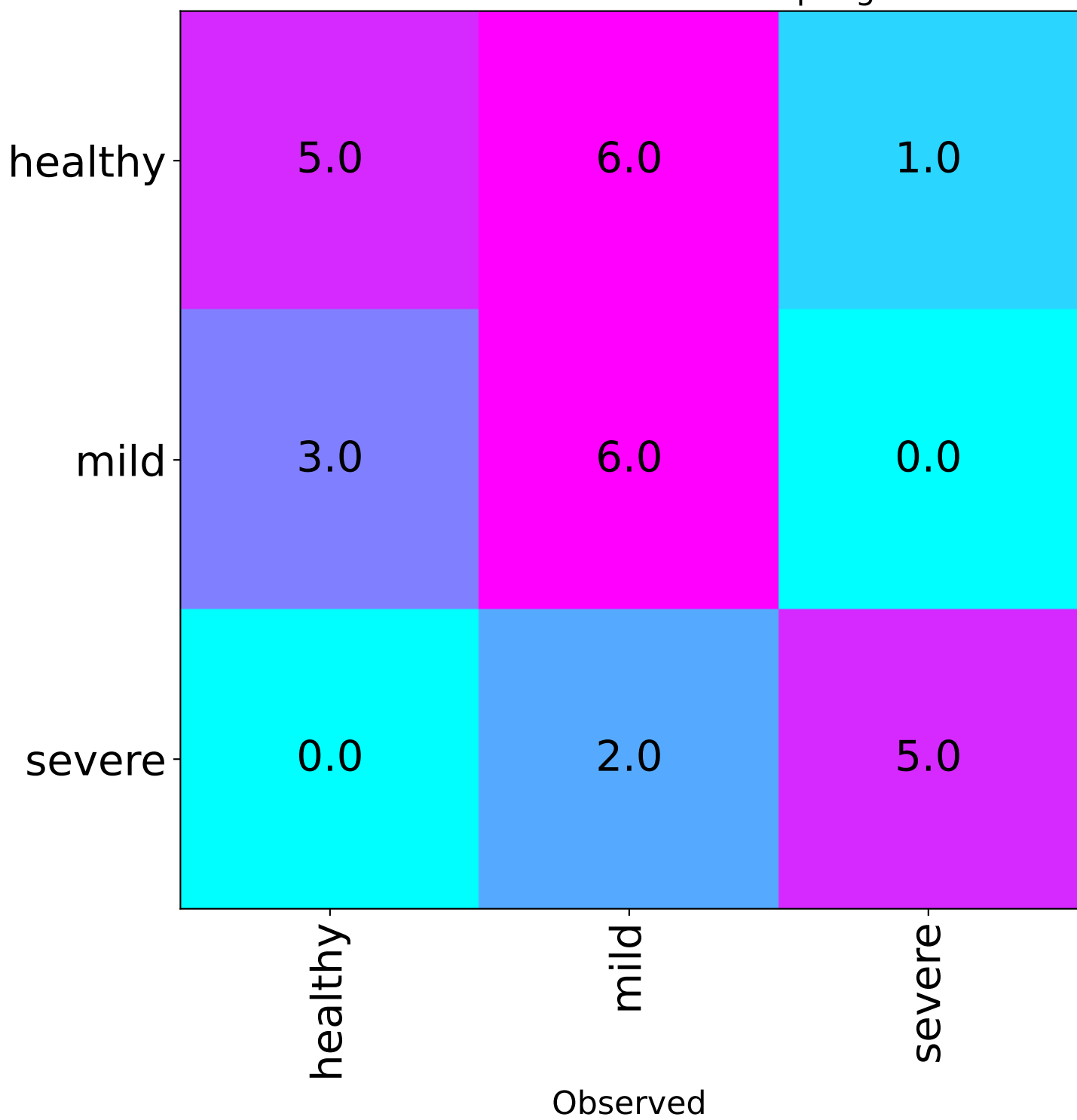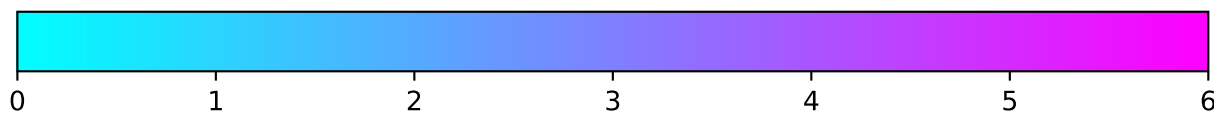

Supplement: Supplementary file 4 — Source Data [file 41467_2023_36140_MOESM4_ESM.zip › Source data/Venet Fig 2b_mDC1s_def/2022-04-28_Thu_16-58-56_GradientBoostClassifier_1/Confusion_matrix_Downsampling 6.pdf]

Confusion matrix Downsampling 4

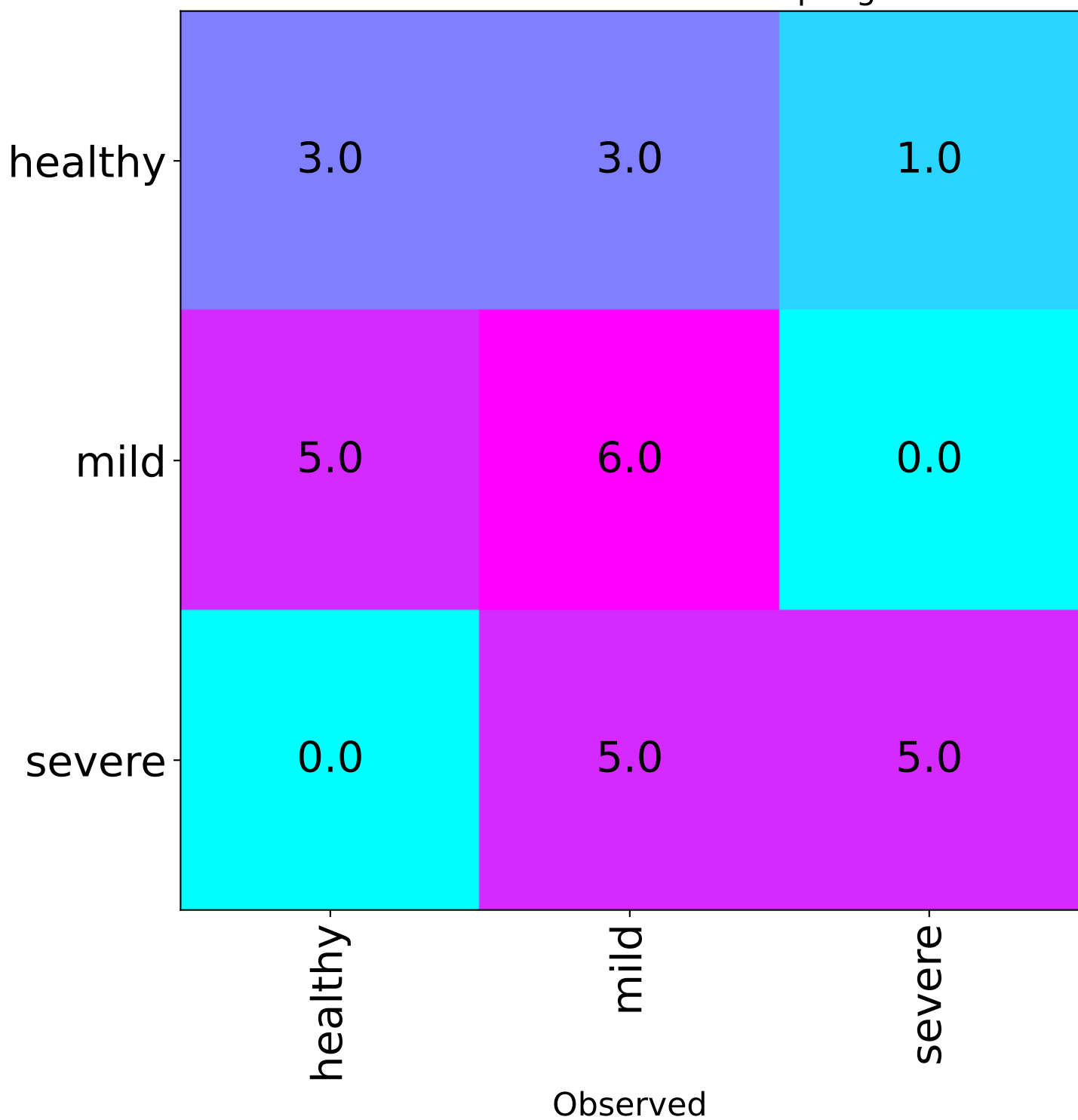

Supplement: Supplementary file 4 — Source Data [file 41467_2023_36140_MOESM4_ESM.zip › Source data/Venet Fig 2b_mDC1s_def/2022-04-28_Thu_16-58-56_GradientBoostClassifier_1/Confusion_matrix_Downsampling 4.pdf]

Confusion matrix Downsampling 5

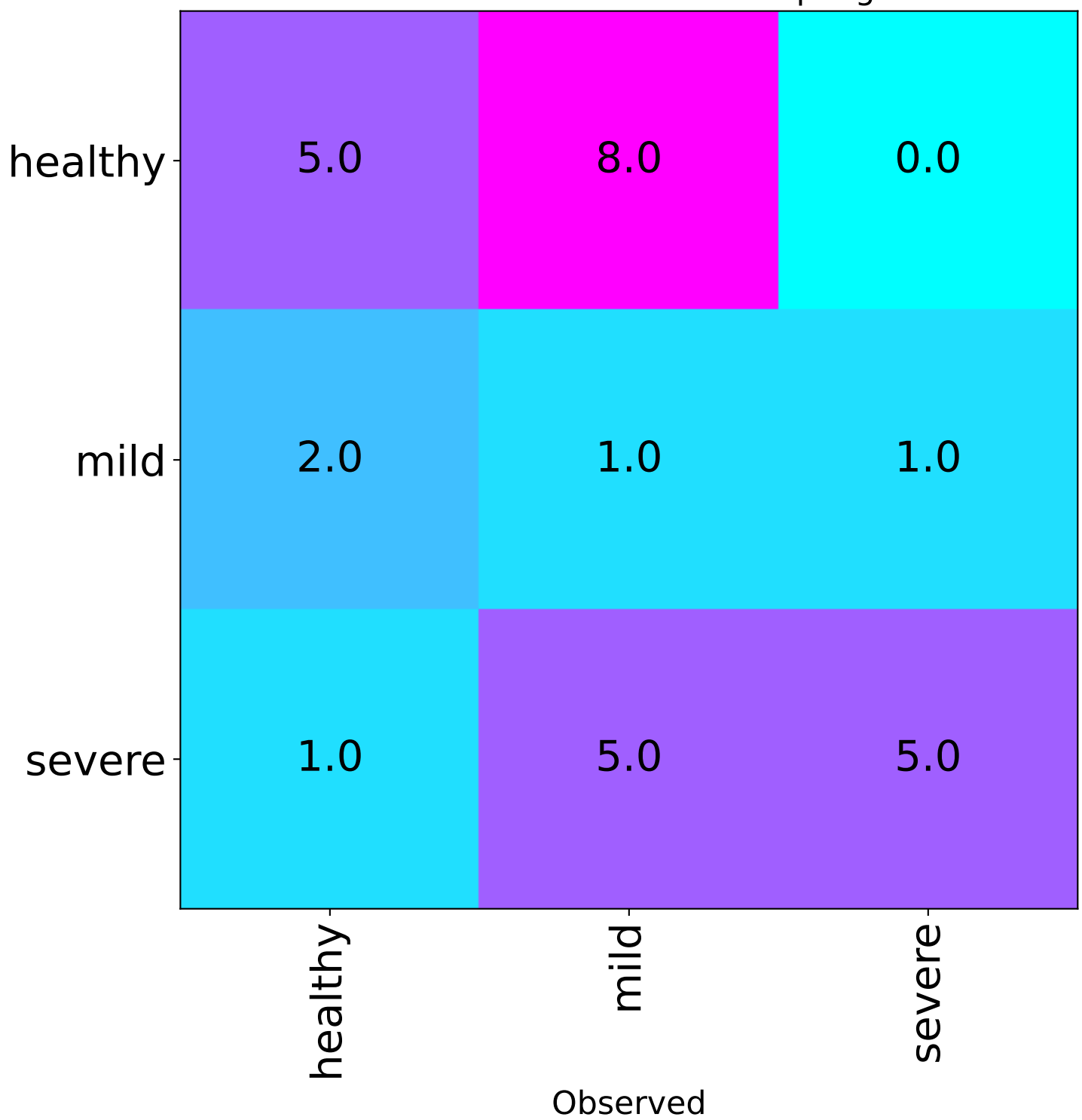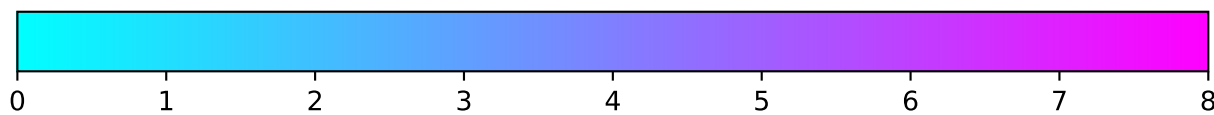

Supplement: Supplementary file 4 — Source Data [file 41467_2023_36140_MOESM4_ESM.zip › Source data/Venet Fig 2b_mDC1s_def/2022-04-28_Thu_16-58-56_GradientBoostClassifier_1/Confusion_matrix_Downsampling 5.pdf]

Confusion matrix Downsampling 1

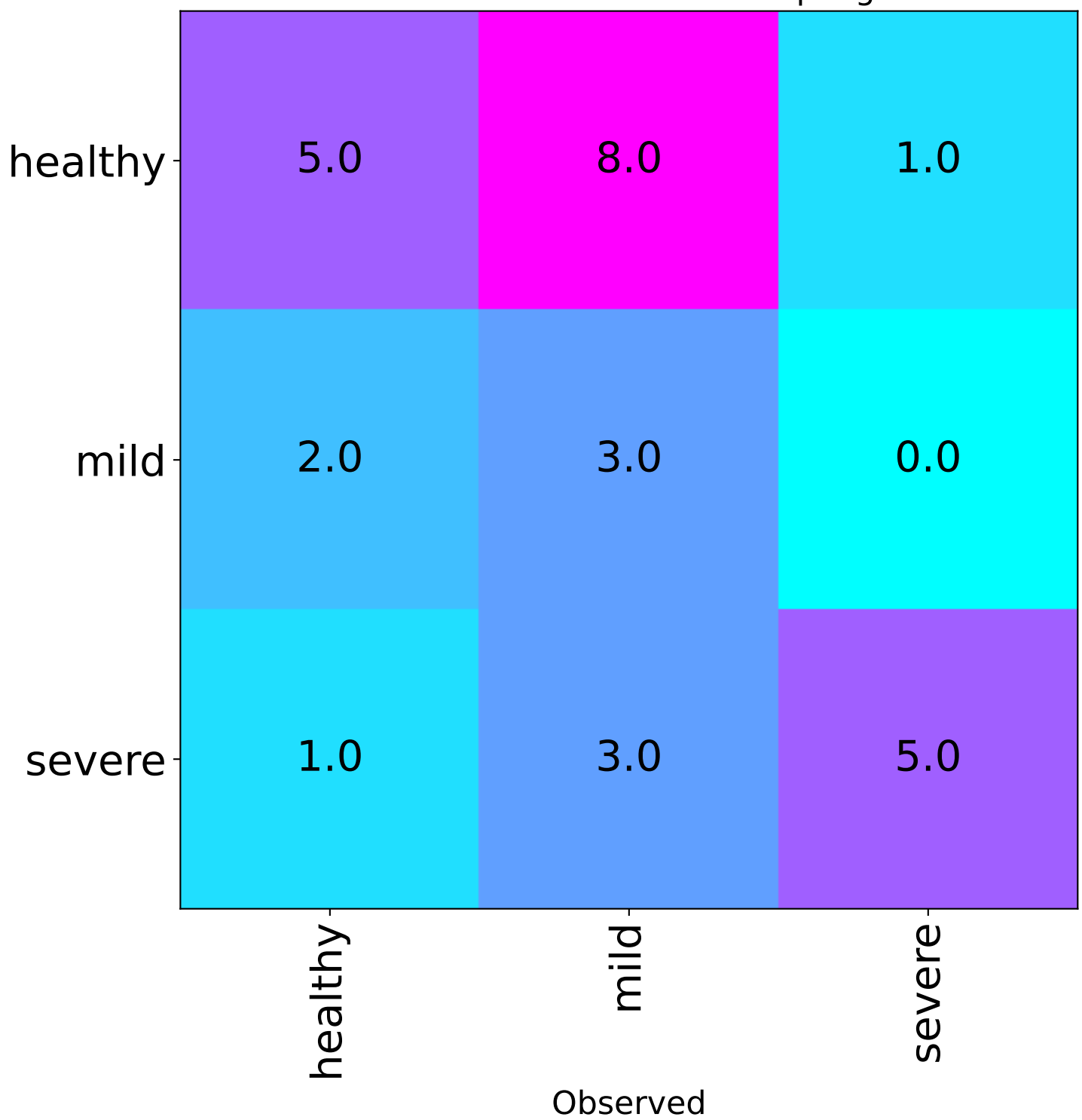

Supplement: Supplementary file 4 — Source Data [file 41467_2023_36140_MOESM4_ESM.zip › Source data/Venet Fig 2b_mDC1s_def/2022-04-28_Thu_16-58-56_GradientBoostClassifier_1/Confusion_matrix_Downsampling 1.pdf]

Confusion matrix Downsampling 0

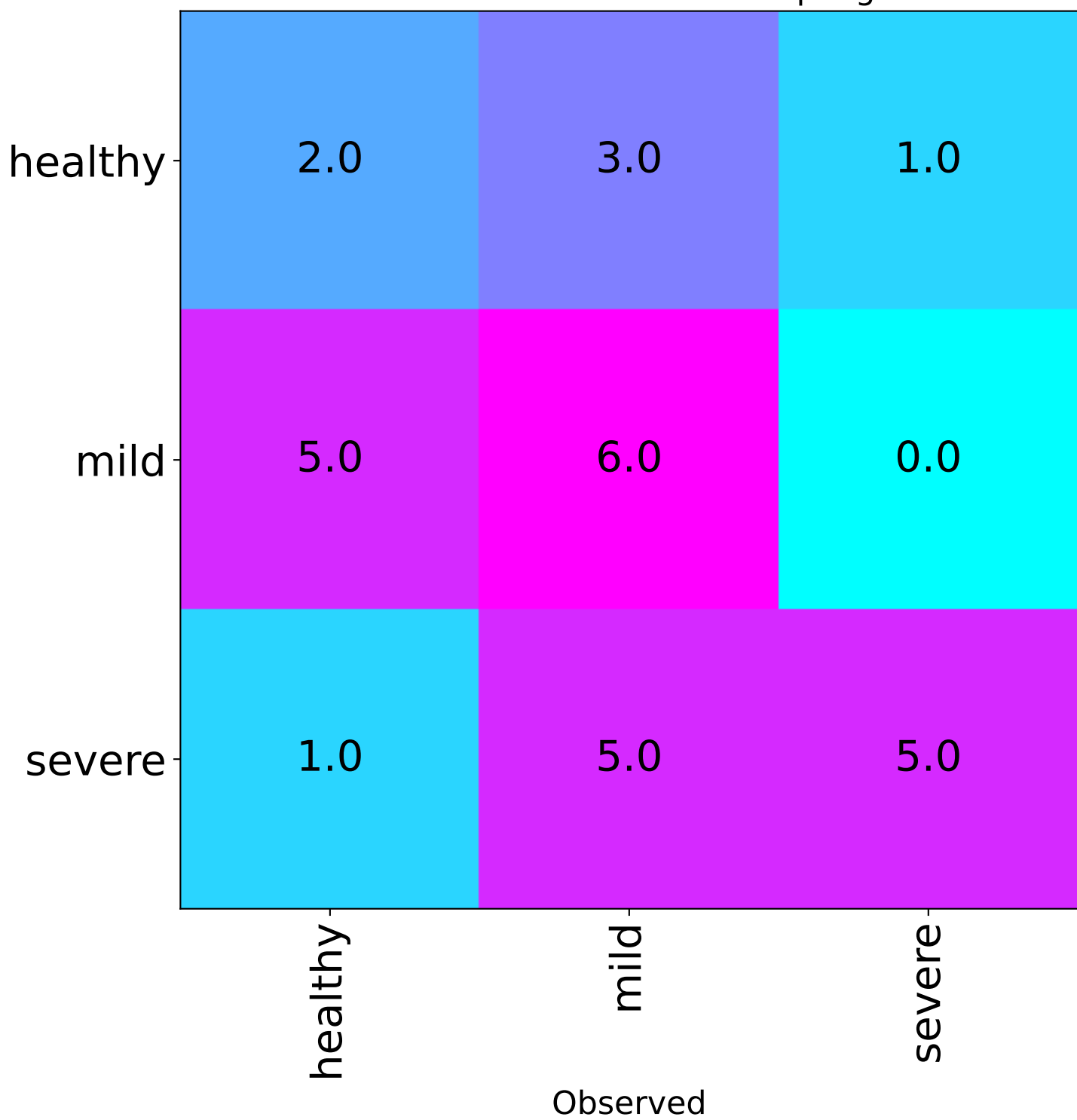

Supplement: Supplementary file 4 — Source Data [file 41467_2023_36140_MOESM4_ESM.zip › Source data/Venet Fig 2b_mDC1s_def/2022-04-28_Thu_16-58-56_GradientBoostClassifier_1/Confusion_matrix_Downsampling 0.pdf]

Confusion matrix Downsampling 2

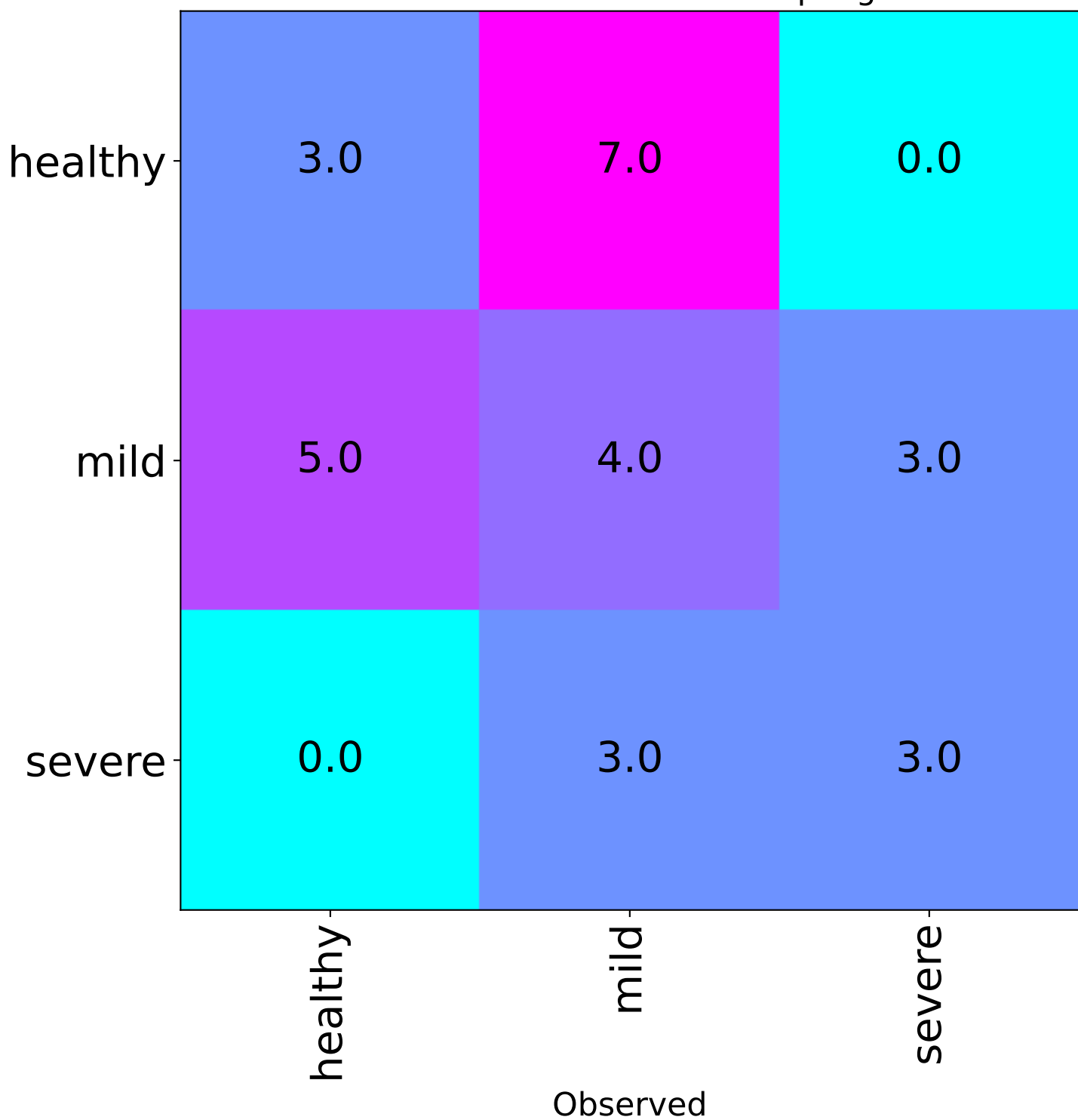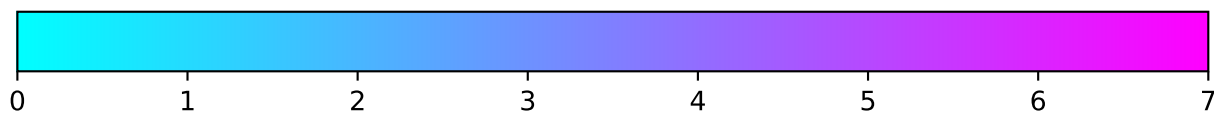

Supplement: Supplementary file 4 — Source Data [file 41467_2023_36140_MOESM4_ESM.zip › Source data/Venet Fig 2b_mDC1s_def/2022-04-28_Thu_16-58-56_GradientBoostClassifier_1/Confusion_matrix_Downsampling 2.pdf]

Confusion matrix Downsampling 3

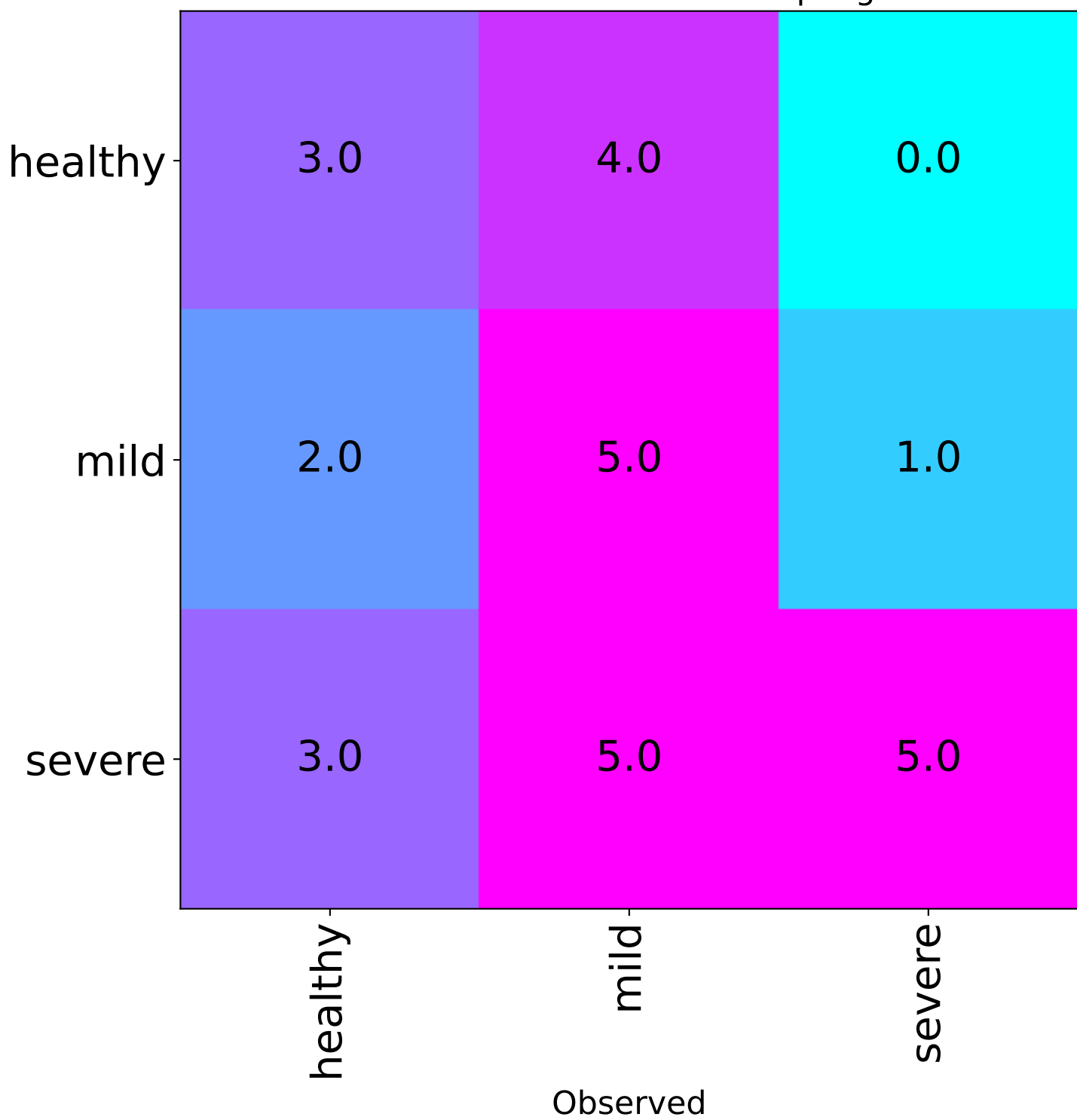

Supplement: Supplementary file 4 — Source Data [file 41467_2023_36140_MOESM4_ESM.zip › Source data/Venet Fig 2b_mDC1s_def/2022-04-28_Thu_16-58-56_GradientBoostClassifier_1/Confusion_matrix_Downsampling 3.pdf]

Confusion matrix Downsampling 8

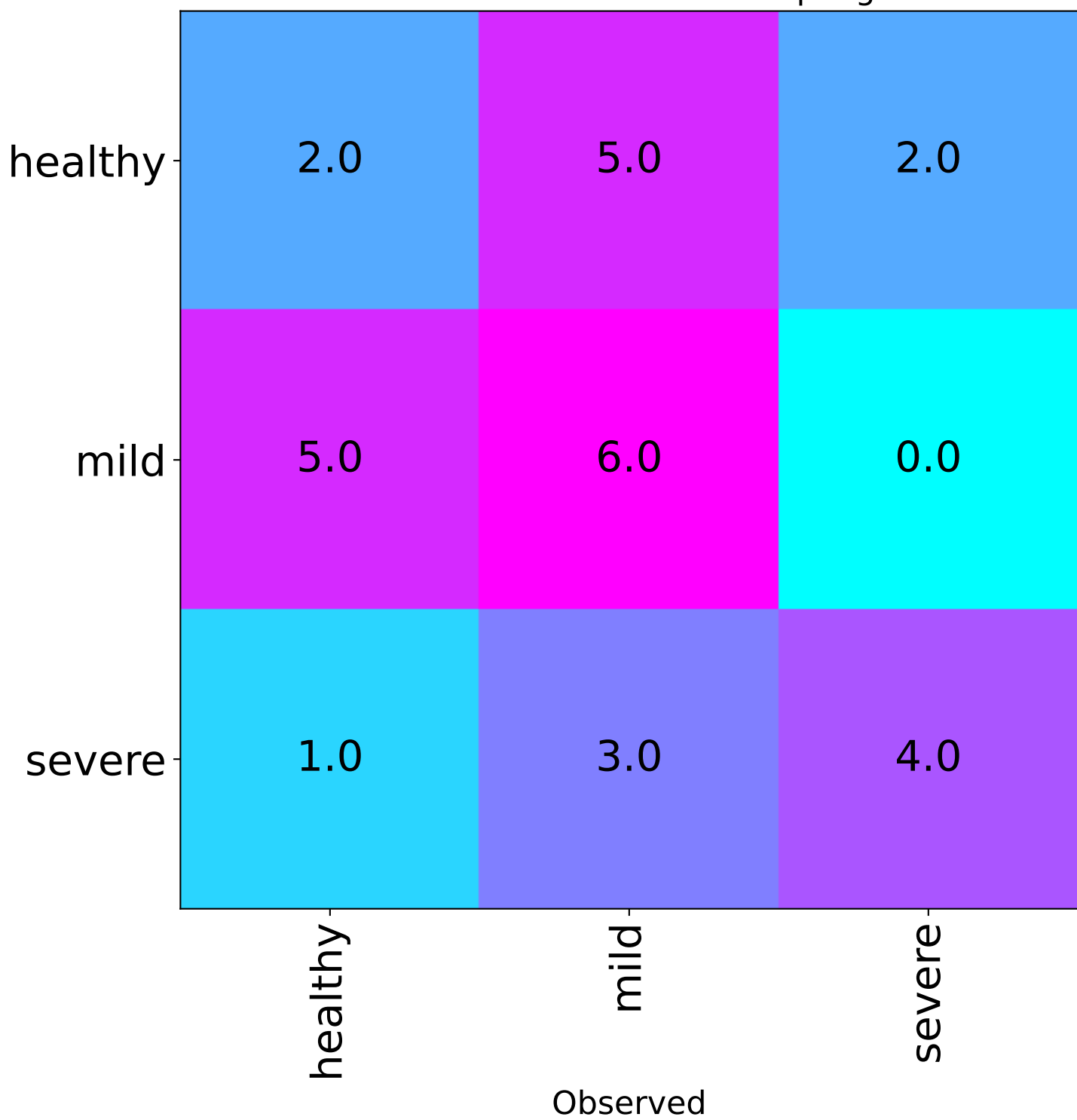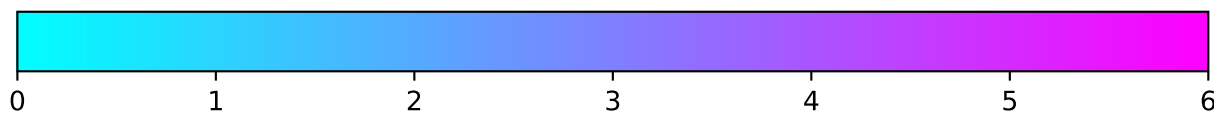

Supplement: Supplementary file 4 — Source Data [file 41467_2023_36140_MOESM4_ESM.zip › Source data/Venet Fig 2b_mDC1s_def/2022-04-28_Thu_16-58-56_GradientBoostClassifier_1/Confusion_matrix_Downsampling 8.pdf]

Confusion matrix Downsampling 9

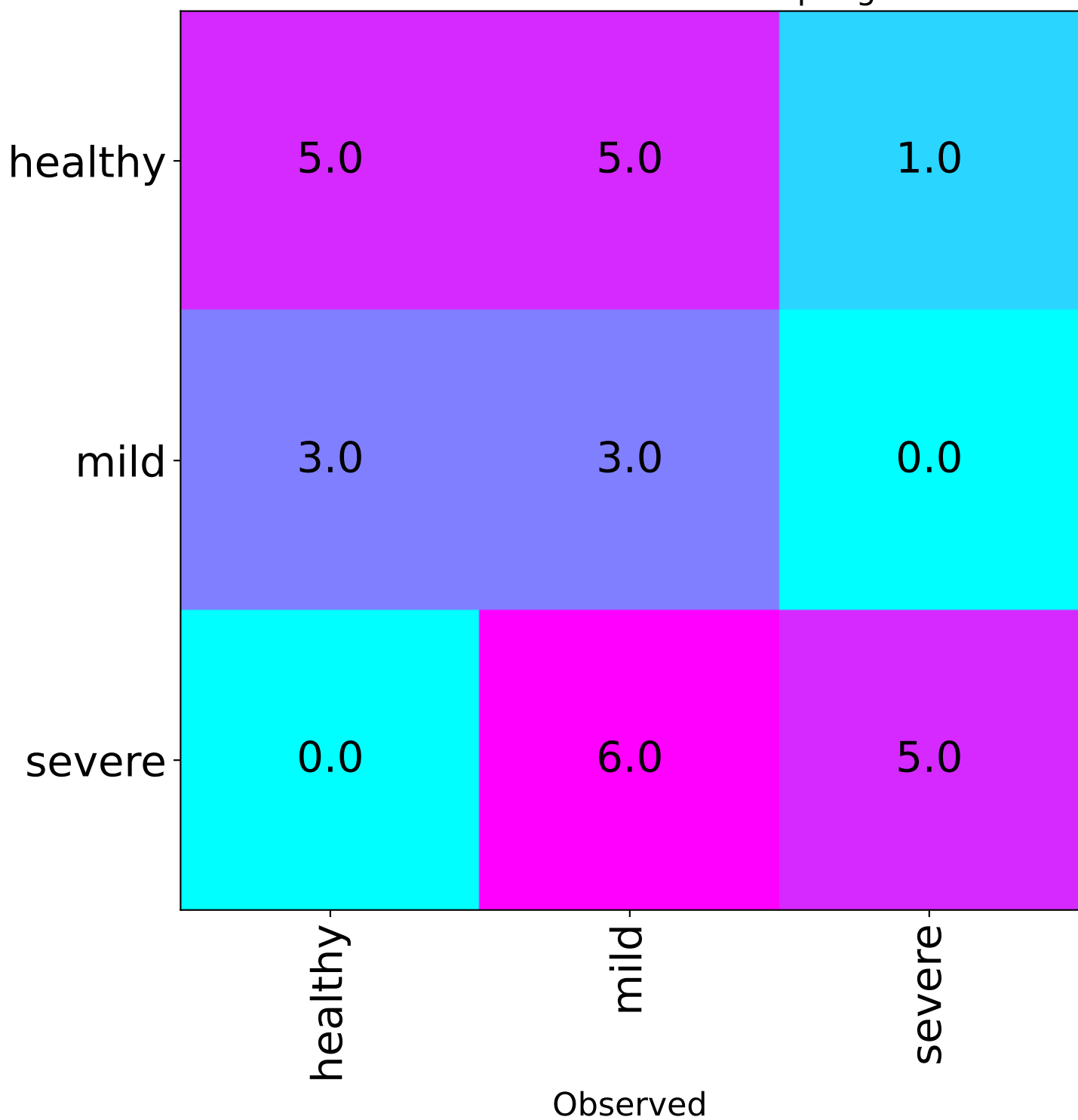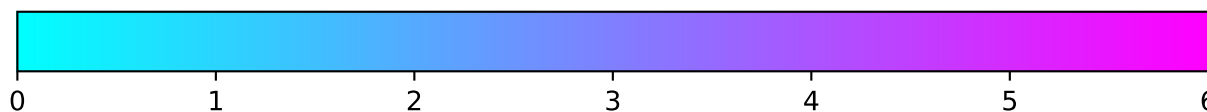

Supplement: Supplementary file 4 — Source Data [file 41467_2023_36140_MOESM4_ESM.zip › Source data/Venet Fig 2b_mDC1s_def/2022-04-28_Thu_16-58-56_GradientBoostClassifier_1/Confusion_matrix_Downsampling 9.pdf]

Confusion matrix Downsampling 7

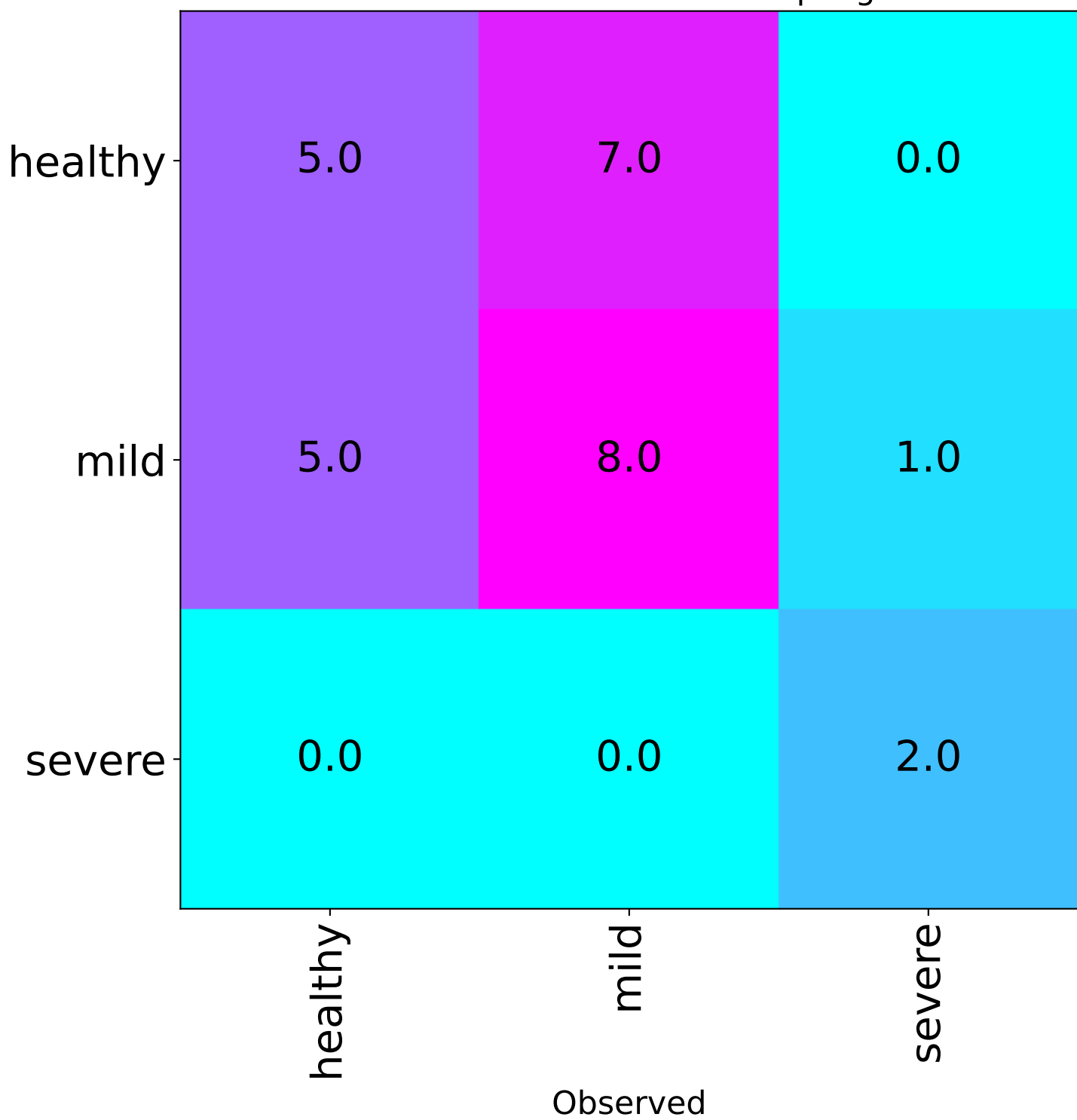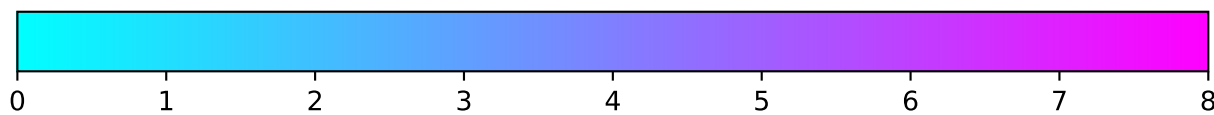

Supplement: Supplementary file 4 — Source Data [file 41467_2023_36140_MOESM4_ESM.zip › Source data/Venet Fig 2b_mDC1s_def/2022-04-28_Thu_17-00-00_GradientBoostClassifier_9/Confusion_matrix_Downsampling 7.pdf]

Confusion matrix Downsampling 6

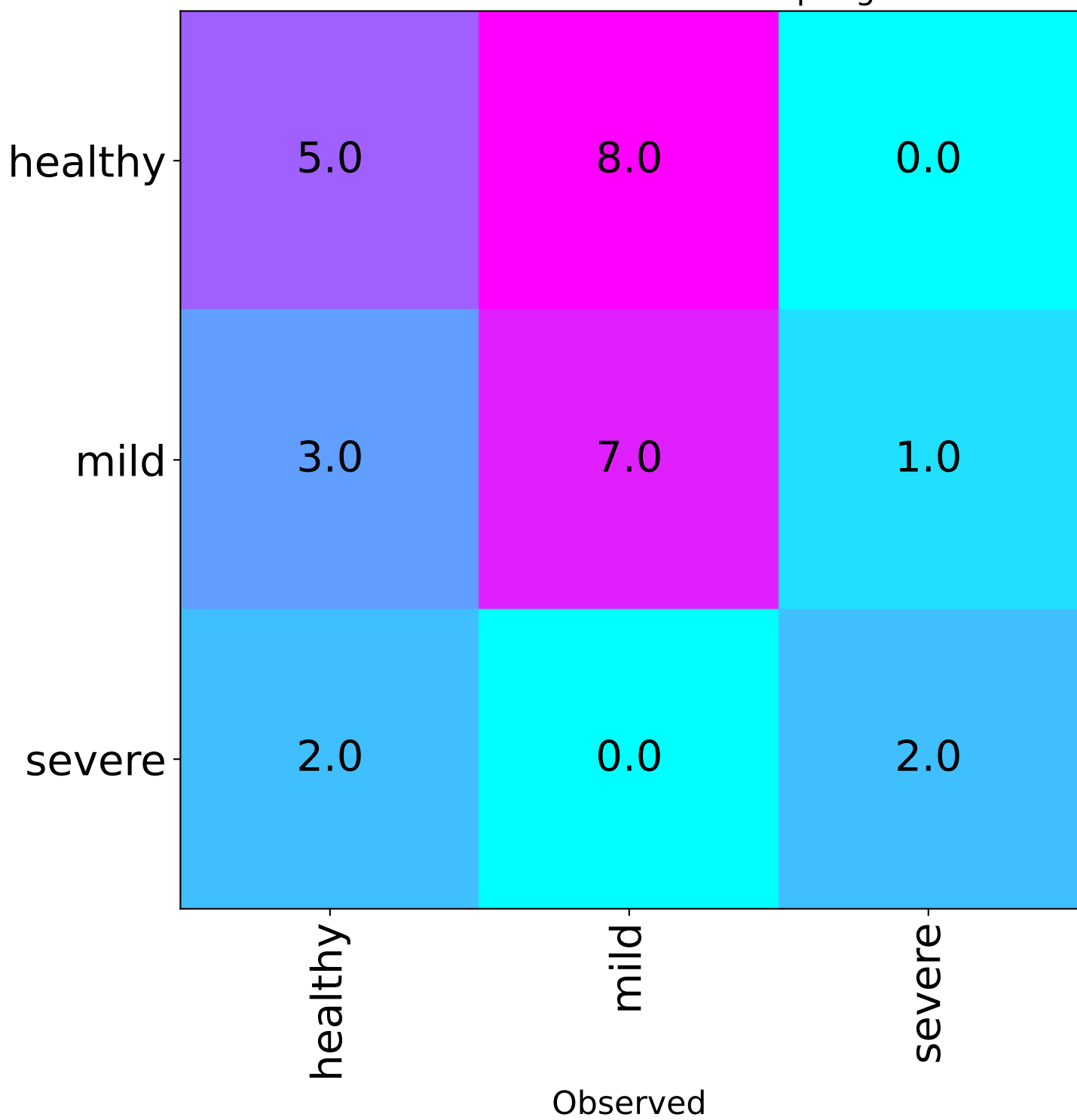

Supplement: Supplementary file 4 — Source Data [file 41467_2023_36140_MOESM4_ESM.zip › Source data/Venet Fig 2b_mDC1s_def/2022-04-28_Thu_17-00-00_GradientBoostClassifier_9/Confusion_matrix_Downsampling 6.pdf]

Confusion matrix Downsampling 4

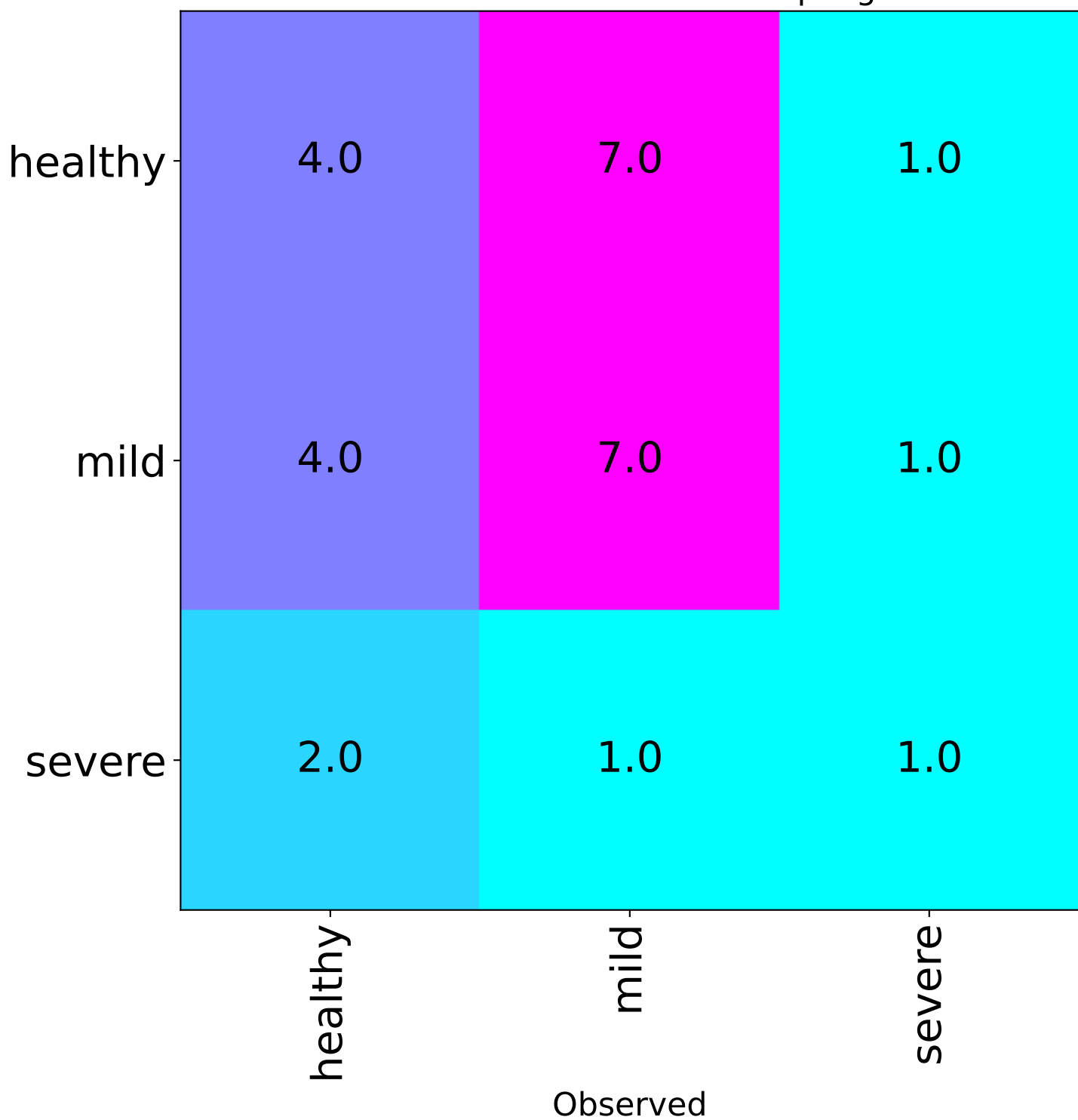

Supplement: Supplementary file 4 — Source Data [file 41467_2023_36140_MOESM4_ESM.zip › Source data/Venet Fig 2b_mDC1s_def/2022-04-28_Thu_17-00-00_GradientBoostClassifier_9/Confusion_matrix_Downsampling 4.pdf]

Confusion matrix Downsampling 5

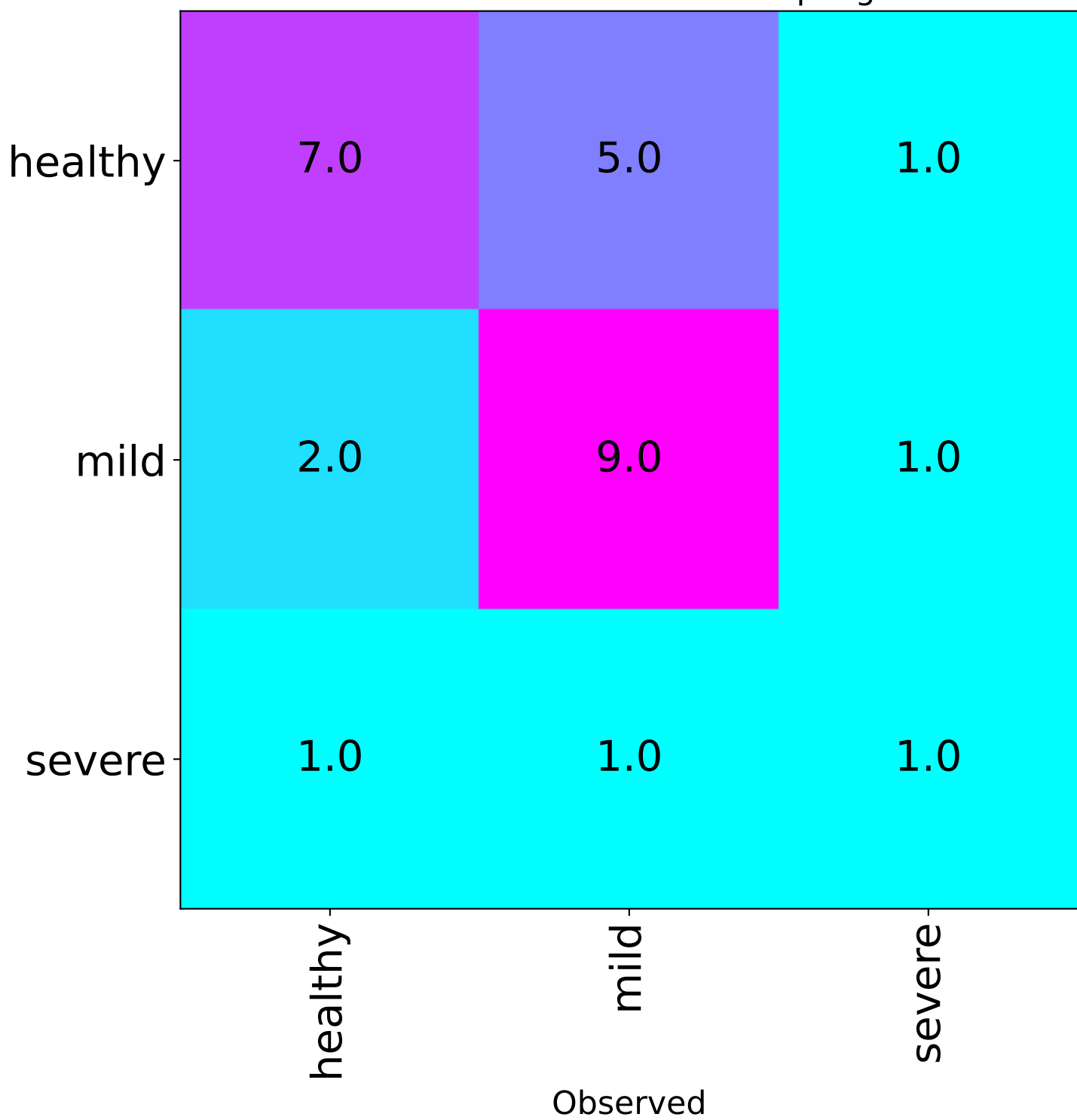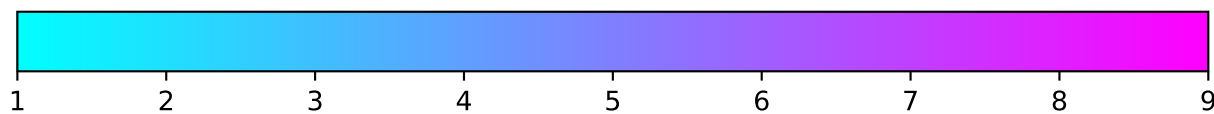

Supplement: Supplementary file 4 — Source Data [file 41467_2023_36140_MOESM4_ESM.zip › Source data/Venet Fig 2b_mDC1s_def/2022-04-28_Thu_17-00-00_GradientBoostClassifier_9/Confusion_matrix_Downsampling 5.pdf]
